# Symptoms after Ingestion of Pig Whipworm *Trichuris suis* Eggs in a Randomized Placebo-Controlled Double-Blind Clinical Trial

**DOI:** 10.1371/journal.pone.0022346

**Published:** 2011-08-02

**Authors:** Peter Bager, Christian Kapel, Allan Roepstorff, Stig Thamsborg, John Arnved, Steen Rønborg, Bjarne Kristensen, Lars K. Poulsen, Jan Wohlfahrt, Mads Melbye

**Affiliations:** 1 Department of Epidemiology Research, Statens Serum Institut, Copenhagen, Denmark; 2 Department of Agriculture and Ecology, Faculty of Life Sciences, University of Copenhagen, Frederiksberg, Denmark; 3 Department of Veterinary Disease Biology, Faculty of Life Sciences, University of Copenhagen, Frederiksberg, Denmark; 4 Pulmonology and Allergy Clinic of Copenhagen, Copenhagen, Denmark; 5 Phadia ApS, Allerød, Denmark; 6 National University Hospital, Allergy Clinic, Copenhagen, Denmark; Statens Serum Institute, Denmark

## Abstract

Symptoms after human infection with the helminth *Trichuris suis* have not previously been described. Exposure to helminths has been suggested as immune therapy against allergy and autoimmune diseases. We randomized adults with allergic rhinitis to ingest a dose of 2500 *T. suis* eggs or placebo every 21 days for 168 days (total 8 doses) in a double-blind clinical trial. In a previous publication, we reported a lack of efficacy and a high prevalence of adverse gastrointestinal reactions. The aim of the present study was to present a detailed description of the adverse event data and post-hoc analyses of gastrointestinal reactions. Adverse events and severity (mild, moderate, severe) were recorded daily by subjects, classified by organ using MedDRA 10.0, and event rates compared between subjects on *T. suis* treatment vs. subjects on placebo. *T. suis*-specific serum IgG antibodies were measured by a fluoroenzymeimmunoassay (Phadia ApS). During 163 days complete follow-up, subjects ingesting *T. suis* eggs (N = 49) had a three to 19-fold higher rate of events (median duration, 2 days) with gastrointestinal reactions (moderate to severe flatulence, diarrhea, and upper abdominal pain) compared with placebo subjects (N = 47). The highest incidence of affected subjects was seen from the first few days and until day 42 (3^rd^ dose): 63% vs. 29% for placebo; day 163: 76% vs. 49% for placebo. Seroprevalences increased concurrently in the *T. suis* group: Day 59, 50%; day 90, 91%; day 170, 93%. The combined duration of episodes with onset before day 42 was ≤14 days in 80% of affected subjects. Age, gender, total IgE, and recent intestinal symptoms at baseline did not predict gastrointestinal side effects. In conclusion, during the first 2 months, repeated ingestions of 2500 *T. suis* eggs caused frequent gastrointestinal reactions lasting up to 14 days, whereas 4 months further treatment mainly provoked a subclinical stimulation.

**Trial registration:**

University hospital Medical Information Network trial registry Reg. no. R000001298, Trial ID UMIN000001070.

## Introduction

The whipworm *Trichuris suis* is a common mild intestinal pathogen of pigs, which is able to establish temporarily in the human caecum and colon [1], [2]. Ingestions of live *T. suis* eggs was reported to be effective in treating patients with inflammatory bowel disease (IBD) in two clinical trials [3]–[6]. The rationale for such therapy stems from the so-called hygiene hypothesis [6], and observations of a reduced pathology in chronic helminth infections, which is believed to be the results of immune-suppression by regulatory T cells, cytokines (IL-10, TGF-β), IgG4 replacing IgE, or other mechanism [7]. In experimental studies, helminths can suppress disease activity in models of IBD as well as of allergy, asthma, multiple sclerosis, type I diabetes, and arthritis [8]–[11]. However, treatment of patients with allergic rhinitis using *T. suis* infections, or patients with asthma or IBD using hookworm infections, did not suppress disease activity in recent clinical trials [12]–[14]. Withstanding the reported therapeutic effect of *T. suis* on IBD patients, similar treatment of other chronic inflammatory diseases is relevant to put on trial [15].

Ingestion of live *T. suis* eggs has been advocated as safe to humans. For example, the life cycle of *T. suis* is not associated with auto-reinfection, direct person to person infection, aberrant migration, or hypobiosis [1], [3]. There has, however, not been published any reports of adverse events other than case-reports [16]–[19]. Recently we performed a randomized controlled clinical trial of *T. suis* therapy against allergic rhinitis in otherwise healthy subjects [12]. We found no efficacy but *T. suis* antibodies (92%) and eosinophilia (41%) which confirmed establishment of *T. suis*. We reported that gastrointestinal reactions were common and occurred at a higher prevalence in the *T. suis* group than the placebo group. However, the prevalence data underlying this conclusion was not described in detail. Our aim in this study was to describe the adverse event data in detail (e.g. types of data sources and distributions by organ groups), and to perform post-hoc analyses to determine the incidence, severity, rate, duration and potential risk factors for gastrointestinal symptoms after human ingestion of whipworm *T. suis* eggs.

## Methods

The protocol and original statistical plan for this trial, as well as the CONSORT checklist are available as supporting information; see checklist S1 and protocol S1.

### Subjects and study design

The study was performed in accordance with the Declaration of Helsinki [20] and Good Clinical Practice (GCP) and was approved by an independent review board of the Danish Ethics Committees (Reference no. H-KF-2006-4100). Written informed consent was obtained before enrolment. The study was a randomized placebo-controlled double-blinded single-center clinical trial conducted in the capital of Denmark, as described in detail elsewhere [12]. Briefly, enrolled subjects were age 18 to 65 years with grass pollen-induced allergic rhinitis, no IBD, and no childbearing potential. Subjects were randomized to receive eight treatments with placebo or *T. suis* eggs with an interval of 21 days. The trial consisted of nine visits scheduled over 168 days, spaced by 21 days (±3) and including eight treatments visits starting at visit one, and three blood sampling visits scheduled at visit one (blood visit one), one of visit three to six (blood visit two, scheduled during the grass pollen season), and at visit nine (blood visit three). Enrolment (visit one) of subjects took two months.

Analyses of blood were performed as described elsewhere [12] and included measurements of serum antibodies by a fluoroenzymeimmunoassay (ImmunoCAP™, ISO 13485; Phadia ApS, Allerød, Denmark), total histamine from cell lysis (RefLab ApS, Copenhagen, Denmark) [21], and haematology (Copenhagen GP Laboratory, ISO 17025, Copenhagen, Denmark). Seroconversion for *T. suis* infection was defined as a *T. suis*-IgG level above the mean of levels measured in a cohort of 15 non-atopic donors who neither participated in the trial nor received *T. suis* eggs. The mean was denoted the cut-off level.

### Agent and intervention

The active agent consisted of embryonated *T. suis* eggs, which were isolated from pigs by Parasite Technologies A/S, Copenhagen, Denmark, and processed to vials by Ovamed GmbH, Barsbüttel, Germany. Each vial was specified to contain 2500 embryonated eggs, or no eggs (placebo), in 15 ml liquid, to be used for one oral treatment. With timely intervals of one to two months, a bulk of liquid with eggs (five bulks in total) were filled onto vials that were delivered to the trial site, as described elsewhere [12]. The numbers of embryonated eggs, counted by quality-controlled microscopy, in randomly selected vials from bulk one to five were 2310, 2010, 2355, 2400, and 2400, respectively. Vials were traceable to bulks by a labeling with blinded numbers indicating subject and bulk number. Placebo was identically supplied and formulated except that it contained no *T. suis* eggs. On scheduled treatment dates (+/− three days), administration was performed by drinking directly from the vial. Subjects were instructed that the vial's content should be intaken on empty stomach.

### Adverse events

Information on adverse events was recorded in diaries kept by subjects daily, and in case report forms (CRF) kept by two doctors and two nurses who evaluated events at each visit. The diaries and CRFs were designed for the study with preprinted guide, examples, baseline questions, and daily questions.

In the diary, subjects spontaneously (unsolicited) recorded the name of any event of importance for their health (>100 recording spaces). However, with the exception of “flatulence”, “diarrhea”, and “pruritus ani” because these were preprinted in the diary to systematically collect data on severity of symptoms that also occur during pig *T. suis* or human *T. trichiura* infections. Subjects then scored the severity of events by ticking off one of four numbers (0, 1, 2, 3) for the three weeks preceding 1^st^ treatment, and *daily* during trial. Subjects were guided only by the diary on how to grade the numbers: “0 = No symptom, 1 = mild symptom (easily tolerated), 2 = moderate symptom (troubles activities), and 3 = severe symptom (hinders activities)”. After completing a two-week period subjects also ticked off the treatment they currently believed they received (active, placebo, or “I do not know”), and reasons they believed so (“I can guess it based on the doctor”, “The treatment affects me”, “The treatment does not affect me”, other).

In the CRF, the nurses recorded event name, start and end date, maximum severity, and relatedness to treatment. This specific information was obtained at the clinical visits, by the doctor and then the nurse during interview with the subject and inspection of his/hers diary. For guidance on which events should be recorded in such detail, the doctor/nurse were first to record for each visit whether the subject had experienced (1) moderate to severe flatulence, diarrhea, and pruritus ani (yes/no), (2) serious events (hospitalization) (yes/no), and (3) other events judged to be important for the subject's health (yes/no). If any of these questions were answered in the affirmative, then the nurse should record the events in detail. Because moderate to severe flatulence, diarrhea, and pruritus ani were expected to occur spontaneously without clinical importance, the nurses were guided to record such events in detail only if they appeared unexpected, by way of example “if a subject e.g. has more than seven days of moderate diarrhea distributed over three weeks”.

Using the medical software dictionary MedDRA® version 10.0 (International Federation of Pharmaceutical Manufacturers and Associations, Geneva, Switzerland), Danish event names were assigned a corresponding English Preferred Term (PT) from MedDRA®. The PT events were included in analyses if treatment-emergent, i.e. not seen in the three weeks before enrolment or worsened even if present in those three weeks. The System Organ Class (SOC) for each event was extracted from medDRA®. For groups or SOCs of events the maximum daily severity was chosen for analyses.

### Statistical analyses

Differences between the *T. suis* and placebo group in the rate of adverse outcomes were evaluated by event rate ratios (RR) and estimated by Cox regression using the PHREG procedure in SAS (version 9.1.3., Cary, USA). Each subject was followed from start of trial and end of each episode of the relevant adverse event until day of the next relevant adverse event or their stop of diary, whichever came first. Stop of diary was defined as the last day with complete recording (no missing values) for allergic rhinitis symptoms and medications (10 values) [12]. The date of the stop day corresponded well with the last date memorized by electronic pocket peak-flowmeters that all subjects blew into at least once every week. Inter-subject correlation was taken into account by using a variance sandwich estimator. Test for effect modification by subject characteristics were performed by including an interaction term in the regression. Differences between the groups in duration of events were tested by non-parametric log rank test using the LIFETEST procedure in SAS. Within the *T. suis* group, differences between trends in blood parameters over time in subjects with none or mild vs. moderate or severe symptoms before the time of the blood sampling were tested by linear regression using the GENMOD procedure in SAS with inter-subject correlation taken into account by Generalized Estimating Equation. Association between self-evaluated and true treatment allocation was evaluated by chi-square statistics. Missing values were disregarded. All tests were two sided using a significance level of 5%.

## Results

### Study flow, subject characteristics and blinding

After randomization, allocation, and receival of first treatment with *T. suis* eggs (N = 50) and placebo (N = 50), 1 subject in the *T.suis* group discontinued the study due to move abroad, 2 subjects in the placebo group dropped out due to no time/interest, and 1 subject in the placebo group was withdrawn because his wife filled in the diary. The study thus included 49 subjects on *T. suis* and 47 on placebo. Subjects recorded diary for on average of 163 days (min. 68, Q1 167, median 168, Q3 168, max. 168) out of the 168 days the trial lasted per subject. The characteristics of subjects were similar between the treatment groups, as reported previously [12] and for additional characteristics in Table 1. Treatment allocation was blinded (i.e. to doctors, nurses, subjects, sponsor, and other study personal) and the percentage of subjects who believed they received *T. suis* (37% vs. 32% for placebo; P = 0.62), or the subset recording they believed so based on the doctor (n = 1 vs. n = 0 for placebo), was not significantly different between the treatment groups.

**Table 1:** Baseline characteristics by treatment group in 100 subjects with grass-pollen induced allergic rhinitis in a randomized placebo-controlled double-blind clinical trial of *T. suis* for grass-pollen allergy, Denmark, 2008.

	*T. suis*	Placebo
	N = 50	N = 50
**Sex**, n (%)		
Male	48 (96)	47 (94)
Female	2 (4)	3 (6)
**Caucasian**, n (%)	50 (100%)	50 (100%)
**Age** (years)		
Mean (SD)	35 (10)	39 (10)
Male	34 (9)	38 (10)
Female	60 (3)	51 (13)
Minimum-maximum	20–61	19–63
**Allergic rhinitis**	50 (100%)	50 (100%)
**Duration of allergic rhinitis** (years), mean (SD)	20 (11)	24 (12)
**Body mass index** (kg/height^2^), median	24.7*	25.6
Minimum-maximum	19.5–39.2*	19.7–44.3
**Total IgE** (kU/l), median	72.9	70.3
Minimum-maximum	20.7–620.6	13.5–1206

*One subject was missing information on height.

### Gastrointestinal symptoms

**Figure 1:**
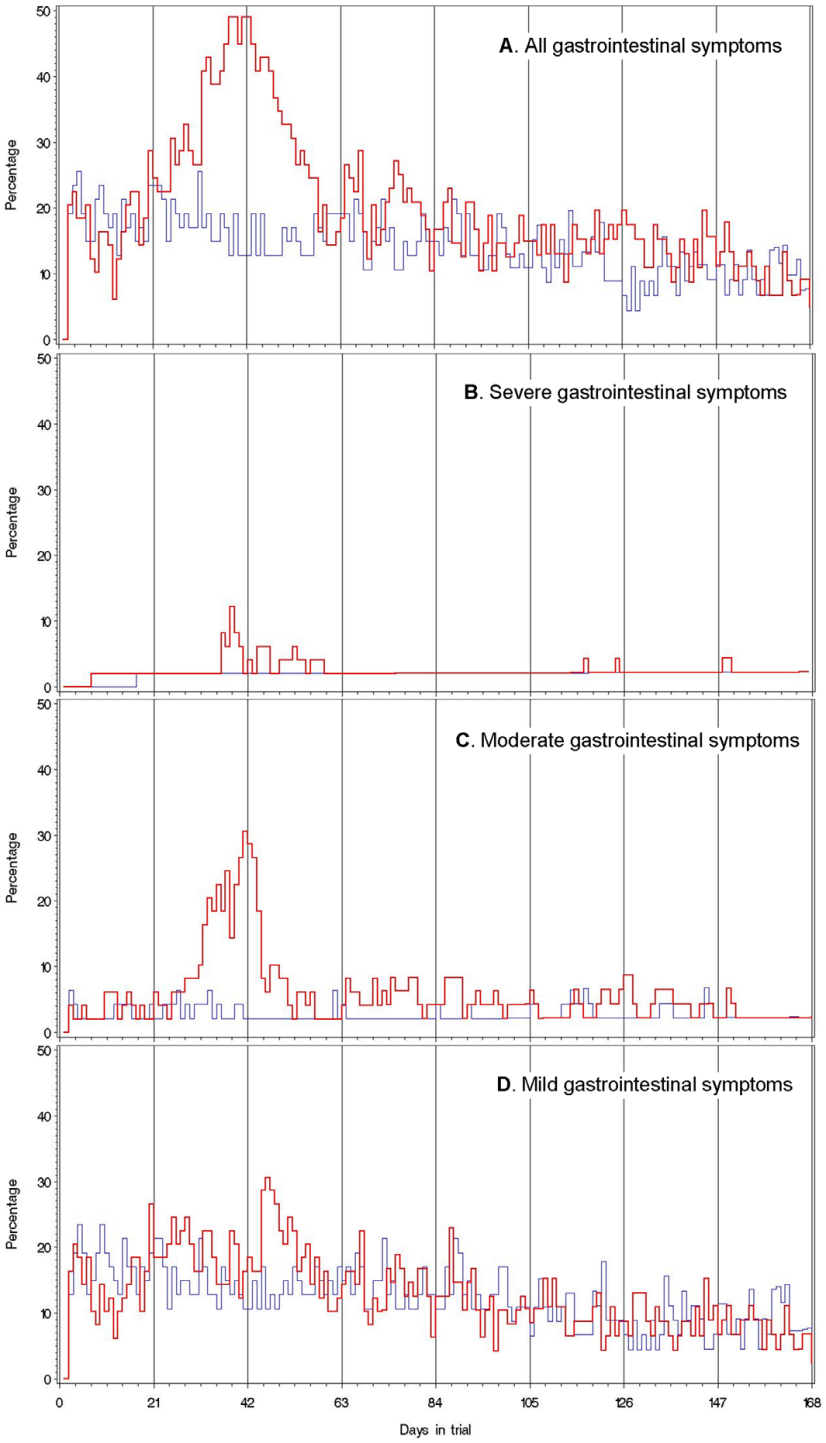
Daily prevalence of subjects who reported gastrointestinal symptoms. (A) by number of days participating in a randomized double-blind clinical trial of three-weekly ingestions of infective *T. suis* eggs (N = 49, red/bold line) and placebo (N = 47, blue/grey line), along with corresponding figures for subjects who reported severe (B), moderate (C) and mild (D) gastrointestinal symptoms, Denmark, 2008. Vertical lines represent days of clinic visits (three-weekly) when subjects were to ingest 2500 live *T. suis* eggs or placebo except on visit day 168 (total 8 doses).

Table 2 presents the rate ratio of self-recorded and doctor/nurse-reported adverse events by organ class and treatment group. Overall, only moderate to severe gastrointestinal symptoms occurred consistently at a significantly higher rate in the *T. suis* group when compared with the rate in the placebo group (*Self-recorded*, RR 2.1, 95% CI 1.2–3.5; *Doctor/nurse-recorded*, RR 2.7, 95% CI, 1.5–5.2). Figure 1 presents the daily prevalence of gastrointestinal symptoms by treatment group and gradings of severity of gastrointestinal symptoms (mild, moderate, severe, and any severity). Overall, *mild* gastrointestinal symptoms occurred at a daily prevalence of about 10–20% in both treatment groups on the majority of days during the trial (Figure 1A) while moderate and severe symptoms were characteristic of the *T. suis* group (Figure 1B and 1C). Table 3 presents the rate ratio of gastrointestinal adverse events according to maximum severity and type by days and treatment group. Overall, the rate of mild gastrointestinal symptoms was not significantly different between the treatment groups (Table 3). In contrast, the rate of moderate to severe gastrointestinal symptoms in the *T. suis* group was significantly higher than in the placebo group (Table 2 and 3).

**Table 2:** Rate ratios (RR) for first adverse event according to System Organ Class by treatment group in a randomized double-blind clinical trial of *T. suis* and placebo, Denmark, 2008.

	First adverse events recorded by subject						First adverse event recorded by doctors/nurses					
	Any severity					Moderate to severe	Any severity					Moderate to severe
	*T. suis*		Placebo				*T. suis*		Placebo			
	N = 49		N = 47				N = 49		N = 47			
	n	(%)	n	(%)	RR (95% CI)	RR (95% CI)	n	(%)	n	(%)	RR (95% CI)	RR (95% CI)
**Any adverse event**	44	(90)	40	(85)	1.2 (0.8–1.8)	1.2 (0.6–2.4)	43	(88)	36	(77)	1.6 (1.0–2.5)	1.8 (1.1–3.0)
Gastrointestinal disorders	43	(88)	34	(72)	1.4 (0.9–2.2)	2.1 (1.2–3.5)	37	(76)	24	(51)	2.0 (1.2–3.4)	2.8 (1.5–5.2)
Nervous system disorders	15	(31)	19	(40)	0.7 (0.4–1.4)	0.8 (0.4–1.7)	18	(37)	18	(38)	0.9 (0.5–1.7)	0.8 (0.3–2.1)
Respiratory, thoracic and mediastinal disorders	12	(25)	7	(15)	1.8 (0.7–4.6)	1.7 (0.6–4.7)	11	(22)	9	(19)	1.2 (0.5–2.9)	1.1 (0.4–3.4)
Skin and subcutaneous disorders*	10	(20)	2	(4)	5.2 (1.1–23.5)	3.5 (0.7–16.7)	9	(18)	3	(6)	3.1 (0.8–11.4)	3.9 (0.4–35.2)
Musculoskeletal and connective disorders†	7	(14)	2	(4)	4.1 (0.8–20.2)	7.1 (0.8–60.0)	7	(14)	5	(11)	1.3 (0.4–4.2)	2.4 (0.5–12.6)
General disorders and administration site conditions‡	5	(10)	14	(30)	0.3 (0.1–0.8)	0.6 (0.2–1.7)	5	(10)	12	(26)	0.4 (0.1–1.1)	1.0 (0.3–3.4)
Infections and infestations††	4	(8)	3	(6)	1.3 (0.3–5.9)	1.3 (0.3–5.9)	5	(10)	4	(9)	1.2 (0.3–4.6)	1.5 (0.2–8.9)
Injury, poisoning, and procedural complications	4	(8)	3	(6)	1.3 (0.3–5.8)	1.9 (0.4–10.6)	3	(6)	3	(6)	1.0 (0.2–4.8)	1.9 (0.2–21.4)
Eye disorders	3	(6)	2	(4)	1.4 (0.2–8.5)	‡‡	3	(6)	3	(6)	1.0 (0.2–4.8)	‡‡
Other System Organ Classes (<5% in each group)	4	(9)	4	(8)	1.0 (0.2–3.8)	1.3 (0.3–5.7)	7	(14)	5	(11)	1.3 (0.4–4.2)	1.9 (0.4–10.6)

Indicated below, events recorded in the *T. suis* group (by subjects, n = x; doctors/nurses, n = xx) and placebo group (by subjects, n = y; doctors/nurses, n = yy) including multiple types in a subject:

*Eczema (x = 3, xx = 3, y = 1), skin irritation (x = 3, xx = 3, yy = 1), impetigo (x = 1, xx = 1), rash papular (x = 1, xx = 1), rash (x = 1), sun eczema (x = 1, xx = 1), urticaria (x = 1, xx = 1, y = 1, yy = 1), acne (yy = 1).

† Arthralgia (x = 1, xx = 2), myalgia (x = 1, xx = 1, yy = 1), pain in extremity (x = 1, xx = 1), back pain (x = 2, xx = 3, yy = 3), rib fracture (x = 1, xx = 1), wrist fracture (x = 1, xx = 1), musculoskeletal discomfort (x = 1, y = 1, yy = 1), abscess limb (y = 1).

‡ Discomfort (x = 1, xx = 1, y = 1, yy = 1), fatigue (x = 2, xx = 2, y = 7, yy = 5), feeling of body temperature change (x = 2, xx = 2, y = 7, yy = 7), hunger/stomach acid (y = 1, yy = 1), application site reaction (y = 1, yy = 1, unrelated to *T. suis* treatment), alcoholic hangover (y = 1), skin tenderness (yy = 1).

†† Enterobiasis (possible pinworm, xx = 1), herpes zoster (xx = 1), skin infection (yy = 1), ear infection (y = 0), influenza (x = 3, xx = 2, y = 1, yy = 1), chlamydial infection (xx = 1), pneumonia (y = 1,yy = 1), pneumonia viral (yy = 1), varicella (x = 1, xx = 1), and sweating fever (x = 1, xx = 1).

‡‡ RR could not be calculated because of zero cases in ether or both treatment groups.

**Table 3:** Rate ratio (RR) of gastrointestinal adverse events according to maximum severity and type by days and treatment group in a randomized double-blind clinical trial of *T. suis* and placebo, Denmark, 2008.

	Before day 63					On or after day 63					Entire trial				
	*T. suis*		Placebo			*T. suis*		Placebo			*T. suis*		Placebo		
	N = 49		N = 47			N = 49		N = 47			N = 49		N = 47		
	N	(%)	n	(%)	RR (95% CI)	n	(%)	n	(%)	RR (95% CI)	n	(%)	n	(%)	RR (95% CI)
**Total**	38	(78)	30	(64)	1.3 (0.8–2.1)	36	(73)	27	(57)	1.5 (0.4–5.2)	43	(88)	34	(72)	1.3 (0.8–2.1)
Any mild	37	(76)	28	(60)	1.4 (0.9–2.3)	32	(65)	27	(57)	0.5 (0.1–1.9)	40	(82)	34	(72)	1.5 (0.9–2.4)
Any moderate	28	(57)	19	(40)	1.7 (1.0–3.1)	21	(43)	14	(30)	2.1 (0.5–8.0)	34	(69)	22	(47)	2.0 (1.2–3.5)
Any severe	14	(29)	4	(9)	4.0 (1.4–12.1)	8	(16)	4	(9)	1.7 (0.4–6.9)	19	(39)	7	(15)	3.2 (1.3–8.1)
**Moderate to severe**	31	(63)	20	(43)	1.9 (1.1–3.3)	22	(45)	14	(30)	2.0 (0.5–8.0)	37	(76)**	23	(49)**	1.9 (1.1–3.3)
Flatulence	20	(41)	9	(19)	2.5 (1.2–5.4)	10	(13)	6	(13)	1.0 (0.1–15.2)	21	(43)	10	(21)	3.4 (1.4–8.1)
Diarrhea	19	(39)	9	(19)	2.5 (1.1–5.4)	15	(31)	7	(15)	2.0 (0.6–6.7)	27	(55)	13	(28)	2.8 (1.4–5.6)
Abdominal pain	14	(29)	0	(0)	-	7	(14)	2	(4)	1.0 (0.1–6.9)	16	(33)	2	(4)	19.2 (4.3–85.1)
Pruritus ani	5	(10)	7	(15)	0.7 (0.2–2.1)	2	(4)	5	(11)	1.0 (0.1–15.5)	6	(12)	8	(17)	1.0 (0.3–3.6)
Other*	5	(10)	5	(11)	1.0 (0.3–3.2)	6	(12)	1	(2)	3.0 (0.3–28.2)	8	(16)	6	(13)	2.1 (0.7–6.2)
**Diarrhea, abdominal pain, and/or flatulence**															
Total	38	(78)	26	(55)	1.7 (1.1–2.8)	33	(67)	21	(45)	0.6 (0.1–2.5)	41	(84)	31	(66)	1.6 (0.9–2.7)
Any mild	36	(73)	25	(53)	1.7 (1.0–2.7)	28	(57)	21	(45)	0.2 (0.0–1.6)	37	(76)	30	(64)	1.8 (1.1–3.2)
Any moderate	26	(53)	11	(23)	2.9 (1.4–5.8)	18	(37)	10	(21)	1.0 (0.3–3.9)	30	(61)	15	(32)	2.9 (1.5–5.7)
Any severe	14	(27)	3	(6)	5.1 (1.5–17.4)	6	(12)	2	(4)	4.1 (0.5–35.7)	17	(35)	4	(9)	6.5 (2.1–20.1)
Moderate to severe	29	(59)	12	(26)	3.1 (1.6–6.0)	19	(38)	10	(21)	1.0 (0.3–3.8)	33	(67)	16	(34)	2.8 (1.4–5.4)

*Include events occurring in less than 5% of subjects on *T. suis* (n = x), and placebo (n = y): Nausea (x = 3, y = 1), stomach discomfort, (x = 3, y = 2), constipation (x = 1), haemoroids (x = 1), lip dry (y = 1), tooth ache (x = 1, y = 2), oesophagal discomfort (y = 1), oesophagal pain (x = 3, y = 2), vomiting (y = 1), dysphagia (x = 1), gastroenteritis viral (x = 1).

**Among those who believed they received *T. suis* eggs (vs. the remaining subjects), the corresponding percentage was 83% (vs. 71%) in the *T. suis* group and 53% (vs. 47%) in the placebo group. If the allocation was disregarded, the percentage was 70% among those who believed they received *T. suis* eggs and 59% among the remaining subjects.

The daily prevalence of gastrointestinal symptoms in the *T. suis* group peaked once, notably 30 to 50 days after 1^st^ treatment, and then declined before day 63 (3^rd^ treatment) (Figure 1). Until day 63, the *T. suis* group demonstrated significantly increased rates of episodes with moderate to severe flatulence (RR 2.5; 95% CI 1.2–5.4), diarrhea (RR 2.5; 95% CI 1.1–5.4), and upper abdominal pain (placebo, none; *T. suis*, 29%; entire trial, RR 19.2; 95% CI 4.3–85.1) when compared with placebo, and this was not the case after day 63 (overall RR 1.0; 95% CI 0.3–3.8) (Table 3). The median duration of episodes (consecutive days) with the moderate to severe gastrointestinal symptoms was 2.0 days over the entire trial (vs. 1.3 days for placebo, P = 0.0006), and 2.5 days if onset was before day 63 (vs. 1.0 day for placebo, P = 0.002) (subjects with repeated episodes were represented by the mean duration of these episodes). The combined duration of episodes with onset before the prevalence peak (day 42) was ≤14 days in 80% of affected subjects in the *T. suis* group. The median duration of severe episodes over the entire trial was 1.4 days (vs. 1.0 day for placebo, P = 0.12).

**Figure 2:**
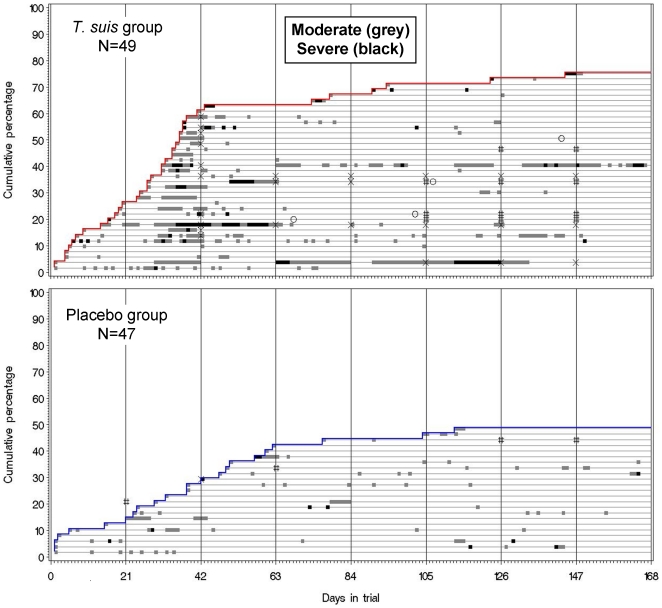
Incidence of gastrointestinal symptoms after three-weekly ingestions of infective *T. suis* eggs by 49 subjects, and placebo by 47 subjects, in a randomized placebo-controlled double-blinded clinical trial, Denmark 2008. Vertical lines represent days of clinic visits (three-weekly) when subjects were to ingest 2500 live *T. suis* eggs or placebo except on visit day 168 (total 8 doses). Each horizontal thin line represents a subject. Episodes of each subject are indicated by grey (moderate) and black (severe) horizontal thick lines. “X”, the subject ingested no eggs/placebo due to gastrointestinal symptoms; “#”,the subject ingested no eggs/placebo due to reasons unrelated to the intervention; “O”, the subject stopped recording diary of symptoms before end of trial.

Figure 2 depicts the data underlying the above findings of a higher rate, severity, and duration of episodes with moderate to severe gastrointestinal symptoms in the *T. suis* group, and demonstrates that the incidence (i.e. cumulative percentage) of affected subjects increased from the first few days and until day 42. Accordingly, after 21 days the cumulated percentage of subjects who had their first episode of the symptoms (i.e. regardless of later episodes, if any) was 27% vs. 15% for placebo (Figure 2). After 42 days, a total of 63% had the first episode of symptoms vs. 29% for placebo. At the end of the study, the percentages were 76% vs. 49%, respectively (Figure 2). The corresponding cumulative percentages for first episode of moderate to severe diarrhea were 12% vs. 6% after 21 days, 29% vs. 11% after 42 days, and 55% vs. 28% at end of study.

### Treatment compliance

**Figure 3:**
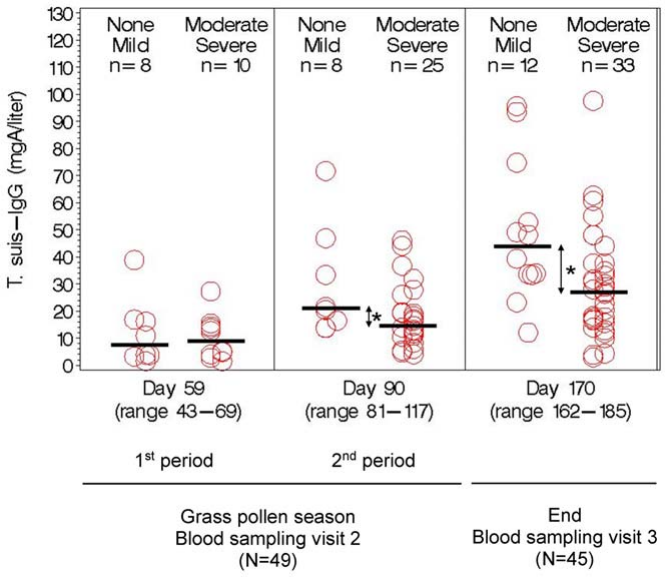
Level of serum IgG against adult *T. suis* excretory/secretory antigen over time according to severity of gastrointestinal symptoms after three-weekly ingestion of infective *T. suis* eggs by 49 subjects in a randomized placebo-controlled double-blind clinical trial in grass-pollen allergic adults, Denmark 2008. Within subgroups of severity, data circles are positioned by severity (None, left; Mild, right), (Moderate, left; Severe, right). Blood was drawn times 3 (blood visit 1 at start (not shown, no IgG response)), blood visit 2 during grasspollen season (1^st^ period and 2^nd^ period), and blood visit 3 at end of study), and diary was kept day 0–168, except in 4 subjects indicated by circles in figure A: 2 subjects (severe) dropped out (moved abroad, no time) between blood visit 2 (day ∼90) and 3, however recorded diary until day ∼90; 2 other subjects (moderate, severe) recorded diary until blood sampling visit 2 (day ∼59) or longer (>day 90) although they did not attend blood visit 3. * P<0.05.

Figure 2 also presents treatment compliance for subjects who reported moderate to severe gastrointestinal symptoms, marked as X (treatment-related) and # (not treatment-related). No treatments were discontinued because of mild gastrointestinal symptoms. A total of 12 subjects on *T. suis* (24%) started discontinuing treatment because of moderate to severe gastrointestinal symptoms that we judged to be treatment-related (vs 2% (n = 1) for placebo, P<0.001). However, 8 of the subjects (16%) continued again after pausing one treatment only, and 4 of the subjects (8%) discontinued treatment the rest of the study (but continued symptom diary, 2 a few months further, 2 until end) (see figure 2). No other subjects in the study had treatment-related discontinuation of treatments. Because of reasons unrelated to treatment, 6 subjects on *T. suis* (4 had moderate to severe symptoms, thus shown in figure 2) and 3 subjects on placebo (2 shown in figure 2) discontinued one or more treatments (12% vs. 6%, P = 0.32), however, 2 of the 6 subjects on *T. suis* had previously discontinued one treatment due to treatment-related gastrointestinal symptoms (both shown in figure 2). The exact distribution of the total number of treatments received was as follows (ds, doses): *T. suis* group (N = 49): 8 ds, n = 32 (65%); 7 ds, n = 9 (18%); 6 ds, n = 1 (2%); 5 ds, n = 3 (6%); 4 ds, n = 1 (2%); 3 ds, n = 1 (2%); 2 ds, n = 2 (4%). Placebo group (N = 47): 8 ds, n = 41 (87%); 7 ds, n = 4 (9%); 6 ds, n = 2 (4%).

### Potential risk factors for gastrointestinal symptoms

Table 4 shows potential risk factors for gastrointestinal symptoms before day 63. Overall, the rate of first moderate to severe gastrointestinal symptoms in the *T. suis* group vs. placebo was not modified by the bulk filled on the vials for the 1^st^ and 2^nd^ treatment (P = 0.31); sex (P = 0.23); age (P = 0.72); body mass index (P = 0.52); duration of allergic rhinitis (P = 0.65); allergic co-morbidities including asthma, birch-pollen allergy, atopic dermatitis, food allergy, or symptomatic cross-reactions to allergens (P = 0.29); allergy of mother and/or father (P = 0.76); total IgE level (P = 0.77); any gastrointestinal morbidity in the three weeks before 1^st^ treatment (P = 0.15); pets ever in household (P = 0.06); and current or ever smoking (yes vs. no, P = 0.92 and 0.41). The results presented in this paragraph were similar for entire trial and for diarrhea (data not shown).

**Table 4:** Rate ratio of first moderate to severe gastrointestinal symptoms (before day 63) within strata of characteristics in a randomized placebo-controlled double-blind clinical trial of *T. suis* for grass-pollen allergy, Denmark, 2008.

	*T. suis*		Placebo		RR (95% CI)	P-value for RR- modification by strata
Overall	31/49	(63%)	20/47	(43%)	2.1 (1.2–3.5)	
***T. suis*** ** egg or placebo bulk filled onto vials intaken at** *						
**visit 1; visit 2**						
Bulk 1; bulk 2 or 3	11/13	(85)	5/12	(42)	4.2 (1.4–12.3)	
Bulk 2; bulk 3	9/17	(53)	7/18	(39)	1.7 (0.6–4.6)	P = 0.31
Bulk 3; bulk 3 or 4	11/19	(58)	8/17	(47)	1.3 (0.5–3.1)	
**Sex**						
Male	30/47	(64)	18/45	(40)	2.1 (1.1–3.7)	P = 0.23
Female	½	(50)	2/2	(100)	0.6 (0.1–7.0)	
**Age**						
20–32 years	17/25	(68)	6/15	(40)	2.0 (0.8–5.1)	P = 0.72
33–63 years	14/24	(58)	14/32	(44)	1.7 (0.8–3.7)	
**BMI (kg/height^2^)** ††						
Normal	17/27	(63)	9/17	(53)	1.6 (0.7–3.6)	P = 0.52
Overweight/obese	14/22	(64)	11/29	(38)	2.1 (0.9–4.6)	
**Duration of allergic rhinitis** (years)						
3–17 years	17/25	(68)	7/17	(41)	2.2 (0.9–5.3)	P = 0.65
18–53 years	14/24	(58)	13/30	(43)	1.7 (0.8–3.6)	
**Allergic comorbidity** †						
No	4/9	(44)	4/8	(50)	0.9 (0.2–3.8)	P = 0.29
Yes	27/40	(68)	16/39	(41)	2.2 (1.2–4.1)	
**Allergy of mother and/or father**						
No	15/23	(65)	11/27	(41)	2.2 (1.0–4.7)	P = 0.76
Yes	16/26	(62)	9/20	(45)	1.7 (0.7–3.8)	
**Total IgE (kU/l) at baseline**						
Low (<100)	13/19	(68)	10/19	(53)	1.8 (0.8–4.1)	P = 0.77
High (100–1200)	18/30	(60)	10/28	(36)	2.1 (1.0–4.5)	
**Gastrointestinal morbidity** ‡						
No	16/27	(59)	7/26	(27)	3.02 (1.2–7.4)	P = 0.15
Yes, recent	15/22	(68)	13/21	(62)	1.23 (0.6–2.7)	
**Pets ever in household**						
None	15/22	(68)	12/17	(71)	1.1 (0.5–2.3)	P = 0.06
Any	16/27	(59)	8/30	(27)	3.0 (1.3–7.1)	
**Smoking**						
No	20/30	(67)	10/27	(37)	2.3 (1.1–5.0)	P = 0.41
Ever	11/19	(58)	10/20	(50)	1.5 (0.6–3.4)	
No	27/43	(63)	17/42	(41)	1.9 (1.1–3.6)	P = 0.92
Current	4/6	(67)	3/5	(60)	1.8 (0.4–8.0)	

*The numbers of embryonated eggs, counted by quality-controlled microscopy, in randomly selected vials from bulk one to five were 2310, 2010, 2355, 2400, and 2400, respectively. Due to small numbers some bulk groups were joined.

† Included birch-pollen induced allergic rhinitis (defined as subjects having birch-IgE≥0.7 kUA/l, birch-SPT≥3 mm, and reporting significant symptoms to birch-pollen in ≥1 of the last 4 years), food allergy, symptomatic cross-reactions to allergens, a diagnosis of asthma, or a diagnosis of atopic eczema.

†† Overweight/obese defined as BMI≥25. One subject was missing information on height.

‡ Subjects with any diarrhea, flatulence, pruritis ani, or other gastrointestinal disorder 3 weeks before trial (n = 43 of 96).

### Gastrointestinal symptoms and blood analyses

To investigate whether self-reported gastrointestinal symptoms were related to objective measurements, results of blood analyses were stratified by the two significantly different subgroups of moderate/severe and none/mild gastrointestinal symptoms (Table 5 and Figure 3).

**Table 5:** Median value of blood parameters over time according to severity of gastrointestinal symptoms after ingestion of whipworm *T. suis* eggs (N = 49) in a randomized double-blind clinical trial of *T. suis* for grass-pollen allergy, Denmark, 2008.

		Grass pollen season‡						
	Start	1^st^ period		2^nd^ period		End		
		None	Moderate	None	Moderate	None	Moderate	P-value (time, severity)†
		Mild	Severe	Mild	Severe	Mild	Severe	
	N = 49	n = 8	n = 10	n = 8	n = 25	n = 12	n = 33	
(different from placebo‡‡)								
*T. suis*-IgG (mgA/l)	1.90	7.4	8.9	21.0*	14.6	43.8*	27.1	0.02
*T. suis*-IgE (kUA/l)	<0.01	0.04	0.03	0.28	0.23	2.95*	1.35	0.07
*T. suis*-IgE (kUA/l)	<0.01	0.04	0.03	0.12	0.18	0.29	0.35	0.70
*T. suis*-IgA (mgA/l)	2.85	2.76	3.01	3.70	3.84	3.47	3.03	0.87
Eosinophil count (10^9^/l)	0.20	0.68	0.61	0.57	0.48	0.34	0.45	0.12
(not different from placebo‡‡)								
Total IgE (kU/l)	70.9	62.3	70.4	100.0	139.1	96.0	123.5	0.86
Non-specified IgE (kU/l)††	53.1	50.4	42.5	87.3	109.7	80.8	78.1	0.82
Total histamine (ng/ml)	100	146	101	109	139	77	109	0.08
Basophil count (10^9^/l)	0.03	0.06*	0.03	0.04	0.04	0.04	0.05	0.61
Haemoglobulin (mmol/l)	9.2	9.3	9.3	9.5	9.3	9.2	9.2	0.55
Leucocyt count (10^9^/l)	6.00	7.70*	6.00	6.80	5.95	6.55	6.15	0.78
Lymphocyt count (10^9^/l)	1.75	2.34	1.79	1.79	1.75	1.76	1.83	0.09
B-erythrocytes, MCV (10^15^/l)	90.0	89.5	88.5	92.5	90.5	89.0	88.5	0.36
Monocyt count (10^9^/l)	0.43	0.56	0.40	0.45	0.40	0.43	0.41	0.60
Neutrophil count (10^9^/l)	3.04	3.71	2.97	3.53	2.88	3.56	3.12	0.41
Erytrocyt count (10^12^/l)	12.9	13.2	13.0	13.3	13.0	13.1	12.8	0.42
Thrombocyt count (10^9^/l)	252	299	303	262	253	255	245	0.32

*P<0.05; mgA/l, milligrams *T. suis*-specific antibodies per liter serum; kUA/l, kilo units antibodies per liter serum.

† P-value for a test for homogeneity between trends over time (in *mean* values) in each severity group. Intersubject correlation was taken into account.

‡ Each subject had one blood sample drawn during the grass pollen season (May 28 to July 27), and all sampling days were then categorized into 1^st^ period (visit 3 of 9, n = 8; visit 4 of 9, n = 10) and 2^nd^ period (visit 5 of 9, n = 27; visit 6 of 9, n = 6) to obtain meaningful results by severity subgroups. The mean day of 1^st^ period was day 59 (range 43–69), 2^nd^ period day 90 (range 81–117), and end day 170 (162–185).

†† Calculated for each subjects as total IgE minus the total sum of IgE against *T. suis*, grass- and birch-allergen.

‡‡ The result of planned analyses (data not shown) executed on the date (March 4, 2009) the study was unblinded.

The placebo group is not shown.

Figure 3 demonstrates serum level of *T. suis*-IgG over time in the *T. suis* group according to maximum severity of gastrointestinal symptoms before consecutive blood sampling dates (Data is not shown for the placebo group, because after first treatment any *T. suis*-response were significantly higher in the *T. suis* group than the placebo group) [12]. Overall, the *T. suis*-IgG level in the *T. suis* group (Figure 3, and Table 5) increased over time (P_trend_<0.0001). However, subjects who had had mild or no symptoms exhibited a significantly larger increase in levels (P_difference in trend_ = 0.02) and higher final levels than subjects who had moderate or severe symptoms (P_day 59(1.period)_ = 0.71, 7.4 vs. 8.9 mgA/liter; P_day 90 (2. period)_ = 0.79, 21.0 vs. 14.6 mgA/liter; P_day 170(End)_ = 0.0001, 43.8 vs. 27.1 mgA/liter). For *T. suis*-IgG4, overall levels also increased over time (P_trend_<0.0001) and again subjects with mild or no symptoms had significantly higher final levels (P_difference in trend_ = 0.07, P_day 59(1.period)_ = 0.37, P_day 90(2.period)_ = 0.32, P_day 170(End)_ = 0.002). For *T. suis*-IgA, *T. suis*-IgE and eosinophil counts, overall levels increased moderately and significantly over time, as reported previously [12], and the two symptom subgroups were not significantly different (Table 5). All results presented in this paragraph were not materially different when restricting analyses to subjects who received all 8 treatments with *T. suis* ova (data not shown).

Using the data points in figure 2 and a cut-off value of 5.1 mgA/L (95% CI 3.9–6.2), determined previously in 15 non-atopics [12], to identify *T. suis*-IgG seroconverted subjects, then seroprevalences over four, not only three, time points was calculated for the *T. suis* group: 0% (0/0) at day 1, 50% (9/18) at day 59, 91% (30/33) at day 90, and 93% (42/45) at day 170. The seroprevalences are for the *T. suis* group only, because no significant *T. suis*-specific response was seen in the placebo group (2 false-negative at baseline, vs. 1 false-positive in the *T. suis* group at baseline; sensitivity 96% and specificity 98%).

Finally, total IgE, non-specified IgE (i.e. total IgE minus *T. suis*-, grass-, and birch-IgE in each subject) and total blood histamine (a proxy measure for blood basophils) and other hematology than eosinophil counts (e.g. lymphocyte counts) were not different from placebo at any time point (data not shown) and the *T. suis* group was therefore not studied further for these parameters, except as shown in table 5.

## Discussion

The present study demonstrated that controlled infection of humans with the pig parasite *T. suis* caused a three to 19-fold increased rate of episodes with flatulence, diarrhea, and abdominal pain. The first ingestion of a dose of 2500 *T. suis* eggs, and/or ingestion of the next dose 21 days later, caused these transitory side effects to appear during the first 1½ month (42 days), because there was no similar effect after later intake of eggs. The repeated infections occurred along with increasing *T. suis*-specific IgG, IgG4, IgA, and IgE levels. However, the *T. suis*-IgG-response developed slower in subjects who had had gastrointestinal reactions – an observation we speculate could be due to expulsion of *T. suis* larvae during initial exposure in some humans.

Gastrointestinal side effects were also reported in a recent clinical phase I trial that evaluated the safety of *T. suis* treatment in five multiple sclerosis (MS) patients [15]. The authors used comparable egg dose (2500) and interval (14 days) over three months (total 6 doses), and three of the five subjects experienced the onset of mild gastrointestinal symptoms (FDA scale grade 1, no interference with activities of daily living such as school or work, 2–3 loose stools per day) at about 30 days after the first dose of *T. suis* ova. Spontaneous resolution of these symptoms occurred after 6, 1, and 4 days in each subject. These results are compatible with our observations, although based on smaller numbers and information collected not daily but monthly at clinical visits. In contrast to both reports, no side effects were observed in three earlier IBD studies which used similar egg dose (2500) and interval (21 or 14 days) and improved gastrointestinal symptoms in Crohn's disease and ulcerative colitis patients [3]–[5], [22]. A significant change in disease activity, e.g. patient-reported stool frequency, was reported to start as early as 1½–3 months after 1^st^ dose [4], which is not as early as diarrhea due to *T. suis* in our allergic rhinitis patients. Possible explanations for the lack of gastrointestinal side effects could be that the already increased stool frequency in IBD patients made it difficult to detect temporary *T. suis*-induced changes in stool frequency, or that the IBD patients received immune-suppressive drugs which then concealed early intestinal side effects. However, although similar information was collected in the IBD studies and the present study (stool frequency, diarrhea), the IBD study with the most detailed temporal data presented results using a 2-weekly disease activity index (the Simple Index) with multiple components (stool frequency, urgency of defecation, blood in stool, general well-being, and extracolonic features), and is therefore not directly comparable.

Despite the lack of side effects in clinical trials of *T. suis* prior to the present (i.e. the IBD studies), we suspected there would be some intestinal reactions during infestations with whipworms, and therefore implemented standard questions to increase detection. We did this as a supplement to the spontaneous reporting of adverse events, and anticipated that it might increase awareness of adverse events, although not differently between treatment groups. Compatible with this there was a similar rate of *mild* gastrointestinal symptoms by treatment group, but in the *T. suis* group we detected a significantly higher rate and duration of *more* severe (i.e. moderate to severe) gastrointestinal symptoms, which we therefore interpreted as side effects and *not* increased awareness. Importantly, rates of side-effects were also increased for rates of first episode of symptoms, i.e. thus excluding bias from awareness due to previous symptoms from *T. suis*. In addition, we evaluated whether subjects guessed their treatment allocation despite the blinding, and found no statistical evidence to support this.

We did not obtain a definitive diagnosis of flatulence, diarrhea, and abdominal pain, for example by using systematic examinations or sampling of stools. This was simply because the side-effects were unexpected and ethical permission for further systematic examination of subjects could not have been obtained in due time. However, as illustrated in figure 2, the symptoms were transitory, and therefore in fact difficult to observe with the three-week visit interval. For example, in three cases we recorded our results of abdominal palpations at their visit at the clinic and found nothing abnormal, despite that the subjects had had weeks with diarrhea and abdominal pain. Overall however, we inquired systematically about, e.g., diarrhea in a follow-up questionnaire one year after the study, and found that stools had never been bloody, always watery (75% responded in each treatment group, data not shown).

We also found further support that the side-effects were clinically significant, when we considered the repeated evaluations at visits by doctor, nurse and subject, of any unusual moderate to severe gastrointestinal symptoms since last visit. Based on these blinded evaluations, it was decided in 13 cases to pause next treatment (see figure 2), and it turned out after the unblinding of the allocation at the end of the study that 12 of these cases had received *T. suis* eggs [12]. Only one similar decision was made for a placebo subject, but merely because of one episode (see figure 2, Placebo) which coincidently was around day 42 and in connection with asthma symptoms. Taken together, this strongly argues in favor of a clinically significant side effect in the 12 subjects in the *T. suis* group (i.e. 24%). Arguably, the significance of the gastrointestinal symptoms reported by the further 25 subjects (76%−24% = 52%, see figure 2), who did not likewise pause treatments, may be questioned. However, figure 2 shows that most of these subjects had a similar pattern of symptoms. Furthermore, the reason they did not pause treatment was not necessarily that they did not have significant gastrointestinal symptoms, but simply that we had not advised that they pause treatment. For example, we advised only some subjects to pause 2^nd^ treatment, because early on we were reassured by the observation that subjects 1–2 months ahead of others in treatment schedule rarely had gastrointestinal symptoms after 3–5 treatments.

We investigated in several ways why only a proportion of subjects had gastrointestinal side effects after ingestion of *T. suis* eggs. Reassuringly, the quality of the egg manufacture did not explain this. For example, minor dose variation was allowed in the manufacture, but the variation (2010–2400 eggs) was not important to the rate of first episodes with side effects. It is therefore interesting that factors like age, gastrointestinal morbidities, smoking, and allergies did not provide an explanation. We did not have statistical power to test a role of gender, although we observed that one of the four included women did report diarrhea between 2^nd^ and 3^rd^ treatment, and received *T. suis* eggs (1 of 2 women (50%) vs. 0 of 2 women on placebo, 0%). In the cited recent clinical trial where five multiple sclerosis patients were treated with *T. suis* eggs, three of four women (75%) reported gastrointestinal symptoms, while the male reported no symptoms [15].

In explorative analyses we investigated whether the self-reported gastrointestinal side effects were related to objective measurements in blood, because such relation could strengthen the validity of self-reports. In addition, the analysis could help understand risk factors for gastrointestinal reactions and whether *T. suis* is expelled by such reactions. In support of the validity of self-reports, we observed that the *T. suis*-IgG response developed faster in subjects without than with gastrointestinal side effects.

With regards to expulsion, blood parameters that are recognized effectors in clearance of gastrointestinal helminths (IgE, IgA, eosinophils) [23]–[27], did not differ between subjects with and without gastrointestinal sides effects, despite an overall response to *T. suis*. However, such differences could have been difficult to detect because subjects in general had a low *T. suis*-IgE response, local mucosal IgA and eosinophils were not measured, and eosinophil responses are non-specific and were a result of both *T. suis* and allergen exposure (allergic disease). In addition, IgA and IgE have a shorter serum half life than IgG (<6 vs. 21 days) which may partly explain the observed concentrations were low. We did not sample stools and therefore could not measure e.g. copro-antigen, eggs, larvae, or worms in feces. However, *T. suis* rarely mature and produce eggs in aberrant hosts [3], [16], and it is therefore not certain that these measures would document expulsion if there was one. We speculated that the slower *T. suis*-IgG response in subjects with gastrointestinal reactions is suggestive of expulsion of *T. suis*, because in single-inoculated pigs serum IgG and IgM antibody responses are reduced within weeks after documented expulsion [28], and diarrhoea can be observed in pens when expulsion starts in week 7 (day 51) post-inoculation (authors' unpublished results). Furthermore, in pigs and mice, one suggested mechanism of expulsion of *Trichuris* species is through the IL-4/IL-13 system [29], [30], with a tightly controlled IL-13 induced dramatic elevation in epithelial cell proliferation and crypt cell hyperplasia that could detach worms from the intestinal wall [28], [31]. It is conceivable that such histological changes would cause gastrointestinal symptoms. The reason why the observed gastrointestinal symptoms, and thus perhaps expulsion, accompanied initial and not later ingestions, is unclear. At least, expulsion during later ingestions, i.e. corresponding to immunity to *T. suis*, seems unlikely because the ingestions induced further antibody responses even in affected subjects, and in the previous IBD trials they maintained efficacy [4], [5], [22]. Possible explanations, however, may involve different immunological memory (*T. suis*-specific T cells and antibodies), helminth-induced immune-suppression (regulatory T-cells, IL-10, TGFβ), and/or histological changes in the intestinal wall [32]. The reason why symptoms occurred in a proportion of subjects only is also unclear. If the symptoms were associated with expulsion of *T. suis*, a genetic component could be involved because resistance to *T. suis* infections has been reported to be different between piglets of different parental background [33].

Overall, the *T. suis*-IgG response and other studied factors did not provide a method to identify subjects at risk of gastrointestinal side effects, suggesting that it will continue to be a reasonable precaution to ingest less than 2×2500 *T. suis* eggs during the first 42 days when such treatment is tested or prescribed. During longer treatment (63 to 168 days) with this dose the risk of side effects is statistically insignificant, also after treatment is stopped (1-year follow-up data not shown).

In conclusion, during the first two months, ingestions of 2500 *T. suis* eggs caused frequent episodes with moderate to severe gastrointestinal reactions lasting up to two weeks. The response reflects the initial exposure to the parasite and the associated immune response. Data from the last four months of the study suggest that ingestions over longer time mainly provoke a subclinical stimulation.

## Supporting Information

Protocol S1 **Trial Protocol.**
(PDF)

Checklist S1 **CONSORT Checklist.**
(DOC)
