# Supplementary material for: Symptoms after Ingestion of Pig Whipworm Trichuris suis Eggs in a Randomized Placebo-Controlled Double-Blind Clinical Trial
Source: PLoS One. 2011 Aug 2;6(8):e22346. doi: 10.1371/journal.pone.0022346 (PMC3149054; doi:10.1371/journal.pone.0022346)
Supplement: Protocol S1 — Trial Protocol. (PDF) [file pone.0022346.s001.pdf]

STATENS
SERUM
INSTITUT

*prevention and control
of infectious diseases
and congenital disorders*

5 Artillerivej
2300 Copenhagen S
Denmark

Tel: +45 3268 3268
Fax: +45 3268 3868
serum@ssi.dk
www.ssi.dk

PROTOCOL

(Version 3: Amendment protocol)

February 19, 2008

Oral treatment of allergy with a non-pathogenic microorganism:
A randomised clinical trial

SSI Trial code: TSO-01
Eudract number: 2007-006099-12
DKMA: j.nr. 2612-3616
Ethical Committee: j.nr. H-KF-2006-4100
Data Protection Agency: j.nr. 2006-54-2215
UMIN Clinical Trial Registry: Reg. no. R000001298, Trial ID UMIN000001070

Peter Bager, Study Director
Department of Epidemiology Research
Statens Serum Institut
Denmark

Confidentiality

This document contains confidential information. The contents may not be used, divulged or published without prior written consent of Statens Serum Institut. This information cannot be used for any other purpose than the conduct and evaluation of the clinical investigation by the Investigator(s), the regulatory authorities and members of the ethics committees.

Table of Contents

1 Synopsis 4
2 List of abbreviations 6
3 Signature Page 8
4 Addresses 9
5 Introduction 13
6 Trial objectives 15
6.1 Primary objective 15
6.2 Secondary objective 15
6.3 Primary variables 15
6.4 Secondary variables 16
7 Investigational plan 17
7.1 Overall design 17
7.2 Study population 20
7.3 Recruitment of volunteers 20
7.4 Inclusion criteria 20
7.5 Exclusion criteria 20
7.6 Withdrawal and predetermined reasons for discontinuation 21
8 Investigational products 21
8.1 Treatments administered 21
8.1.1 Dosages and administration 22
8.1.2 Packing and labelling 23
8.1.3 Storage information 27
8.1.4 Transport of trial products 27
8.1.5 Randomization procedure 27
8.1.6 Compliance and drug accountability 29
8.1.7 Blinding 31
8.1.8 Precautions 31
8.1.9 Concomitant medication 32
9 Investigational events 33
9.1.1 Details of visits 35
9.1.2 Efficacy assessments 38
9.1.3 Safety assessments 39
9.1.4 Immunological assessments 39
9.1.5 Handling of blood samples 39
9.1.6 Laboratory assays 41
9.1.7 Follow-up 41
9.1.8 Time schedule and end of trial 42
10 Ethical aspects 42
10.1 Payment of volunteers and investigational institution 44
10.2 Budget 44
11 Adverse events 45
11.1 Definitions and terms 45

11.2 Standard reporting of adverse events ... 46
11.3 Expedited reporting of serious adverse events ... 47
12 Data management and statistical analysis ... 48
12.1 General considerations ... 48
12.2 Data management ... 48
12.3 Clean file procedures ... 51
12.4 Statistical analysis ... 51
12.4.1 Primary endpoints ... 52
12.4.2 Secondary endpoints ... 54
13 Good Clinical Practice considerations ... 58
13.1 Declaration of Helsinki ... 58
13.2 Subject information and informed consent ... 58
13.3 Ethics committee ... 58
13.4 Health authority submission and approval ... 59
13.5 Subject data protection ... 59
13.6 Investigators responsibilities ... 60
13.7 CV and log of staff ... 60
13.8 Training ... 61
13.9 Monitoring ... 61
13.10 Audit and Inspection ... 61
13.11 Definition of source data ... 61
13.12 Archiving ... 61
14 Agreement and financial settlement ... 62
15 Insurance ... 62
16 Confidentiality and disclosure ... 63
17 Changes to the protocol ... 63
18 References ... 65
Appendix 2: Declaration of Helsinki ... 68
Appendix 3: Consent form ... 71
Appendix 4: Participant information ... 72
Appendix 5: Investigator's statement ... 78
Appendix 6: Indemnity Statement ... 79
Appendix 7: Scores for relief medication ... 80
Appendix 8: Guidelines for verbal information of participants ... 81
Appendix 9: Subject Screeening Log ... 83
Appendix 10: Subject Identification Code List ... 84
Appendix 11: Subject Enrolment Log ... 85
Appendix 12: Product accountability document ... 86
Appendix 13: Check list for Investigator's and Sponsor's File ... 87

1 Synopsis

**Title**	Oral treatment of allergy with a non-pathogenic microorganism: A randomised clinical trial
**Trial code**	TSO-01
**Eudract number**	2007-006099-12
**Trial phase**	Phase II trial
**Primary objective**	To assess the efficacy of transient *Trichuris suis* infestation of the gut on self-reported symptoms of allergic rhinitis and number of well-days among adults with allergic rhinitis.
**Secondary objective**	To assess the efficacy and safety of transient *Trichuris suis* infestation of the gut on allergic rhinitis and asthma assessed through self-reported disease activity, clinical skin and airway activity, and immunological activity among adults with allergic rhinitis.
**Primary variables**	Daily score for symptoms of allergic rhinitis, and number of well days during the pollen season.
**Secondary variables**	<u>Efficacy variables</u><br>Self-reported disease activity related to allergic rhinitis<br>a. Daily recording of use of medication for allergic rhinitis, calculated as a score during data management.<br>b. Daily score for all symptoms of allergic rhinitis on a 100mm visual analog scale (VAS).<br>c. Assessment at the end of the trial of all symptoms of allergic rhinitis during the trial when compared with symptoms during the previous pollen season.<br>d. Weekly score for quality of life using a validated Rhinitis Quality of Life Score.<br>Clinical skin and airway activity<br>e. Skin test reactivity to inhalant allergens.<br>f. Degree of airway inflammation measured by amount of exhaled nitrogen oxid into a Niox apparatus.<br>Immunological activity<br>g. Reactivity of specific IgE and level of specific IgG/IgG4 to inhalant allergens measured by ImmunoCAP^{TM}. Differential blood count.<br>h. Cytokines (IL-4, IL-5, IL-13, IL-10, TGFβ, IFNγ) measured as *in vitro* production from peripheral blood mononuclear cells in response to stimulation with allergens and *Trichuris suis* antigen. Furthermore, total histamine in serum (Subsample of participants).<br><u>Efficacy and safety variables</u><br>Self-reported disease activity related to asthma and adverse events<br>i. Daily recording of use of medication for asthma, calculated as a score during data management.<br>j. Daily score for symptoms of asthma (safety variable).<br>k. Daily adverse events (safety variable)<br>Clinical airway activity<br>l. Daily lung function measured as forced expiratory volume 1 (FEV1, safety variable) and peak expiratory force (PEF) using a pocket flow-meter.
**Trial design**	A single-centre triple-blinded placebo controlled phase II trial including

	100 adult male and non-fertile female volunteers with allergic rhinitis. The volunteers will be randomized to treatment with 2500 eggs of *Trichuris suis* and placebo every 3rd week for 24 weeks. Pariticipants will be followed up 6 weeks later.<br>Measurements and assessments include schematic diaries of symptoms and medication for allergic rhinitis and asthma, and quality of life (scoring systems). Furthermore, daily lung function, 2 skin prick test for allergens, 3-4 measurements of airway inflammation, and 3 blood samples analysed for reactivity to *T. suis*, allergens, and for immune responses possibly associated with therapeutic activity. The follow-up includes a questionnaire. If an effect is observed we plan to study potential effects during the next pollen season as well.
**Inclusion criteria**	1. Male or non-fertile female, aged $\geq$18 and $\leq$65 years<br>2. Symptoms of grass pollen allergy the last 2 pollen seasons, or more.<br>3. Forced Expiratory Volume 1 >70% of expected.<br>4. Scoring all symptoms of allergic rhinitis >50 mm on a 100 mm VAS (0=not bothersome, 10=worst) during previous pollen season.<br>5. Specific IgE class $\geq$2 to grass ($\geq$0.7 $kU_{allergen}$/l)<br>6. A positive skin prick test ($\geq$3mm) to grass<br>7. Prepared to grant authorized persons access to medical records<br>8. The volunteer is likely to comply with instructions
**Exclusion criteria**	9. Significant asthma<br>10. Use of systemic steroids during the last 2 months<br>11. Immune therapy for grass pollen allergy the last 2 years.<br>12. Planning a travel abroad during the trial period (excluding areas with a similar grass/birch pollen counts when compared with the Copenhagen area)<br>13. Past or current severe diseases (a history of Crohn's disease, ulcerative colitis, multiple sclerosis, active hepatitis B or C, cytomegalovirus, herpes simplex, HIV, other kinds of immune deficiency, and cancer)<br>14. Anti-helminth treatment within the last 2 weeks<br>15. Known or possible hypersensitivity to *Trichuris* species or compounds made of *Trichuris* species<br>16. A past or recent drug abuse<br>17. Participation in other clinical trials<br>18. Employed with the Investigator (Pulmonology- and Allergy Clinic Copenhagen (center of trial)), or the relevant department at the sponsor institution (Department of Epidemiology Research at Statens Serum Institut).
**Trial population**	100 voluntary adults allergic to gras pollen, male and non-fertile female
**Country**	Denmark
**Investigational products**	Active product: An aqueous suspension of living embryonated eggs of *Trichuris Suis* (*Trichuris Suis* Ova = TSO®) stabilised in 0.1 N sulphuric acid (pH 1). The concentration is 167 eggs/ml. *Trichuris Suis* is a parasite with pigs as natural hosts, and with favourable characteristics for human use (non-pathogenic, non-invasive, and can not multiply) Placebo product: An aqueous suspension of stablised =0.1 N sulphuric acic (pH 1).
**Dosage**	2500 TSO® or placebo
**Dosage volume**	15 ml
**Administration**	Oral (sulphuric acid is neutralised by adding sodiumhydrogencarbonate powder)

2 List of abbreviations

AE	Adverse events
AR	Allergic rhinitis
CIOMS	Council for International Organizations of Medical Sciences
CRF	Case Record Form
CPR	The Danish Civil Registration Number
CV	Curriculum vitae
DMI	Danish Metrological Institut [Dansk Metereologisk Institut]
DKMA	Danish Medicine Agency [Lægemiddelstyrelsen]
DPA	Danish Data Protection Agency
EC	Ethics Committee of Copenhagen and Frederiksberg Municipalities
ELISA	Enzyme Linked Immunosorbent Assay
FEV1	Forced Expiratory Volume 1
PEF	Peak Expiratory Force
GCP	Good Clinical Practice
GMP	Good Manufactoring Practice
IB	Investigator's Brochure
IBD	Inflammatory Bowel Disease (e.g. Morbus Crohn's and ulcerative colitis)
ICH	International Committee on Harmonisation
IgG	Immunoglobulin G
IgE	Immunoglobulin E
KPLL	Københavns Praktiserende Lægers Laboratorium
KVL	The Royal Veterinary and Agricultural University [Den Kongelige Veterinære Landbohøjskole]
PACC	The Pulmonology and Allergy Clinic Copenhagen [Lunge- og Allergiklinikken København]
PBMC	Peripheral Blood Mononuclear Cells
SAE	Serious Adverse Event
SSI	Statens Serum Institut
sIg	Specific Immunoglobulin (E, G, G4, or A)
TMF	Trial Master File
VAS	Visual Analog Scale

List of short terms

Clinical skin activity	Skin test reactivity to inhalant allergens
Clinical airway activity	Degree of airway inflammation (NO measurement) OR lung function measured by FEV1 (Piko-1 pocket flowmeter)
Immunological activity	Reactivity of specific antibodies to inhalant allergens (serological analyses) OR cytokine responses (*in vivo*, i.e. in serum; *in vitro*, i.e. by antigen stimulation of leukocytes).<br><br>Differential blood count and total histamine in serum.
Self-reported disease activity	Selfreported: Medication/symptom-score for allergic rhinitis and asthma OR rhinitis quality of life score, OR adverse events

3 Signature Page

Principal Investigator	John Arnved<br>Pulmonology and Allergy Clinic Copenhagen	---<br>*Date and signature*
Investigator	Steen Rønborg<br>Pulmonology and Allergy Clinic Copenhagen	---<br>*Date and signature*
Study Director	Peter Bager<br>Department of Epidemiology Research<br>Statens Serum Institut	---<br>*Date and signature*
Data manager	Jan Wohlfahrt<br>Department of Epidemiology Research<br>Statens Serum Institut	---<br>*Date and signature*
Sponsor	Mads Melbye<br>Department of Epidemiology Research<br>Statens Serum Institut	---<br>*Date and signature*

LUNGE & ALLERGIKLINIK
John Arnved
Speciallæge i:
Intern medicin
Medicinske
lungesygdomme
Allergi diagnostik

3 Signature Page

Principal Investigator	John Arnved<br>Pulmonology and Allergy<br>Clinic Copenhagen	20/2 08<br>Date and signature
Investigator	Steen Rønborg<br>Pulmonology and Allergy<br>Clinic Copenhagen	20/2 -08<br>Date and signature
Study Director	Peter Bager<br>Department of Epidemiology<br>Research<br>Statens Serum Institut	Date and signature
Data manager	Jan Wohlfahrt<br>Department of Epidemiology<br>Research<br>Statens Serum Institut	Date and signature
Sponsor	Mads Melbye<br>Department of Epidemiology<br>Research<br>Statens Serum Institut	Date and signature

Frederiksborggade 15, 7.
1360 København K
Tlf. 33 13 65 13
Fax: 33 13 65 03
Giro: 9 280 715
john.arnved@dadlnet.dk

3 Signature Page

Principal Investigator	John Arnved<br>Pulmonology and Allergy<br>Clinic Copenhagen	....<br>*Date and signature*
Investigator	Steen Rønborg<br>Pulmonology and Allergy<br>Clinic Copenhagen	....<br>*Date and signature*
Study Director	Peter Bager<br>Department of Epidemiology<br>Research<br>Statens Serum Institut	20/2-08 [signature]<br>*Date and signature*
Data manager	Jan Wohlfahrt<br>Department of Epidemiology<br>Research<br>Statens Serum Institut	....<br>*Date and signature*
Sponsor	Mads Melbye<br>Department of Epidemiology<br>Research<br>Statens Serum Institut	....<br>*Date and signature*

3 Signature Page

Principal Investigator	John Arnved<br>Pulmonology and Allergy<br>Clinic Copenhagen	..........................<br>*Date and signature*
Investigator	Steen Rønborg<br>Pulmonology and Allergy<br>Clinic Copenhagen	..........................<br>*Date and signature*
Study Director	Peter Bager<br>Department of Epidemiology<br>Research<br>Statens Serum Institut	..........................<br>*Date and signature*
Data manager	Jan Wohlfahrt<br>Department of Epidemiology<br>Research<br>Statens Serum Institut	Jan Wohlfahrt, 20/2-08<br>..........................<br>*Date and signature*
Sponsor	Mads Melbye<br>Department of Epidemiology<br>Research<br>Statens Serum Institut	19/2 08 M. Melbye<br>..........................<br>*Date and signature*

3 Signature Page

Principal Investigator	John Arnved<br>Pulmonology and Allergy<br>Clinic Copenhagen	*(abroad on submission November 1, however, a signed statement will be send to DKMA before November 15, 2007)*<br>6/11 07
Investigator	Steen Rønborg<br>Pulmonology and Allergy<br>Clinic Copenhagen	1/11-07<br>*Date and signature*
Study Director	Peter Bager<br>Department of Epidemiology Research<br>Statens Serum Institut	1/11 07 Peter Bager<br>*Date and signature*
Data manager	Jan Wohlfahrt<br>Department of Epidemiology Research<br>Statens Serum Institut	1/[illegible] 07 Jan Wohlfahrt<br>*Date and signature*
Sponsor	Mads Melbye<br>Department of Epidemiology Research<br>Statens Serum Institut	1/11 07 M. Melbye<br>*Date and signature*

4 Addresses

Principal Investigator	John Arnved<br>Pulmunology and Allergy Clinic Copenhagen<br>Frederiksborggade 15, 7th floor<br>DK-1360 Copenhagen	Phone: +45 3313 6513<br>(Direct): +45 3313 6502<br>Fax: +45 3313 6503<br>e-mail: john@arnved.dk<br>john.arnved@dadlnet.dk
co-Investigator	Steen Rønborg<br>Pulmunology and Allergy Clinic Copenhagen<br>Frederiksborggade 15, 7th floor<br>DK-1360 Copenhagen	Phone: +45 3313 6513<br>(Direct): +45 3393 1875<br>Fax: +45 3313 6503<br>e-mail: steron01@mail.dk
Study Director	Peter Bager<br>Department of Epidemiology Research<br>Statens Serum Institut<br>Artillerivej 5<br>DK-2300 Copenhagen S	Phone: +45 3268 8327<br>Fax: +45 3268 3165<br>e-mail: pbg@ssi.dk
Safety & Regulatory Officer	Camilla Panduro Pretzmann<br>Statens Serum Institut<br>Artillerivej 5<br>DK-2300 Copenhagen S	Phone: +45 3268 3933<br>Fax: +45 3268 3973<br>e-mail: cpr@ssi.dk
Study Monitor	Kristina Devantier<br>GCP-unit of Copenhagen University Hospital<br>Amtssygehuset i Gentofte, opg. 15, 2<br>Niels Andersens Vej 65<br>2900 Hellerup	Phone: +45 3977 7279<br>Fax: +45 3965 4156<br>e-mail: kridev01@<br>geh.regionh.dk
Study Research Nurse	***PENDING***<br>Department of Epidemiology Research<br>Statens Serum Institut<br>Artillerivej 5<br>DK-2300 Copenhagen S	Phone: +45 3268 3268<br>Fax: +45 3268 3165<br>e-mail:
Data manager	Jan Wohlfahrt<br>Department of Epidemiology Research<br>Statens Serum Institut<br>Artillerivej 5<br>DK-2300 Copenhagen S	Phone: +45 3268 3952<br>Fax: +45 3268 3165<br>e-mail: jaw@ssi.dk

Sponsor representative	Mads Melbye<br>Department of Epidemiology Research<br>Statens Serum Institut<br>Artillerivej 5<br>DK-2300 Copenhagen S	Phone: +45 3268 3163<br>Fax: +45 3268 3165<br>e-mail: mme@ssi.dk
Collaborator	Tine Westergaard<br>Department of Epidemiology Research<br>Statens Serum Institut<br>Artillerivej 5<br>DK-2300 Copenhagen S	Phone: +45 3268 3156<br>Fax: +45 3268 3165<br>e-mail: twe@ssi.dk
Collaborator	Stig Thamsborg<br>Faculty of Life Sciences, Dep. Veterinary Pathobiology (The Royal Veterinary and Agricultural University, KVL)<br>Bülowsvej 17<br>DK-1780 Frederiksberg C	Phone: +45 3533 3778<br>Fax: +45 3533 2755<br>e-mail: smt@life.ku.dk
Collaborator	Allan Roepstorff<br>Faculty of Life Sciences, Dep. Veterinary Pathobiology (The Royal Veterinary and Agricultural University, KVL)<br>Bülowsvej 17<br>DK-1780 Frederiksberg C	Phone: +45 3533 2746<br>Fax: +45 3533 2755<br>e-mail: aro@life.ku.dk
Manufacturer (supplier/product Insurance)	Ovamed GmbH<br>Att: Detlev Goj<br>Kiebitzhörn 33-35<br>22885 Barsbüttel<br>Germany<br>**Qualification**: GMP certified for TSO manufacture	Phone: +49 40-67 50 95-0<br>Fax: +49 40-67 50 95-59<br>e-mail: gojd@ovamed.de<br>gojd@ovamed.org
Manufacturer	Parasite Technology AB<br>Att: Christian Kapel<br>Faculty of Life Sciences, Dep. Veterinary Pathobiology (The Royal Veterinary and Agricultural University, KVL)<br>Bülowsvej 17<br>DK-1780 Frederiksberg C<br>**Qualification**: GMP certified for TSO rawmaterial manufacture	Phone: +45 3533 2690<br>Fax: +45 3533 2755<br>e-mail: chk@life.ku.dk

Laboratory 1	Phadia AB<br>Att: Bjarne Kristensen<br>Gydevang 33<br>DK-3450 Allerød<br>**Qualification:** In the process of being accredited by DANAK, and fulfils the audit criteria of an international medical company subcontracting clinical trials.	Phone: +45 3264 3201<br>Fax: +45 7023 3307<br>e-mail: bjarne.kristensen@ phadia.com<br>CVR.NR: 2764 0745
Laboratory 2	Lars K. Poulsen, Ph.D.<br>Head of Research, Professor<br>Allergy Clinic, FIN 7551<br>National University Hospital<br>Blegdamsvej 9<br>DK-2100 Copenhagen Ø<br>Denmark<br>**Qualification:** Accredited by Joint Commision as required by the hospital.	Phone: +45 3545 7356<br>Fax: +45 35 45 75 81<br>e-mail: lkpallgy@mail.dk<br>CVR.NR: 1768 4779
Laboratory 3	Københavns Praktiserende Lægers Prøvelaboratorium (KPLL)<br>Pilestræde 65<br>DK-1112 Copenhagen K<br>Denmark<br>**Qualification:** DANAK (reg. nr. 434)	Phone: +45 3374 4000<br>Fax: +45 3374 4001<br>e-mail: kpll@kpll.dk
Chief pharmacist	Jytte Bjerregaard<br>Pharmacy Services<br>Faculty of Life Sciences, Dep. Veterinary Pathobiology (The Royal Veterinary and Agricultural University, KVL)<br>Bülowsvej 17<br>DK-1780 Frederiksberg:<br>**Pharmacy number:** 38200<br>**Qualification:** The Pharmacy is run by the rules pertaining to hospital pharmacies thereby allowing participation in clinical trials following §39 in "lægemiddelloven" (i.e. GCP and Danish regulations). External services provided to research institutions are specifically included according to order no. 857 of October 1, 2001, Ministry of Health.	Phone: +45 3533 3150<br>Fax: +45 3533 2755<br>e-mail: jbe@life.ku.dk

Emergency telephone numbers:

**Study Director**	Peter Bager	Office:<br>Mobile:<br>e-mail:	+45 3268 8327<br>+45 2671 3178<br>pbg@ssi.dk
**Serious Adverse Event Notification**		Fax:<br>Mobile:<br>e-mail:	+45 3268 3973<br>+45 2298 5583<br>pvssi@ssi.dk

5 Introduction

The investigational product is consisting of living embryonated eggs of the intestinal pig whipworm *Trichuris suis,* which belongs to the animal order Helminths in the phyla Nematoda (roundworms). The common name is "egg of the porcine whipworm" (In Danish; "æg fra grisens piskeorm"). The name of the pharmaceutical product is *Trichuris Suis* ova or "TSO®", and the ova are defined as the active substance.

The background for performing research with TSO® offsets in an increasing number of high-ranking publications in recent years suggesting that helminth parasites have a unique capacity to decrease hyper-reactive immune responses,[1-9] such as occurs in IBDs and allergic phenotypes (atopy, food allergy, asthma, allergic rhinitis, and atopic dermatitis). The relevance for allergic diseases should be seen in the context of the so-called hygiene hypothesis,[10] which suggests that the allergy epidemic[11-13] in affluent countries is the result of more hygienic lifestyles leading to loss of microbial stimulation, which previously protected children from developing allergic diseases. Allergy research has focused intensely on the possible preventive and therapeutic effects of micro-organisms or microbial products;[14] so far helminths have not been put on trial. However, observational studies on microbial exposures have most consistently reported that particularly helminth infection is associated with a reduced risk of atopy and allergic disease.[3;4] The observations are much in line with the hygiene hypothesis, because helminth infections are most common in developing countries, where allergy is rare and hygiene poor. In addition, a cause-effect is made plausible by several biological observations:

- Helminth infection presents with a high level of IgE antibody in serum otherwise only seen in allergic disease. However, in allergic individuals, IgE against pollen triggers allergic inflammation and symptoms, while helminth infection is often asymptomatic due to a state of hyporesponsiveness to the helminths and bystander antigens, such as allergens.[15]

- Infection with helminth with widely different life-cycles and species morphology has been associated with reduced risk of atopy (e.g. schistosomiasis and hookworm infection) suggesting a common biological mechanism.[3;4]

- Both allergic reactions and helminth infection are characterised by a so-called Th2 cytokine profile (IL-4, IL-5, and IL-13). However, during helminth infection this profiles is modified by production of the cytokines IL-10 and TGFβ by regulatory T- helper lymphocytes, factors that are believed to control inflammatory reactions.[7;15]

- The state of hyporesponsiveness during helminth infection presents immunological characteristics,[15] which are similar to those observed during effective allergen immune therapy for allergic rhinitis, including detectable production of the cytokine IL-10,[3;16] specific IgG4, and high ratios of IgG/IgG4 to antigens/allergens.[17-19]

- Murine models of helminth infection and allergic diseases support the above observations in humans.[20-22]

Observational studies have mostly focused on asthma and helminths, and these studies further suggests that the observed reduction in risk of asthma might be stronger for infection with helminth species with a systemic phase in their human host, and/or to be

strengthened with increasing intensity of infection measured by number of eggs in feces.[4] Currently, it is believed that helminths or their excretory products carry signature molecules that are particular suitable for the natural induction of a robust anti-inflammatory regulatory network that could prevent or alleviate symptoms of allergic disease.[7] Research is greatly motivated by the fact that successful intervention studies with helminths are likely to focus the search for such potential signature molecules.

Evidence for a direct cause-effect between helminth infection and reduced risk of allergic disease has been suggested by studies showing that anti-worm treatment in endemic areas effectively removes high worm burdens but such treatment is temporally associated with an increase in positive skin reactions to allergens (atopy).[23;24] In April 2006, a large trial published in Lancet showed that although moderate worm burdens was associated with a reduced the risk of atopy, randomised worm treatment did not reverse this effect.[5] An allergy expert[14] in the hygiene hypothesis commented that intervention studies adopting original and safe approaches now are required to bring this field further[25] Accordingly, already in 2004, a trial with *T. suis* was suggested in Science.[2] Since 2004, phase I-III trials with hookworms have become ethically approved in UK.[26] Phase I trials are reported to be completed[27] and II commenced.[28] One subject with self-reported asthma claimed effective results in a deliberate self-treatment with hookworm.[29] Hookworms, however, might be invasive and high dosages can cause anemia.

In contrast, members of helminth family *Trichuris* are intestinal whipworms with favourable characteristics for therapeutic use;[30;31] they are non-invasive, are superficially attached to the intestinal mucosa, do not multiply in the host, and have no migratory stages. The investigational helminth, *Trichuris suis*, is genetically related to *Trichuris trichiura*, the human whipworm, but it is not a human pathogen. It has been shown experimentally to colonise humans very briefly without causing disease.[32-36] TSO® can be produced using pigs grown in a pathogen controlled environment, and processed to assure absence of biological contaminants. *Trichuris suis* ova has been shown to be safe and effective therapy in treatment of IBDs in three clinical trials in USA (phase I and II).[34-36] A total of 90 patients were treated 3-6 months, and some were concurrently treated with moderate dose corticosteroids, azathioprine, 6-MP and/or other immunosuppressants. There have been no adverse events reported attributable to TSO®. Thus, systemic infection or invasion has not been seen, despite the severity of the underlying colonic problems with IBDs, and in accordance with the natural life cycle of whipworms. In Europe, protocols for TSO® treatment of food-allergic children[37] and IBD patients are being developed (see www.ovamed.de). The sponsor of the present trial has chosen a phase II design with 100 participants, because moderate or severe adverse effects is not expected in the chosen study population, whereas any efficacy is likely, of great interest, and unknown. To measure efficacy of pollen-allergy, then a relatively large number of participants are preferable. In these terms, the population size is quite small, however, of a size comparable with other immune therapy trials for allergic rhinitis, and with sufficient statistical power.

TSO® and placebo is supplied for the present trial by the pharmaceutical company Ovamed GmbH in Germany. The rawmaterial for TSO® is manufactured by the Danish company Parasite Technology ApS at the fascilities of KVL (scientific collaboration),

and processed into the investigational medicinal product by Ovamed GmbH. The manufacturing process at Parasite Technology ApS has been audited, and is certified by the DKMA, and Ovamed GmbH is GMP certified for TSO production. The ongoing preclinical and the planned clinical development (first trial planned in august 2008) of the TSO® for marketing as IBD treatment is performed by the RDC company. Their preclinical toxicology program includes repeat dose toxicity studies in cynomolgous monkeys and rabbits, which have caused no safety concern. TSO® has been evaluated by EMEA on June 2, 2006, and their advice is taken into account in the IB and IMPD submitted to the DKMA by the sponsor, SSI. The investigational trial is a non-commercial, and researcher-initiated study, which will be initiated and leaded by SSI. SSI's interest is purely scientific with the innovative aim of exploring TSO®'s potential in treatment of allergic rhinitis in a clinical trial.

The following guidelines have been used in particular when preparing the protocol:

- Note for guidance on good clinical practice (CPMP/ICH/135/95).
- Guideline on the clinical development of medicinal products for the treatment of allergic rhinoconjunctivitis (CHMP/EWP/2455/02).
- Note for guidance on the clinical investigation of medicinal products in the treatment of asthma (CPMP/EWP/2922/01).
- Note for guidance on statistical principles for clinical trials (CPMP/ICH/363/96).
- Points to consider on missing data (CPMP/EWP/1776/99).

6 Trial objectives

6.1 Primary objective

The primary objective is to assess the efficacy of immune stimulation by transient *Trichuris suis* infestation of the gut on self-reported symptoms of allergic rhinitis and number of well-days among adults with allergic rhinitis.

6.2 Secondary objective

The secondary objective is to assess the efficacy and safety of immune stimulation by transient *Trichuris suis* infestation of the gut on allergic rhinitis and asthma assessed through self-reported disease activity, clinical skin and airway activity, and immunological activity among adults with allergic rhinitis.

6.3 Primary variables

The primary variables will be based on participants' diaries. Details of the definition of the variables are described in the table 1 and 2 in section 12.4. The two primary variables comprise:

A. Average daily score for symptoms of allergic rhinitis, which include symptom A+B+C, or A+B+C+D as defined in table 1 in section 12.4.

B. Number of well days, as defined in table 2 in section 12.4.

6.4 Secondary variables

The secondary efficacy and safety variables will be based on self-reported disease activities related to allergic rhinitis, asthma and adverse events (diaries). Furthermore, on clinical skin and airway activity (skin prick tests, lung function, and degree of airway inflammation).

Details of the definitions of variables (e.g. standardised scoring systems) are described in the table 3, 4, and 5 in section 12.4. The secondary variables comprise:

Efficacy variables

Self-reported disease activity related to allergic rhinitis (diaries)

a. Daily recording of use of medication for allergic rhinitis, calculated as a score during data management.
b. Daily score for all symptoms of allergic rhinitis on a 100mm visual analog scale (VAS).
c. Assessment at the end of the trial of all symptoms of allergic rhinitis during the trial when compared with symptoms during the previous pollen season.
d. Weekly score for quality of life using a validated Rhinitis Quality of Life Score.

Clinical skin and airway activity

e. Skin test reactivity to inhalant allergens.
f. Degree of airway inflammation measured by amount of exhaled nitrogen oxid into a Niox apparatus.

Immunological activity

g. Reactivity of sIgE and level of sIgG/sIgG4 to inhalant allergens measured by ImmunoCAP™.
h. Cytokines (IL-4, IL-5, IL-13, IL-10, and TGFβ) measured as *in vitro* production from peripheral blood mononuclear stimulated with allergens and *Trichuris suis* antigen. The analyses will be performed for 30 (2x15) participants (see section 8.1.5).

Efficacy and safety variables

Self-reported disease activity related to asthma and adverse events

i. Daily recording of use of medication for asthma, calculated as a score during data management.
j. Daily score for symptoms of asthma.
k. Daily adverse events (safety variable)

Clinical airway activity

l. Daily lung function measured as forced expiratory volume 1 (FEV1, safety variable) and peak expiratory force (PEF) using a pocket flow-meter (Piko-1).

The secondary safety variables j, and l are included as safety variables not because the active treatment is suspected to influence these variables negatively, but because the

duration of the investigational treatment by definition corresponds to chronic treatment (>6 months). It therefore is reasonable to asses whether it is safe for the indication allergic rhinitis, i.e. the only potential life-threatening allergy associated with allergic rhinitis is asthma. The long duration of the treatment also makes it reasonable to assess efficacy for the same variables as well as for variable i, although the study is not designed to have the statistical power to do so.

The secondary safety variable (k) for adverse events, include events that are presumable reasonable to study, although no such adverse events have been reported to be due to TSO® treatment: Diarrhoea, anal pruritis, and flatulence. These are among the few symptoms in pigs - the natural host of *Trichuris suis* – that are exposed to extreme numbers of eggs when compared with the investigational dose in man (i.e. pr kg). In addition, they are among the symptoms associated with human *Trichuris trichiura* infection.

7 Investigational plan

7.1 Overall design

A single-centre, triple-blinded placebo-controlled phase II trial with 100 adult male and non-fertile female volunteers allergic to birch and/or grass pollen. Volunteers will be recruited at Denmark's largest allergy clinic (the PACC) after a 2-month pre-trial screening and thereafter randomised to receive treatment every 3rd week for 24 weeks with 2500 TSO® and placebo. The dose and interval has been used in preclinical and clinical studies and without any concerns for safety. Furthermore, the longest duration in the clinical trials were also 24 weeks, and here 70-80% of patients with Crohn's disease responded to the treatment.[34-36] TSO® and placebo has the same taste, smell, and absence of immediate effect. The appearance of TSO® is slightly different (eggs are slightly visible as a turbid sedimentation after few seconds of immobility), and therefore treatment bottles are dark-coloured to conceal the content and intake is directly from the bottle. Treatment is to be received at the clinic in the presence of the Investigator.

Participants will be asked to withstand use of preventive steroids, and avoid any use of medication for allergic rhinitis, as long as symptoms are tolerable. The benefits for all participants are that no side effects are expected due to TSO, whereas several allergy medications and therapies have long-term side effects: i.e. immune therapy with allergens can cause oral pruritis in between 40-70% of treated patient, steroids can be immuno-suppressive (on the doping list), and antihistamines can cause drowsiness. In addition, education through monitoring of their disease during trial is expected to increase their disease control. No further quantitative and qualitative risk-benefit assessment can be performed because this is the first time allergy is treated with TSO. The participants will have the advantage that their symptoms of allergic rhinitis might be reduced. Medication for allergic rhinitis and asthma (relief medication) is provided by the PACC in order to uniform any medical treatment (different allergy medications have different duration of effect).

All participants are to receive 8 doses in 3-week intervals. Thus, the duration of the trial for each participant is 24 weeks. The screening dates and inclusion dates (see calendar at start of section 9) are planned so that all participants have been treated at least 2 weeks before the grass pollen peaks arrives. The choice of 2 weeks is based on a 2-4 weeks lag time for a response reported for *individual* participants in a previous trial, where IBD patients were treated with TSO. In a second trial, it was shown that the majority of IBD patients responded to the treatment after 6-8 weeks.[34;35] Overall, however, it is expected that the majority of the 100 participants can be started up before April 5 (primary recruitment group), and thus have at least 8 weeks of 3-weekly treatment before the grass pollen season peaks. However, later start-up is allowed merely to increase the possibility that 100 patients can be included in the trial (secondary recruitment group).

The repeating of treatment is chosen because the two previous trials[34;35] showed maintainance therapy was required for effective treatment of IBD.

It is unknown whether the above choices on pre-treatment period before pollen seasons are an advantage in the treatment of allergic rhinitis, however, it is the optimal design considering the currently available knowledge on TSO treatment.[§]

§ Note: The trial can not be started early enough for all participants to be treated for min. 2 weeks prior to the birch pollen peak (which arrives before the grass peak).The potential loss of the detection of a TSO-effect on birch pollen symptoms is not likely to be crucial for the statistical power of the efficacy analyses, because very few clinical trials on immune therapy for allergic rhinitis have been able to show an effect purely using data on birch pollen symptoms. This was partly because the birch season is very short, the peak is very variable, and sometimes absent (e.g. in Viborg in 2005 the birch pollen season was almost absent). In contrast, the grass pollen season is usually longer, its time of peak more constant, and grass pollen seasons have not been absent in Denmark as judged from pollen counts from 2003 to 2007 in Copenhagen and Viborg.

Furthermore, for some participants in the secondary recruitment, it is possible that a minority will receive up to 4 of the 8 doses *after* the pollenseason of grass has ended. This is allowed in order to increase detection of a treatment effect on other allergic symptoms (asthma and other pollen allergies, e.g. mugwort season), and to increase the quality of a potential follow-up in the next pollen-season – i.e. all participants will have received the same number of doses when followed up.

Baseline is established through the pre-trail screening, patient journals, baseline questions, and measurements at the first visit. The baseline questions include questions on sex, age, height, weight, occupation, ethnic origin, smoking, pet keeping, antibiotic and fungal treatment within the last 3 weeks, parental allergic diseases, past and current allergic diseases, symptoms and triggers of allergic rhinitis, medication for allergic rhinitis (strength, daily dosis, treatment period), classification of allergic rhinitis (ARIA classification), and use of medication other than the mentioned.

Participants will be asked to keep a diary in which they use standardised scoring systems to score daily symptoms of allergic rhinitis and asthma (4-point scale), and weekly quality of life (6-point scale).[38;39] They are also to record adverse events, as well as use of relief medication –the latter will be calculated as a score during data management. Daily lung function is to be measured at home using an electronic pocket-flowmeter. Data on FEV1 from the device is recorded in the diary thus fascilitating quick safety assessment for the Investigator at a visit, and all data in the

device (FEV1, PEF) will be downloaded by SSI to a dedicated computer every 3-4th week due to the limited memory of the device. In the mean time a second device is used by the participant (all will have 2 devices).

At start, middle, and end of the trial, the Investigator measures participant's airway inflammation by exhaled nitrogen oxid into a Niox apparatus at the clinic, and collect blood samples to be analysed in external laboratories for sIgE, sIgG, and sIgG4 reactivity to allergens, and for cytokine responses possibly associated with therapeutic activity.

At start and end of the trial, skin reactivity to allergens will also be tested.

At the end of the trial, participants assess globally their symptoms of allergic rhinitis when compared with the previous pollen season.

The follow-up period will be assessed by participants in the last part of their diaries and will include an assessment of symptoms and medication for allergic rhinitis and asthma since the trial ended. The diary will be handed in at the follow-up visit at the clinic.

Every 3rd week during the trial, the Investigator assess the safety of the treatment through evaluation of recorded adverse events, which will be restricted to daily records of diarrhoea, anal pruritis, flatulence, asthma symptoms, asthma relief medication, and measurements of lung function. Serious adverse events are reported within 72 hours to the senior medical advicer at SSI (e.g. not mild diarrhoeas), and if the investigator judges it is necessary, a similarly immediate reporting to SSI should be done for non-serious adverse events if unexpected and/or of moderate to severe intensity (The severity will be assessed by the Investigator based on diaries and an overall assessment of the patients)

The trial personal at the clinic includes the Investigator, a co-Investigator, and 2 clinical nurses. The trial personal from the Sponsor (SSI) includes a Study Director, a Monitor, a research nurse, and a Data Manager (statistician).

The data management is performed by SSI. Thus, after all participants have completed every 3rd visit, all source documents (including CRFs) are transported to SSI (diary, skin test, NO, and lung function data).Source data from blood analyses is transported from external laboratories to SSI, i.e. they are not entered into the CRFs.

At SSI, the data from source documents are entered into SAS data sets, which are subsequently checked for consistency, and proofread against the source documents – all done by the Study Director, the Data Manager (statistician), and the research nurse.

Monitoring include an initiation and close-out visit at Investigator's and Sponsor's site, and regular monitoring visits each month during the trial.

To maintain blinding during statistical analyses of the data, the Data Manager will only know the allocation *groups*, while the code for the allocation of *active* and *placebo* groups is first to be broken after the follow-up period and close-out of sponsor's site. However, blinded statistical analyses will start already after close-out of the Investigator's site (last end visit). The Data Manager finishes the blinded data analyses according to the protocol, and unblinds the treatment groups after close-out of the sponsor's site. If an effect of TSO is observed then blood samples will be send for analyses, and if not then the decision to do the analyses will be postponed.

7.2 Study population

The study population consists of a 100 voluntary male and non-fertile female adults with allergic rhinitis.

7.3 Recruitment of volunteers

The volunteers will be recruited by the Principal Investigator and co-Investigator based on a 2-month pre-trial screening of patients at the PACC, and for most patients' medical records at the clinic dating back to at least the previous pollen season will be checked against the inclusion/exclusion criteria (the procedure have been approved by the Ethical Committee). The Investigator will keep a screening log of potential volunteers.

It is expected that all 100 volunteers will start up trial during 4 weeks from March 10 to April 4 (primary recruitment group), however, after that and before May 24, partipants can still be recruited (secondary recruitment group). See trial calendar at start of section 9 for a better overview).

7.4 Inclusion criteria

1. Male or non-fertile females aged $\geq$18 and $\leq$65 years. Females must be post-menopausal or sterilised to be categorised as non-fertile.
2. Symptoms of grass pollen allergy the last 2 pollen seasons, or more.
3. Forced Expiratory Volume 1 >70% of expected.
4. Scoring all symptoms of allergic rhinitis >50 mm on a 100 mm VAS (0=not bothersome, 100=worst) during previous pollen season.
5. Specific IgE class $\geq$2 to allergen extracts of grass ($\geq$0.7 $kU_{allergen}$/l).
6. A positive skin prick test ($\geq$3mm) to allergen extracts of grass.
7. Prepared to grant authorized persons access to medical patient records at PACC.
8. The volunteer is likely to comply with instructions (willing to participate for 24 weeks, visit the clinic, make the diary, and measure daily lung function).

For a volunteer to be included in the trial, all of the criteria listed above must be answered with a "yes".

7.5 Exclusion criteria

9. Significant asthma.
10. Use of systemic steroids during the last 2 months.
11. Immune therapy for grass pollen allergy the last 2 years.
12. Planning a travel abroad during the trial period (excluding areas with similar grass and birch pollen counts when compared with the Copenhagen area).
13. Past or current severe diseases (a history of Crohn's disease, ulcerative colitits, multiple sclerosis, active hepatitis B or C, cytomegalovirus, herpes simplex, HIV, other kinds of immune deficiency, and cancer).
14. Anti-helminth treatment within the last 2 weeks (e.g. vermox, mebendazol, albendazol).

15. Known or possible hypersensitivity to *Trichuris* species, or compounds made of *Trichuris* species.
16. A past or recent drug abuse.
17. Participation in other clinical trials.
18. Employed at the PACC (center of trial), or the Department of Epidemiology Research at SSI (sponsor's department).

7.6 Withdrawal and predetermined reasons for discontinuation

Volunteers are free to withdraw from the trial whenever they desire.

If, for any reason, a volunteer wishes to discontinue his/hers participation in the trial, or if, according to Sponsor's/Investigator's judgement, he/she must be withdrawn from the trial, the date and reason for the withdrawal are to be recorded in the CRF. Data from volunteers that are withdrawn from/discontinue the trial will be included in the final report.

Volunteers that are to be withdrawn from participation in the trial, wishes to discontinue participation in the trial, or in either case by reasonable way could discontinue only the investigational treatment, will always as minimum be followed up as described in section 8.1.8 (Precautions).

If participants discontinue only the investigational treatment, the Investigator should make an effort to follow the compliance procedures described in section 8.1.6 (Compliance and drug accountability).

Volunteers will not be replaced.

The trial can be terminated at any time if Sponsor or Investigator determines that the trial poses a serious threat to the volunteers.

8 Investigational products

8.1 Treatments administered

The investigational active product is a non-sterile, aqueous suspension of 2500 viable purified, embryonated *Trichuris suis* eggs in a volume of 15 (±1) ml with a concentration of 167 embryonated TSO per ml suspension base of sulphate-stabilised 0.1 N sulphuric-acid buffer (pH 1).

The investigational placebo product is an aqueous suspension of sulphate-stabilised 0.1 N sulphuric-acid buffer (pH 1) in a volume of 15 (±1) ml

Blinding is achieved because TSO® and placebo dosages have identical taste, smell, and absence of immediate effect. The TSO eggs are slightly visible as a minor turbid sediment, and therefore dark-coulered immediate containers is used to conceal the content.

The investigational products will be administered as one dose every 3rd week during the trial. The number of dosages to be admininstered to one participant during the trial is 8 dosages and no more.

The ingredients, concentration, and volume of the investigational products are:

	**TSO®**	**Placebo**
Dose	2500 eggs	None
Concentration of eggs	167 eggs/ml	None
Impurity	Non-embryonated eggs<br><50% of eggs	None
Buffer system	Sulphate-stabilised sulphuric acid, 0.1 N (pH=1)	Sulphate-stabilised sulphuric acid, 0.1 N (pH=1)
Excipients	Na (sodium)<br>K (potassium)<br>Sulphate	Na (sodium)<br>K (potassium)<br>Sulphate
Volume	15 ml	15 ml
Immediate container (bottle)	60 ml dark-coloured bottle with screw cap	60 ml dark-coloured bottle with screw cap

Briefly, TSO® is manufactured by Parasite Technology at KVL (rawmaterial), and at Ovamed GmbH in Germany. Ovamed GmbH will also manufacture the placebo product, and provide both products for the trial free of charge. Both products are produced according to GMP. Active and placebo investigational products will be transported from Ovamed GmbH to the Pharmacy Services at KVL, and from Pharmacy Services to the PACC. Temperature loggers will be used during all transport.

8.1.1 Dosages and administration

The investigational 2500 TSO® and placebo dosages is to be administered orally in the presence of the investigator every 3rd week during the trial.

The active product has no taste, smell, and absence of immediate effect different from that of the placebo product.

To achieve blinding, dark-coloured bottles are used, and participant are to drink directly from the bottle *after* the Investigator has neutralised the sulphuric acid by adding 200(±50) mg sodiumhydrogencarbonate ($NaHCO_3$) powder into the bottle. This will raise the pH from 1 to pH 6-9, which is drinkable.

The powder will either be delivered in boxes (GMP produced pure $NaHCO_3$), or in letters with the product Samarin (213mg $NaHCO_3$ with uvic and citric acid). If delivered in boxes then measuring spoons and stiring sticks are supplied by SSI. A total of 175g is needed for the entire trial.

INSTRUCTION FOR TSO:

- Treatment bottle
- Sodium hydrogencarbonate powder
- Measuring spoon
- A glass of water for the participant

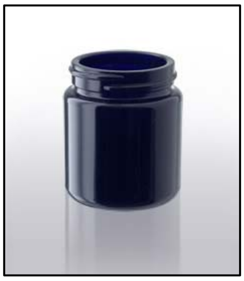

Bottle unscrewed

Products should be administered on an "empty stomach", and as follows:

1. Unscrew the cap. Apply one measuring spoon of sodium hydrogencarbonate powder (a little fizz and foam occurs). Screw the cap back on and shake the bottle.
2. Unscrew the cap (a little fizz and foam occurs). Ask the participant to drink the content ***directly*** from the bottle. Offer the participant a glass of water afterwards.
3. After the drinking, screw the cap back on, and write date and initials on the label. Used bottles are to be accounted for, and must be put back in the refrigerator.
4. Assure that the participant feels comfortable with the treatment.

8.1.2 Packing and labelling

Packing and labelling takes place in two steps; one step pertaining to the transport from Ovamed GmbH to the Pharmacy Services at KVL, and one step pertaining to the transport from the Pharmacy Services at KVL to the PACC.

For the transport from Ovamed GmbH to KVL: The investigational products will be labelled and packed at Ovamed GmbH according to internal procedures and so that boxes with packages of active and placebo products are packed separately. The products will then be transported to the Pharmacy Services at KVL in Denmark.

Ovamed's labels on the immediate containers of the IMP and placebo will be in English and appear as below. The labels will have a blinding code for IMP and placebo. The purpose is to maintain blinding of participants/doctors, when Pharmacy Services later put labels on top of Ovameds labels (possibly transparent labels). If possible, however, the Pharmacy Services will remove Ovamed's labels before putting on their own.

Ovamed's labels on the immediate containers of the IMP and placebo:

IMP	Placebo
Batch no.: [IMP no.]	Batch no.: [placebo no.]

Ovamed's labels on the outer packing and of boxes with packages will be in English and appear as below:

IMP	Placebo
For clinical trial use only<br>Manufacturer: Ovamed GmbH<br><br>INVESTIGATIONAL<br>MEDICINAL PRODUCTS<br><br>XX bottles, which contains 15 ml of sulphuric acid (pH=1) with 2500 TSO®<br><br>**Storage**: 2-8 ºC.<br>Avoid freezing or heating.<br>**Batch no.**: [IMP no.]<br>**Expiry date**: [ddmmyyyy]	***For clinical trial use only***<br>Manufacturer: Ovamed GmbH<br><br>PLACEBO<br>PRODUCTS<br><br>XX bottles, which contains 15 ml of sulphuric acid (pH=1)<br><br>**Storage**: 2-8 ºC.<br>Avoid freezing or heating.<br>**Batch no**.: [placebo no.]<br>**Expiry date**: [ddmmyyyy]

For the transport from KVL to PACC: KVL performs labelling, and packing of all treatment bottles into packages and packages into boxes. The task will be performed by Chief Pharmacist Jytte Bjerregaard at the Pharmacy Services at KVL. The Pharmacy Services is approved by the DKMA to perform the task. All packages will be packed in boxes and transported to the PACC. Temperature loggers will be packed with the products and collected at arrival to PACC.

Ready-for-treatment-labelling: Batch no. will not be indicated on any labels because the pharmacist will have information on participant no. linked to batch no. via the master randomisation list.

Leaflet and packages for self-administration: A participant who self-adminster the treatment is provided with a 0.5 liter steel thermo-can containing a labelled treatment bottle. The can will contain a leaflet for self-administration + powder for neutralisation. The participant is to keep the leaflet in his/hers possession at all times until next visit at the PACC. (The CRF will contain a check-list for selftreatment including packing instructions and recording of phone numbers for the day of self-treatment.)

Pharmacy Services' labels for immediate containers (bottles) will be in Danish and appear as below:

In Danish	In English
Kun til klinisk forsøg<br>***Læge***: John Arnved; TSO-01.<br>Svovlsyre (pH=1) med 2500 TSO®, eller uden.<br>Oral indtagelse, følg instruks!<br>Mindst holdbar til: [ddmmåååå]<br><br>***DELTAGER NR.:***	***For clinical trial use only***<br>***Investigator***: John Arnved; TSO-01.<br>Sulphuric acid (pH=1) with 2500 TSO®, or without.<br>Oral intake, follow instructions!<br>Expiry data: [ddmmyyyy]<br><br>***PARTICIPANT NO.:***

Pharmacy Services' labels on the outer packing and boxes with packages will be in Danish and appear as below:

In Danish	In English
Kun til klinisk forsøg TSO-01<br>Læge John Arnved (tlf. +45 3313 6502)<br>Lunge- og Allergiklinikken København<br>Klinikken's Tlf.: +45 3313 6513<br>Frederiksborggade 15, 7. sal<br>DK-1360 København K<br><br>XX flasker der indeholder 15 ml svovlsyre (pH=1) med 2500 TSO®, eller uden (placebo).<br><br>***DELTAGER NR: XX***<br><br>***Indtagelse***: Følg indlægssedlen.<br>***Opbevaring:*** Opbevares utilgængeligt for børn. Ved 2-8 ºC i køleskab. Undgå frysning og opvarming.<br>***Anvendes før:*** [dag, måned, år]	***For clinical trial use only TSO-01***<br>Dr. John Arnved (Phone. +45 3313 6502)<br>Pulmonology and Allergy Clinic Copenhagen<br>Phone no. of clinic.: +45 3313 6513<br>Frederiksborggade 15, 7. sal<br>DK-1360 Copenhagen K<br><br>XX bottles, which contains 15 ml of sulphuric acid (pH=1) with 2500 TSO®, or without (placebo).<br><br>***PARTICIPANT NO.: XX***<br><br>***Administration:*** Follow the leaflet.<br>***Storage:*** Keep out of reach of children. Store at 2-8 ºC in refrigerator. Avoid freezing or heating.<br>***Use before***: [day, month, year]

Leaflet for self-administration of oral treatment (will be in Danish and/or English):

Til klinisk forsøg **TSO-01**
Læger: John Arnved, +45 3313 6502; Steen Rønborg, +45 3393 1875. Sygeplejerske, +45 3391 5669. Klinik:+45 3313 6513
Lunge- og Allergiklinikken København
Frederiksborggade 15, 7. sal, 1360 København K

1 glasbeholder indeholder en sur vandig opløsning med 2500 *Trichuris suis* æg (TSO®), eller helt uden æg (dvs. placebo).

Virkning og anvendelse: TSO® indeholder levende *Trichuris suis* æg, som er den aktive ingrediens. TSO® optages ikke og cirkulerer ikke i kroppen. *Trichuris suis* æg gennemgår omfattende tests for ensartethed og sikker brug. TSO® bruges til kroniske immunologiske betændelses-tilstande, som kan skyldes tab af naturlig eksponering overfor parasit-organismer af typen Helminth. Placebo produktet har ingen virkning.
Instruktion for Indtagelse: Ved indtagelse uden lægen, venligst ring som aftalt og <u>før indtagelse</u> til forsøgets læge/sygeplejerske/klinik. Indtag på ”tom mave”. Skru låget af og tilsæt 1 måleske eller 1 brev pulver (findes i termokanden). Skru låget på, ryst flasken let i ½-1 minut og skru låget af (Opløsningen er nu drikkelig). <u>Derefter d*rik indholdet direkte fra glasbeholderen*</u>. Skyl efter med et glas vand. Husk at skrue låget på, at skrive dags dato på beholderen, og at medtage beholderen ved næste besøg på allergi-klinikken. Lad ikke andre drikke af beholderen. Fortynd ikke indholdet i beholderen med varme drikke (fx kaffe, te), eller alkohol (fx øl, vin, spiritus). Indholder lokalirriterende syre (pH 1).
Opbevaring: Opbevares utilgængeligt for børn. Opbevares ved 2 ºC til 8 ºC i køleskab. Undgå frysning eller opvarmning over 40 ºC. Læg ikke glasbeholder(e) på fryseelementer eller i is der kunne være under 0 ºC. Ved transport, brug udleveret termokande: Nedkøl termokanden i køleskab før transporten, og transporter i op til 6 timer.
Bivirkninger: Ingen kendte. Hvis nogen komplikationer ved et uheld skulle skyldes TSO® vil behandling med f.eks. vermox tabletter (2x200 mg/dag i 3 dage) behandle *Trichuris suis*. Medicinen har ingen større bivirkninger. Kræver recept.
Brug af anden medicin: Undgå brug af antibiotika af typen metrodinazole (flagyl) og cyclosporin da de måske kan dræbe *Trichuris suis*. Brug af anden medicin er tilladt i forsøget.
Behandlingsperiode: 6 måneder i 2008. Max. 8 tre-ugentlige doser er tilladt i forsøget.

For clinical trial use only. **TSO-01**
Doctors: John Arnved, +45 3313 6502; Steen Rønborg, +45 3393 1875. Nurse: +45 3391 5669. Clinic: +45 3313 6513. Pulmonology and Allergy Clinic Copenhagen, Frederiksborggade 15, 7. sal, 1360 Copenhagen K

1 glasscontainer contains an acidic aqueous suspension with 2500 *Trichuris suis eggs* (TSO®), or completely without eggs (i.e. placebo).

Effect and use: TSO® contains living *Trichuris suis* eggs, which is the activie ingrediens. TSO® is not metabolized and does not circulate in the body. *Trichuris suis eggs* are extensively tested for uniformity and safe use. TSO® is used for immune-mediated chronic inflammatory conditions that may result from loss of natural exposure to parasitic organisms of the Helminth type. The placebo product has no effect.
Instruction for Intake: If intaken without the doctor, please phone as scheduled, and <u>before intake</u> to the trial’s doctor/nurse/clinic. To be administrated on an ”empty stomach”. Unscrew the cap and add 1 measuring spoon or 1 letter of powder (present in the thermo flask). Screw the cap back on, shake the glasscontainer slightly for ½-1 min., and unscrew the cap again (the suspension is now drinkable). <u>Afterwards *drink the content directly from the glasscontainer*</u>. Rinse after with a glass of water. Remember to srew the cap back on, write the date on the container, and bring the conainer along at your next visit at the allergiy clinic. Do not let others drink from the container. Do not dilute the content of the container with hot drinks (coffee, tea), or alcohol (beer, wine, liquor). Contains locally irritating sulphuric acid (pH 1).
Storage: Keep out of reach of children. Store at 2 ºC to 8 ºC in refrigirator. Avoid freezing or heating above 40 ºC. Do not place glasscontainer(s) on freezer packs or in ice that could be below 0 ºC. For transport, use thermocan handed out: Cool the thermocan in refrigerator before the transport, and transport in up to 6 hours.
Side effects: None known. Should any adverse events by chance be due to TSO®, then treatment with e.g. Vermox pills (2x200mg/day for 3 days) will treat *Trichuris suis.* The medication has no major side effects. Prescribtion is required.
Concomitant medication: Avoid use of antibiotics of the type metrodinazole (flagyl) og cyclosporin as they might kill *Trichuris suis.* Use of other medication is allowed in the trial.
Treatment period: 6 months in 2008. Max. 8 three-weekly doses are allowed in the trial.

8.1.3 Storage information

Because TSO® contains a living organism, it must be protected from high temperatures or freezing. Do not expose TSO® to temperatures greater than 40°C. Do not freeze TSO®. TSO® must be refrigerated at temperatures between 2°C and 8°C. It should not be stored below 2°C due to the risk of freezing. Do not place the treatment bottles directly on freezer packs or in ice that could be below 0°C. TSO® must not be used after the expiry dates. Investigational products that have been stored differently from recommendations of SSI must not be used.

The placebo product is to be stored under the same conditions as TSO®.

The investigational products should be stored separately or locked away from other medication, and the drug accounatability document must be updated.

The storage temperature for the investigational products will be monitored during all transport, and also at storage in refrigirators at the PACC.

The investigational products must always be used within the same day the cap of the treatment bottle is unscrewed.

8.1.4 Transport of trial products

The investigational products will be packed at Ovamed GmbH and transported to the storage fascilities at the Pharmacy Services at KVL. Temperature loggers will be packed with the investigational products prior to transport for monitoring of the temperature during the transport, and temperature documented by SSI. After KVL have allocated, labelled, and packed the treatment bottles with temperature loggers, SSI will transport the boxes from KVL to the refrigerator at PACC. Temperature loggers must be treated according to the instructions given by SSI.

In the case a few participants cannot attend a visit, then self-treatment is proposed, and a cooled steel thermocan (6 hours temperature retention) is provided for the transport of the product home, and for any further transport carried out by the participant. A checklist for preparing the self-treatment-participant has been implemented in theCRF.

8.1.5 Randomization procedure

The trial is a triple blinded placebo controlled randomised trial. Thus, all participants and personal involved in the trial are unaware of the identity of who receives active and placebo treatment during the trial period and statistical analyses. The randomisation procedure will be performed by the Biostatistical Unit at SSI's Quality Assurance Department (none of the personal at this department are otherwise involved in the trial, the data management, or post-trial statistical analyses). The labelling and packing will be performed by Chief Pharmacist Jytte Bjerregaard at the Pharmacy Services at KVL. Before the trial starts, the Biostatistical unit will prepare the following lists for the trial:

- The master randomisation list (complete randomisation list)
- The blinded allocation list for statistical analyses
- A packing list

All lists can be supplemented by an electronic form, which should be saved on a CD and secured together with any hard copies of the lists.

Briefly, the statistical software SAS will be used for generating all 3 lists. The randomisation will be performed in blocks of a size that both takes into consideration the blinding of the 2 Investigators and the inclusion of 100 subjects. No stratification will be used. A testversion of the lists (and emergency envelopes) will be made and controlled by the dedicated person at the sponsor's Quality Assurance department.

All 3 lists will contain:

- Trial code, TSO-01
- Principal Investigator: Dr. John Arnved, Pulmonology and Allergy Clinic Copenhagen
- Number of subjects randomised
- Block size
- Participants' trial number

And they will be different in the following way

Content of the master randomisation list

- Title: COMPLETE randomisation list
- Seed values used for initialisation of the SAS randomisation program
- Allocation group for each participant

Content of the allocation list for statistical analyses:

- Title: ANONYMISED Randomisation List
- Anonymous allocation group for each participant (e.g. group Z vs. Y, but NOT active vs. placebo).

Content of the packing list:

- Title: "Packing List: Placebo" and "Packing List: Trichuris suis ova (Active)"
- Seed values used for initialisation of the SAS randomisation program
- Dose no. (space for the pharmacist to write date and initials for each packing)

Decoding envelops in two sets

Labels: The starndard text on labels for treatment bottle are prepared and printed electronically, while the trial numbers are manually written on the labels while following the packing list provided by the sponsor.

Location of decoding envelopes and rules for unblinding

SSI, Pharmacovigilance department
The biostatistical unit will prepare one decoding envelope for each participant. The outside front of the envelope will be labelled with the trial code, the Investigator's name, and the participant number, and each envelope will have a paper inside with

similar information and in addition which group the participant is allocated to (active or placebo). The biostatistical unit will give the envelopes to the Safety and Regulatory Advisor at SSI, Pharmacovigilance department. In this way, the Safety and Regulatory Advisor and the doctors at the Pharmacovigilance department can decode the identity of a participant in case of a serious adverse event for a single case.

Location of randomisation lists and emergency envelopes

Placement of key documents	Master randomisation list	Allocation list for statistical analyses	Emergency envelopes	Packing list
Investigator	-	-	X	-
SSI, Pharmacovigilance	-	-	X	-
SSI, Quality Assurance (QA)	X	X	-	-
Pharmacy	X	-	-	X

(All items are generated electronically by QA, who has approved test-versions)

The Investigator and the Safety and Regulatory Advisor and relevant doctors at the Pharmacovigilance department at SSI must only open the envelopes in situations in which, in their opinion, unblinding is essential for the appropriate care of the trial subject. At best, a diagnosis should be made, and a treatment should be started before opening the envelopes. Furthermore, if the trial poses a threat to participants in general, Pharmacovigilance and QA must assess this threat, and if unblinding of the entire trial is necessary, then they must approve the unblinding.

8.1.6 Compliance and drug accountability

Compliance procedures

General considerations

All below compliance procedures will be implemented in the CRFs.

The overall purpose with the visits at the PACC is to achieve treatment compliance, and to administer the treatment in a way that feels safe and comfortable for the participant.

In general, the investigator should make an effort to avoid non-compliance for visit where blood is to be sampled and exhaled NO to be measured at the clinic. To achieve

this, the Investigator must continually check the trial calendar at start of section 9 for overview of visits.

Compliance procedure for treatment

When making appointments for subsequent visits, the investigator should reassure that the participant will comply with all the following visits of the trial, i.e. are not planning to cancel. If, however, the participant is planning to cancel (e.g. due to vacation or accidentally), then the investigator must propose self-administration to the participant, and if approved by the participant, the Investigator should follow the check list in the CRF when handing out treatment products to the participant. This includes the following procedures:

1) Check the expiry date of the participants self-treatment bottle.
2) Settle date and phone numbers for phone call on the day of self-treatment (which takes place after the phone call and one the same day)
3) Write the planned treatment date on the bottle or thermo-can
4) Pack thermo-can with treatment bottle, leaflet, powder, and measuring spoon
5) Inform participant about storage in refrigirator, use of the security letter on flight travels (template in Investigator's file), having diary ready at phone call, treatment takes place before phone call and on the same day, and that FEV1 must be recorded daily.
6) Both the participant's two Piko-1's are checked for sufficient memory capacity, and handed out to the pariticipant.
7) Letter to the participant's travel insurance company handed out/send if applicable (template in Investigator's File).
8) Hand out packed thermo can to the participant

Only the Investigator can assess and take action on safety data reported during the phone call (see section 9.1.3 and 11.2).

Compliance procedure for discontinuation of treatment

If participants should discontinue the investigational treatment (potential drop-out or withdrawal issues), the Investigator should ask the participant to continue with diary, Piko-1 measurements, and visits at the level within the participant's means, but also at a constant level, e.g. weekly. The level should be agreed on, planned, and documented in the CRF. This is particular important for collection of diary pages and shifting of Piko-1 when memory capacity runs out.

The purpose of this is to minimise a reduction in quality and statistical power of the trial (i.e. those who are in the placebo group and starts discontinuing only the treatment will be included in additional analyses as part of the placebo group. In addition, those who are in the active group and starts discontinuing only the treatment will be treated as a special group). Such participants will also still have the advantage of gaining experience from close monitoring of his/hers allergic disease.

SSI will provide the Investigator with assistance in developing and carrying out any individual procedures, if applicable.

Drug accountability

The investigational products must be kept in a safe place during the trial under supervision of the Investigator. The Investigator is responsible for maintaining the inventory.

All used treatment bottles must be stored in their original packages until the completion of the trial. Any bottles used for self-administration must be returned to the PACC by the participant, and similarly stored in their original packages.

The Investigator is to document the investigational product accountability at the PACC at the end of the trial (see Appendix 12). The Investigator must return all used and unused treatment bottles to the Pharmacy Services at KVL, if no other procedure for disposal has been given by the sponsor.

8.1.7 Blinding

Tripplet blinding is achieved through the described blinding of the active and placebo products and though the described procedure of randomisation. The effectiveness of the blinding will be assessed after the first data collection, i.e. 3 weeks after start of the trial. Information from participant's diaries will be used. The diaries will contain questions on whether the participant believes he/she receives the active treatment, "the microorganism", or the placebo treatment, and whether his/hers suspiscion is due to the treatment bottles or their content, the PACC's personal (doctor or clinic nurses), or other. The data manager will tabulate the result of the answers, and check whether the participant's distributions by positive and negative answers are fifty-fifty. If not, appropriate action will be taken depending on the given reason for ineffective blinding. No further assessment of the effectiveness of blinding will be performed during the trial. The assessment is reasonable to perform at that time because an effect of active treatment is not expected before after 2-8 weeks.

8.1.8 Precautions

All participants will receive a medical pass indicating participation in clinical trial 2008, treatment with TSO (placebo not mentioned, because of the blinding), the dose, the name of the responsible investigator, and contact phone numbers in case of serious injury (clinic nurse) or questions on trial authorisations (Study director).

During the trial, adverse events will regularly be assessed. Should any adverse effects due to *Trichuris suis* occur, then medical treatment, which kills larvae of *Trichuris* species, is readily available. There are no major side effects of antihelminth treatment; minor side effects occurring in 0.01 – 1.0 % are mild intestinal pain and diarrhoea (Use e.g. Albendazole, 400 mg. Albendazole is not registered for human use in DK due to repro-toxic side effects in animals - but not humans. Therefore it is advisable to contact Infectious disease departments for prescription. Vermox/Mebendazol (2x100mg for 3 days), however, can be readily prescribed).

If participants by chance should experience adverse events related to allergic disease or due to the investigational treatment, they will be offered appropriate treatment at PACC as applicable depending on the nature of the adverse event. If participants then discontinue the trial or the investigational treatment they will also be followed up – as a minimum by visit(s) at and/or telephone contact by PACC - until appropriate treatment is working.

8.1.9 Concomitant medication

Participants will be asked to use the relief medication for allergic rhinitis and asthma provided by PACC. The type of medicine has been chosen to minimise prolonged action, i.e. any carry-over effects. Relief medication is administered in a stepwise manner depending on persistence and severity of symptoms (see Appendix 7). Use of other relief medication is safe, and will not lead to with-drawal from the trial (the Investigator will try to resolve any such violations of the protocol with the participants before or at the 3-weekly visits at the clinic).

Concomitant treatment with antibiotics is allowed, however, metrodinazole (Flagyl) and cyclosporin may have anti-helminthic effects (i.e. kill *Trichuris suis*). Participants will be asked to contact their physician to ask whether he/she can agree on giving an alternative medication or take this off during the trial. The CRF will be used to document and check this compliance.

Other concomitant medication considered necessary for the volunteer during the trial is allowed and must be recorded in the participant's diary (dosage taken, and name of product).

9 Investigational events

STATUS DATE: *28/1-08*			**(SEE CALENDAR NEXT PAGE)**			**Handling of source data**
**Visit no.**	Scre.	B0	B=birch; G=gras	End visit	Follow-up	(Preprinted in CRF)
**Trial week no.**	-8 to -1	0	14-24	35-45	41-50	CRF
**Volunteer information**	×	-	-	-	-	CRF (verbal+written information given)
**Informed consent form (/check-in)**	-	×	-	-	-	CRF+Investigators File (/hand out diary+Piko1+medical pass)
**In/exclusion criteria**: LFU + sIgE + pricktest (ptt. Journal) + blue scheme	×	×	-	-	-	CRF + insert the clinic's blue scheme in CRF
**Baseline questions**	-	×	-	-	-	CRF (+ further baseline questions from box above and below)
Daily diary data collected<br>- Baseline quest. + 3-w period<br>- 3-w. period<br>- FEV-1 (Piko-1)<br>- Follow-up<br>- Transport-files updated	 -<br>-<br>-<br>-<br>-	 -<br>-<br>-<br>-<br>-	 ×<br>×<br>×<br>-<br>×	 ×<br>×<br>×<br>-<br>×	 -<br>-<br>-<br>×<br>×	} Filled in diary pages, check/write ID.nr on all pages, insert pages in transport-files: see ID.nr on back<br>Record at page1 in transport-file the insertion of diary pages
**Skin prick test**	(×↑)	-	-	×		Attach to CRF 1 print-out of the diameter plot from ptt.journal
**Nitric-oxid measurement (NO)** (U numbers indicates ptt. start-up week in calendar next page. NO is measured at blue and red visits)	-	×	U11-U16: 2 visits<br>U17-U21: 1 visit<br>(Holidays: check options in calendar)	×	-	CRF + attach 1 print-out from Niox-PC
**Sampling of blood**<br>**<u>All ptt.s (100 of 100 pt.s):</u>**<br>Green 9ml tubes► RH, green bike<br>Purple 3ml tubes► KPLL<br>**<u>Grass+birch ptt.</u>**<u>s</u> **<u>(30 of 100 pt.s):</u>**<br>Green 9ml tubes► RH, green bike	 <br>-<br>-<br><br>-	 <br>**9ml (100pt)**<br>**3ml (100pt)**<br><br>**9ml (30pt)**	(Holidays: check options in calendar)<br>**9ml (100pt)** (1 visit)<br>**3ml (100pt)** (1 visit)<br><br>**9ml (30pt)** (1 visit)	 <br>**9ml (100pt)**<br>**3ml (100pt)**<br><br>**9ml (30pt)**	 <br>-<br>-<br><br>-	CRF (record sample). Grass+birch group indicated front page<br><br>- (post-trial analyses: Ig Assay; total histamine)<br>- CRF + insert results in CRF (Differential cell count (3ml)).<br><br>- (immediate cell-stimulation. Post-trial analyses: cytokines)
**Safety data check** (at visit/by phone)<br>FEV1 in diary<br>Asthma score in diary<br>"Intestinal" score in diary<br>Serious AEs in diary	 -<br>-<br>-<br>-	 -<br>-<br>-<br>-	 ×<br>×<br>×<br>×	 ×<br>×<br>×<br>×	 -<br>-<br>-<br>-	CRF: Assess, and if applicable, take action.<br>(Serious Adverse Events: fax to SSI within 72 hours. Read chapter 11 in protocol)
**Investigational treatment**	-	×	×	-	-	CRF
**Monthly source data transport to SSI**	-	-	**SSI**	**SSI**	**SSI**	Download data from Piko-1s, shift transport-files (2 sets)

LFU: Lungefunktionsundersøgelse (FEV1)

		**TRIAL CALENDER 2008** (weekends and possible holidays in red)								**VISITS** Primary recruitment (100-xx ptt.)				Secondary recruitment (xx ptt.)							**EVENTS**
		Mon	Tue	Wed	Thu	Fri	Sat	Sun	**Week**	U11	U12	U13	U14	U15	U16	U17	U18	U19	U20	U21	
	**March**	3	4	5	6	7	8	9	**10**	-	-	-	-	-	-	-	-	-	-	-	Modtagekontrol fredag
		10	11	12	13	14	15	16	**11**	**B0**	-	-	-	-	-	-	-	-	-	-	
		17	18	19	20	21	22	23	**12**	-	**B0**	-	-	-	-	-	-	-	-	-	Modtagekontrol fredag
		24	25	26	27	28	29	30	**13**	-	-	**B0**	-	-	-	-	-	-	-	-	Modtagekontrol fredag
	**April**	31	1	2	3	4	5	6	**14**	**B1**	-	-	**B0**	-	-	-	-	-	-	-	Monitor 2/4, 13.00
		7	8	9	10	11	12	13	**15**	-	**B1**	-	-	**B0**	-	-	-	-	-	-	-
		14	15	16	17	18	19	20	**16**	-	-	**B1**	-	-	**B0**	-	-	-	-	-	-
Birch season		21	22	23	24	25	26	27	**17**	**B2**	-	-	**B1**	-	-	**B0**	-	-	-	-	-
	**May**	28	29	30	1	2	3	4	**18**	-	**B2**	-	-	**B1**	-	-	**B0**	-	-	-	Monitor 30/4, 13.00
		5	6	7	8	9	10	11	**19**	-	-	**B2**	-	-	**B1**	-	-	**B0**	-	-	-
		12	13	14	15	16	17	18	**20**	**B3**	-	-	**B2**	-	-	**B1**	-	-	**B0**	-	-
		19	20	21	22	23	24	25	**21**	-	**B3**	-	-	**B2**	-	-	**B1**	-	-	**B0**	-
	**June**	26	27	28	29	30	31	1	**22**	-	-	**B3**	-	-	**B2**	-	-	**B1**	-	-	Monitor 28/5, 13.00
		2	3	4	5	6	7	8	**23**	**G1**	-	-	**G1**	-	-	**G1**	-	-	**G1**	-	-
		9	10	11	12	13	14	15	**24**	-	**G1/G1**	-	-	**G1/G1**	-	-	**G1/G1**	-	-	**G1/G1**	-
		16	17	18	19	20	21	22	**25**	-	-	**G1/G1**	-	-	**G1/G1**	-	-	**G1/G1**	-	-	-
Grass season		23	24	25	26	27	28	29	**26**	**G2**	-	-	**G2**	-	-	**G2**	-	-	**G2**	-	Monitor 25/6, 13.00
	**July**	30	1	2	3	4	5	6	**27**	-	**G2/G2**	-	-	**G2/G2**	-	-	**G2/G2**	-	-	**G2/G2**	-
		7	8	9	10	11	12	13	**28**	-	-	**G2/G2**	-	-	**G2/G2**	-	-	**G2/G2**	-	-	-
		14	15	16	17	18	19	20	**29**	**G3**	-	-	**G3**	-	-	**G3**	-	-	**G3**	-	-
		21	22	23	24	25	26	27	**30**	-	**G3**	-	-	**G3**	-	-	**G3**	-	-	**G3**	-
	**Aug.**	28	29	30	31	1	2	3	**31**	-	-	**G3**	-	-	**G3**	-	-	**G3**	-	-	-
		4	5	6	7	8	9	10	**32**	**G4**	-	-	**G4**	-	-	**G4**	-	-	**G4**	-	Monitor 6/8, 13.00
		11	12	13	14	15	16	17	**33**	-	**G4**	-	-	**G4**	-	-	**G4**	-	-	**G4**	-
		18	19	20	21	22	23	24	**34**	-	-	**G4**	-	-	**G4**	-	-	**G4**	-	-	-
		25	26	27	28	29	30	31	**35**	**END**	-	-	**G5**	-	-	**G5**	-	-	**G5**	-	-
	**Sept.**	1	2	3	4	5	6	7	**36**	-	**END**	-	-	**G5**	-	-	**G5**	-	-	**G5**	-
		8	9	10	11	12	13	14	**37**	-	-	**END**	-	-	**G5**	-	-	**G5**	-	-	Monitor 11/9, 13.00
		15	16	17	18	19	20	21	**38**	-	-	-	**END**	-	-	**G6**	-	-	**G6**	-	-
		22	23	24	25	26	27	28	**39**	-	-	-	-	**END**	-	-	**G6**	-	-	**G6**	-
	**Oct.**	29	30	1	2	3	4	5	**40**	-	-	-	-	-	**END**	-	-	**G6**	-	-	-
		6	7	8	9	10	11	12	**41**	**F**	-	-	-	-	-	**END**	-	-	**G7**	-	-
		13	14	15	16	17	18	19	**42**	-	**F**	-	-	-	-	-	**END**	-	-	**G7**	-
		20	21	22	23	24	25	26	**43**	-	-	**F**	-	-	-	-	-	**END**	-	-	-
	**Nov.**	27	28	29	30	31	1	2	**44**	-	-	-	**F**	-	-	-	-	-	**END**	-	-
		3	4	5	6	7	8	9	**45**	-	-	-	-	**F**	-	-	-	-	-	**END**	-
		10	11	12	13	14	15	16	**46**	-	-	-	-	-	**F**	-	-	-	-	-	Monitor close-out
		17	18	19	20	21	22	23	**47**	-	-	-	-	-	-	**F**	-	-	-	-	-
		24	25	26	27	28	29	30	**48**	-	-	-	-	-	-	-	**F**	-	-	-	-
	**Dec.**	1	2	3	4	5	6	7	**49**	-	-	-	-	-	-	-	-	**F**	-	-	-
		8	9	10	11	12	13	14	**50**	-	-	-	-	-	-	-	-	-	**F**	-	-
		15	16	17	18	19	20	21	**51**	-	-	-	-	-	-	-	-	-	-	**F**	-

Pollencounts >50/m³/day indicating symptoms occur:
Pollen seasons in 2003-2007 in Copenhagen/Viborg
Pollen season in 2007 in Copenhagen/Viborg

B = Birch visits
G = Grass visits
F = Follow-up visit

Blue visits: Measurement of NO, **but not** sampling of blood
Red visits: Measurement of NO **and** sampling of blood**.** The Investigator chooses **G1/G1** or **G2/G2** depending on participant's visit compliance (e.g. holiday); here **G2/G2 HAS FIRST PRIORITY!!**.

Comment on calendar: "Modtagekontrol": IMP and placebo receival at Investigator's site, to be documented using the form "Modtagekontrol" in Investigator's File.

9.1.1 Details of visits

The below described details of the visits have been guidelines for the CRF form, which have been developed by the Study Director together with the Investigator, clinic nurses, the Monitor, and the Data Manager (statistician) prior to the start of the trial. All these persons have at individual meetings read, commented, and learned the procedures in the CRF while guided by the Study Director.

Repeating of data collection and transport of source data from PACC to SSI:

SSI collects CRFs and source data after completion of every visit no. (B1, B2,..) starting from the last participant's visit B1. To reduce work load while securing source data, the Investigator documents in the CRF the collection of source data, and inserts the source data at the back of the CRF or in transport files:

Diary pages will be collected in separate transport files [ringbind] due the high number of pages.

Piko-1 data will be downloaded to a portable computer and immediately burned on a CD (the particpants 2nd Piko-1 not in use is placed in plastic folder at start of the CRF).

Counting of the content of used packages of relief medication is dobbelt-checked (nurse, monitor, and sponsor), and after that destruction is allowed to save space at the clinic (destruction boxes is ordered from SMOKA, contact info in Investigator's file).

The follwing template lists are available in the Investigator's file:

- Subject screening log (date and time of screening, sex, age, and if applicable the reasons the participant is not eligible for inclusion). Copy Appendix 9.
- Subject identification code list (CPR, personal trial no., initials, and name). Copy Appendix 10.
- Subject enrolment log (can be generated at the end of trial from the subject identification code list). See Appendix 11.
- Product accountability document with records of expiry dates and self-treatments Appendix 12

First visit: Screening (see "Scre." in flow-chart)

- Screen inclusion/exclusion criteria using information from the ptt. journal (VAS for AR, skin prick test, sIgE reactivity, Lung function examination (LFU)).
- Document screening in the subject screening log. Send screening log to Study Director every Friday.

- Give verbal and then written information to potential volunteers (see Appendix 4 and 8).
- Give the consent form (dated and signed by the Investigators) to potential volunteers. The potential volunteer must have more than one day to decide whether to participate and sign the consent form. The time-interval should be evident when comparing the date in the screening log with the date on the consent form.
- Make an appointment for the potential start up visit at least one day after this visit.

Visit B0

- The volunteer must give informed consent prior to initiation of any trial related test or evaluation. After having collected the consent form (dated and signed by the volunteer) and given the participant a copy, the Investigator can initiate the following procedures:
- Start CRF recording by recording the participant's trial number on the first page:

For participants who have grass allergy according to the inclusion criteria, and also have any history of symptoms of birch allergy plus a positive prick test and/or IgE reactivity to birch, choose an ID.nr. from 1 to 30. For participants who are not birch allergic in this way, choose a number between 31 to 100.

- Check in/exclusion criteria from screening visits, and record and insert baseline information in the CRF.
- Record the participant in the subject identification code list (CPR, personal trial no., initials, name, phone no.; address is entered into the clinic's computer).
- Give a Piko-1 pocket flow-meter to the participant, and instruct in use. There are 2 Piko-1 for each participant. They are placed in a plastic chatex at start of the CRF.
- Give a diary to the participant, and instruct in how to fill it in.
- Sample blood (See flow-chart)
- Perform measurements of exhaled nitrogen oxid with the Niox apparatus. Record the results on a pre-printed template and attach as part of the CRF.
- Administer the first treatment drink to the participant as instructed in section 8.1.1.
- Make an appointment for the next visit between 19 and 23 days after the present visit. Reassure that the participant will comply with all the following visits of the trial, i.e. are not planning to cancel (e.g. now planning a vacation).

Visits hereafter and before the end visit

- Use the calendar shown at start of section 9 to decide whether sampling of blood and NO is schedulled for the visit. If so, then perform the sampling. Record sampling in CRF, attach source data to CRF.
- Collect diary pages covering the time since the last visit, check/write ID no. on all pages, record the collection in the CRF, put the diary pages into dedicated transport files [ringbind].

- Shift Piko-1. Each participant has two Piko-1s. The one not in use is placed in the plastic chatex at the beginning of the CRF.
- Assess the safety data (diary recordings of symptom score for asthma, lung function measurements (Piko-1), and adverse events).

  Always judge and record in the CRF the causality for the safety data and for all unexpected adverse events. If the intensity of asthma symptoms and/or lung function is severe, and/or any adverse events are moderate to severe it must evaluated whether to record them in the AE pages of CRFs. Always judge whether immediate reporting to SSI is required. Serious adverse events are subject to immediate reporting to SSI. See section 11.2 and 11.3 for further details.
- If the participant has used the antibiotics metrodinazole (Flagyl) and/or cyclosporin then try to arrange use of other antibiotics if possible (see section 8.1.9).
- Administer the treatment drink to the participant as instructed in section 8.1.1.
- Collect all packages/boxes of used and unused relief medication from the participant, and record the used medication in the CRF.
- Make an appointment for the next visit between 19 and 23 days after the present visit. Reassure that the participant will comply with all the following treatment visits of the trial, i.e. are not planning to cancel (e.g. now planning a vacation). Consider selft-treatment if the particpants cancels, follow the checklist in the CRF.

After end of last patients visit B1: SSI downloads data from Piko-1 to a dedicated computer. Piko-1 software automatically assigns one folder for the particular Piko-1. The folder's name is the serial number of Piko-1, which is also recorded in the CRF by the Investigator. SSI also transports source data to SSI for data management.

End visit

- Sample blood and NO (see flow-chart). Record sampling in CRF, attach source data to CRF.
- Perform a skin prick test to 10 allergens including grass and birch. Attach source data to CRF.
- Assess the safety data (diary recordings of symptom score for asthma, lung function measurements (Piko-1), and adverse events).

  Always judge and record in the CRF the causality for the safety data and for all unexpected adverse events. If the intensity of asthma symptoms and/or lung function is severe, and/or any adverse events are moderate to severe it must evaluated whether to record them in the AE pages of CRFs. Always judge whether immediate reporting to SSI is required. Serious adverse events are subject to immediate reporting to SSI. See section 11.2 and 11.3 for further details.

- Collect diary pages, check/write ID no. on all pages, record the collection in the CRF, put the diary pages into dedicated transport files [ringbind]. Participants are to keep the diary in order to answer questions for the follow-up visit.
- Collect all packages/boxes of used and unused relief medication from the participant, and record the used medication in the CRF.
- Collect Piko-1; neither of the two Piko-1s are to be used further.
- Make an appointment for the follow-up visit 6 weeks (±2 days) after the present visit.
- Finalise the document for the investigational products accountability (dobbelt check with CRFs). Return the unused and used investigational treatment bottles to KVL together with a copy of the document.
- SSI downloads data from Piko-1 to a dedicated computer.
- SSI transports source data to SSI for data management.

Follow-up visit

- Collect follow-up diary pages. If not filled in, ask the participant to do so.
- Evaluate the follow-up period with the participant.
- Assess any need for further treatment of the participant's symptoms of allergies.
- If the participant is not available for the follow-up visit, then a phone call has to be arranged. Use the phone number recorded in the CRF at the end visit.

SSI transports follow-up source data to SSI for data management.

9.1.2 Efficacy assessments

Comprise:

A. Self-reporting in diaries of scores for daily symptoms of allergic rhinitis, scores for weekly quality of life, and of daily use of relief medication for allergic rhinitis and asthma. Symptoms are to be scored in the evening.

B. Airway inflammation measured by the amount of exhaled nitrogen oxid using a Niox apparatus (Entra Medical) at visit indicated in the trial calendar at start of section 9.

C. Daily lung function (PEF and FEV1) measured by participants using the pocket-flowmeter Piko-1.

D. Skin prick test for 10 allergens (grass, birch and 8 others) at visits indicated in the trial calendar at start of section 9.

E. Immunological assessments based on blood sampling at visits indicated in the trial calendar at start of section 9.

9.1.3 Safety assessments

Comprise:

F. Two-weekly assessments of the last two week's diary data on daily asthma symptoms and use of medication for asthma, and adverse events.

G. Two-weekly assessment of the last two week's daily lung function measurements (Piko-1).

9.1.4 Immunological assessments

Immunological assessments are based on laboratory analyses of blood samples taken at visits indicated in the trial calendar at start of section 9. The assessments comprise:

1. Differential blood count by KPLL using their HEM II analyses (code KPL00011)
2. Total histamine in serum (Fluorometric method by Laboratory 2, *PENDING*)
3. Seroconversion for *Trichuris suis* antigen including sIgA, sIgE, sIgG, and sIgG4 (Assay to be developed by Laboratory 1, Phadia)
4. Detection by ImmunoCAP™ of the serum level of sIgE, sIgG, and sIgG4 to allergen extracts of birch, grass, and other relevant allergens.
5. Detection of production of IL-4, IL-5, IL-13, IL-10, and TGFβ from thawed supernatant of leukocytes pre-stimulated with *Trichuris suis* antigen and with allergen ectracts of grass, birch, and perhaps others. (Cytokine assay by Laboratory 2). This analysis will be performed for subsample of participants (30 of 100).

Differential blood count and total histamine (No. 1 and 2) will be analysed within days, seroconversion (No. 3) will analysed after the last blood sample has been drawn from all participants.

The remaining analyses will only be performed if TSO treatment can be claimed to have an effect on allergic rhinitis.

9.1.5 Handling of blood samples

Blood samples will be collected by experienced medical personnel (for schedule see flowchart and trial calendar at start of section 9). All 100 participants will have drawn one 9ml and one 3ml blood sample. In addition, a subsample of 30 grass <u>and</u> birch allergic participants will have a blood sample of 9ml drawn: The CRF front page for each participant will indicate whether the subject is grass <u>and</u> birch allergic, the subject is then assigned an ID.no. from 1 to 30.

All the 9ml samples mentioned above is drawn in 9ml <u>green</u> heparin tubes, and send to Laboratory 2 (RH), while the 3ml samples is drawn in <u>purple</u> 3ml EDTA tubes and send to Laboratory 3 (KPLL).

Lab 1: Phadia (post-trial analyses)
Lab 2: RH **Phone:** ______________
Lab 3: KPLL **Phone:** 3374 4000

BLOOD HANDLING INSTRUCTION

Labelling

Prior to sampling, the clinic nurse labels the sampling tubes as shown below:

1 or 2 green 9ml heparin tubes: *ID.nr 1-100*	*ID.nr 1-30*	1 purple 3ml EDTA tube: *ID.nr 1-100*
**SSI TSO-01** **GRØN100**<br>Subject No:…………………<br>Visit No:…………………….<br>Sample Date:………………	**SSI TSO-01** **GRØN30**<br>Subject No:…………………<br>Visit No:…………………….<br>Sample Date:………………	**Use KPLL's labels. Use CPR.nr. to link to ID.nr.**

Example: Subject no. 3's number is between 1 and 30. No. 3 is about to have one of two 9ml blood samples drawn at visit B0 on March 10, 2008. For this one sample write:

SSI TSO-01 **GRØN30**
Subject No:………***3***……….
Visit No:………..***B0***………
Sample Date:…***10-02-08***….

Laboratory request forms and labels

Before dispatch for transport, the clinic nurse fills in a laboratory request form for each blood sample. KPLL supplies the clinic with their request forms and labes, while the sponsor provides the following forms and labels in 2 boxes:

Label on box	Total content of file:	Each form contains:
Label on box 1:<br>TSO-01, GRØN100, 9ml, RH	300 requests for Lab 1+2:<br>300 labels	1 original + 2 copies
Label on box 2:<br>TSO-01, GRØN30, 9ml, RH	90 requests for Lab 2:<br>90 labels	1 original + 1 copy

For each request form, fill in subject No., initials, CPR, sex, and sampling date. Then tick of the visit no. of sampling, and tick of the relevant request(s) at the bottom:

☐ **GRØN100** To be send to RH. 1x9ml Heparin tube. Request for total histamine and dispatch of plasma to Phadia. RH forewards request to Phadia, and keeps a copy of the request.

☐ **GRØN30** To be send to RH. 1x9ml Heparin tube. Request for cell-stimulation and later (post)-trial cytokine analyses

After filling in request forms for each tube, the forms are send with the tubes to the lab. Remember to take one copy from each form and archive by ID no. in binders:

Name on back of binder:	Total content of binder:
TSO-01, Rekvisitionskopi, ID.nr. 1-50	50 guide cards (ID.nr. "1", "2",…"50")
TSO-01, Rekvisitionskopi, ID.nr. 50-100	50 guide cards (ID.nr."51", "52",…"100")

Storage and transport of blood samples

Within 24 hours: Store at room temp./refrigerator and then send in isolated boxes.
Transport to Lab 2: Green bike delivery. Phone: 70 103 103. Arrival before 12.00h!
Transport to Lab 3: KPLL collects between 11.45h and 13.45h from Investigator OR receives blood samples between 7.30-14.00h.

9.1.6 Laboratory assays

All blood samples (label GRØN100, GRØN30, and LILLA100) will be processed on the same day they arrive, and according to the standard operational procedures of the laboratories.

GRØN100: Laboratory 2 will separate 0.2ml of the 9ml blood and analyse for total histamine using ***Fluorometric method*** (Assay info *PENDING*). The results are posted directly to the Study Director. The remaining 8.8ml of the 9ml blood will be centrifugated, freezed, and send to Laboratory 1 (Phadia) for post-trial analyses (***seroconversion, and ImmunoCAP assay***). Copy of request forms are to be send with the samples (tick off the requested analyses!). The post-trial results from Laboratory 1 (Phadia) are posted directly to the Study Director.

GRØN30: Laboratory 2 will stimulate the blood samples with T.suis antigen, birch and grass allergen, centrifugate, and freeze the supernatant for post-trial analyses (***cytokine assay***). The post-trial results are posted directly to the Study Director.

LILLA100: Laboratory 3 will run the ***HEM II assay***, and the result will be available next day, if the blood was delivered before 14.00h the day before. The results are posted to the Investigator and the Study Director.

9.1.7 Follow-up

Participants will be followed up six weeks post trial (see flowchart). They will be asked to visit the clinic for an evaluation of the follow-up period, and to fill in the follow-up questionnaire in the diary. To increase compliance, the questionnaire will also be send to participants 4 weeks after their end visit, and if forgotten at the visit, the questionnaire will be handed out at the visit. If particpants cannot attend the follow-up visit, a telephone meeting is arranged already at the end visit (phone no. recorded in the CRF).

In the questionnaire, participants score global symptoms (VAS and 4-point scale, see section 12.4), record use of relief medication for allergic rhinitis and asthma and any other medication used, and adverse events. Furthermore, a safety check of the recorded information is done by the Investigator – any serious adverse events are to be reported to SSI within 72 hours using the adverse event page of the CRF.

The Investigator will evaluate the follow-up period together with the participant, and assess any need for further treatment of symptoms of allergies, or appropriate treatment in case of adverse events.

If the trial and follow-up period suggest long-term effects, permissions and funding will be applied for a follow-up study in the next pollen season. That study will focus on more objective measures (e.g. skin prick tests, blood analyses, NO-, and PiKo-1 measurements) because the allocation has been unblinded.

9.1.8 Time schedule and end of trial

First volunteer/first visit is expected to take place in Q1 of 2008, and last volunteer/last visit is expected to take place in Q3-Q4 of 2008. The clinical study report is expected to be finished between the Q4 of 2008 and Q1 of 2009.

The definition of end of trial is when the final trial close-out monitoring report for the investigator's site has been submitted to and approved by the Sponsor and the Investigator. The report should document that all activities required for close-out of the Investigators site are completed, and copies of essential documents are held in the appropriate files. After close-out of the investigator's site, the blinded statistical analyses can begin.

The definition of time of complete unblinding of the trial is when the final close-out monitoring report for the sponsor's site has been submitted and approved by the sponsor.

The Sponsor is responsible for informing the EC and competent authority (DKMA) of trial terminination within 90 days of the last participant's visit, and of early trial termination with 15 days of the decision to terminate early.

10 Ethical aspects

The study will be conducted in accordance with the declaration of Helsinki. The protocol (Danish version) has been approved by the EC.

The volunteers will receive verbal and written information about the trial including details of any potential risks. All volunteers will be informed that they will have the advantage of gaining experience from close monitoring of their allergic disease through diaries, daily lung function measurements, and consultations with the Investigator every 3rd week. Such education is likely to improve their disease control.[40] All volunteers will also be informed that the investigational treatment has no known side effects. In contrast, long-term use of allergy medication and sublingual immune therapy often has side-effects (minimal sedative effects, oral and ear pruritis, mouth edema). Finally, they will be informed that they will receive medication against allergic rhinitis and asthma free of charge. The principle Investigator has at the PACC conducted 12 clinical trials as Investigator for several medical companies including Phadia AB, AstraZeneca ApS, and Alk Abelló ApS. The sponsor's Department of Epidemiology Research has many years experience with handling of personal data, and statistical analyses, and an international reputation for epidemiological research of high quality. The Pharmacy Services at KVL has experience with, and has been approved by the DKMA, to perform packing, and labelling of treatment products for human clinical trials.

Before entering the trial, the volunteers will sign an informed consent form. The EC has been informed and have approved the recruitment procedure.

The total amount of blood needed from each volunteer during the 6 months participation will be 13 to 23 ml which equals 3-5% of a single blood donation (500 ml).

Blood samples will be coded before they are sent for laboratory analyses, and only the Investigator will have access to information that may link laboratory results with personal identification. After laboratory analyses are done, any remaining blood will either be destructed or returned to storage fascilities at SSI as soon as possible and within less than 3 years.

All documents with information on identity (CPR, names, addresses, and contact information) will be kept in a locked locker in a locked office room at PACC and SSI. All other anonymous information on participants (e.g. source data) will be kept in a locked office room at PACC and SSI, and if possible also in a locked locker in the office room.

Experience with patient acceptability for TSO®: The treatment is a natural way to modify inappropriate mucosal immune responses due to lack of stimulation with Helminth parasites. Experience with treatment of IBD patients has shown that they are very accepting of the therapy when they understand the principles supporting this treatment. Patients are relieved to know that they ingest almost microscopic eggs and do not find (see) worms or eggs in the stool. The therapy does not appear to cause side effects or complications. The therapy can be given alone or in conjunction with other standard medications for IBDs.

Preclinical repeat dose toxicity studies in primates and rodents with the investigational TSO® dose and interval have caused no safety concerns. Furthermore, three clinical trials in humans have been performed and have caused no safety concerns.[34-36] The trials were GCP compliant with limitations.

In a phase I trial in USA,[34] 7 adult patients with active Crohn's disease (n=4) and Ulcerativ colitis (n=3) received a single dose of 2500 *Trichuris suis* ova and were followed every 2nd week for 12 weeks (efficacy, physical examination, complete blood count, hepatic enzymes and bilirubin, erythrocyte sedimentation rate and C-reactive protein, and ova and parasite examination of stool samples). Four of the patients then received a 3-weekly 2500 *Trichuris suis* ova dosages and were similarly followed at 3-week intervals for a further 28 weeks. Overall, there were no adverse events, e.g. infection or invasion, reported with use of ova despite the underlying colonic problems in the diseases.

In an open-label phase II trial in USA,[36] 13 male and 16 female adult patients with Crohn's disease received every 3rd week for 24 weeks the dosage 2500 *Trichuris suis* ova. There were no adverse events reported for the use of ova. Subset analyses of the data suggested that patients faired better on immunosuppressive therapy, e.g. prednisone, which is the type of systemic steroid relief medication to be used in the investigational trial. However, whether the author's result was an artefact could not be established.

In a randomised DBPC phase II trial in USA,[35] 32 male and 22 female adult patients with Ulcerative colitis received every 2nd week for 12 weeks the dosage 2500 *Trichuris suis* ova or placebo. The patients were asked about side effects every 2nd week, and were evaluated at entry, 6 weeks, and 12 weeks (efficacy, medial history and physical examination, complete blood count, erythrocyte sedimentation rate, C-reactive protein,

liver profile, stool examination for ova and parasites, bacterial pathogens, and *C. difficile* toxin). There were no adverse events reported for the use of ova.

During the trial adverse events will regularly be assessed and should any adverse effects to *Trichuris suis* occur, then medical anti-helminth treatment (Mebendazole, or Albendazole), which effectively kills larvae of *Trichuris* species, is readily available. There are no major side effects of antihelminth treatment; minor side effects occurring in 0.01 – 1.0 % are mild intestinal pain and diarrhoea. All participants who by chance should experience adverse events due to the treatment or suspected to be due to the treatment, will be followed up by the Investigator until appropriate treatment is working.

10.1 Payment of volunteers and investigational institution

The volunteers are compensated at the end of the trial for their inconveniences with respect to daily lung function measurements and filling in diaries and questionnaires (2 min. per day for 150 DDK/hour). Thus, around 1000 DKK is paid to each volunteer after completion of the trial. Participants who are withdrawn from participation in the trial, or who discontinue only the investigational treatment, will be paid according to no. of days spend on the trial. Furthermore, all participants are compensated for any documented loss of work time at trial visits of up to 15 minutes (150 DKK/hour) and for documented expenses for transport between home/work and the PACC of up to 100 DKK/visit.

Payment is to be done each month with regards to loss of work time and transport compensation, whereas inconvenience compensation is payed at the end of trial when statistical analyses start. Payment is subject to tax duty except for the transport compensation.

The investigational institution, the PACC, has agreed to be compensated for 1500 DKK per participant's total consultation at the clinic, for the extra work load of clinic nurses (salaries of 2 clinic nurses employed for 7 months; ~469,000 DKK). The amount will be transferred to the Clinic's account at a regular interval agreed with the Investigator at start of the trial.

10.2 Budget

A detailed budget signed by the Investigator and sponsors is part of a written agreement.

Administration of the budget will be performed by the administrative secretary at the Department of Epidemiology Research, SSI (Helle Elisabeth Jørgensen).

The investigational products provided by OvaMed GmbH and are not included in the budget.

The trial is to be funded by public and private organisations. At present, the Danish Research Council (FSS) has delegated 1.356.000 DDK to the trial (~50% of the budget) for initiation. For operational expense, FSS has granted 140.000 DKK, and the Maersk foundation and Aase Ejnar Danielsen foundation has granted 80.000 DKK, all in all

amounting to 15% of the operational expenses. The total cost for operational expenses is 1.5 mill. DKK. SSI will fund up to about 50% of the operational expenses - the exact amount will depend on the funding achieved from other organisations. The remaining costs for operational expenses concerns the blood analyses, and here fundraising is ongoing, and will continue throughout and after the trial until fundraised.

11 Adverse events

This section reviews the procedures for recording and reporting of adverse events in the trial. Relevant definitions and terms are listed. Furthermore, the procedures for immediate reporting of serious adverse events to SSI and for expedited reporting to the DKMA and the EC are described.

11.1 Definitions and terms

All definitions are according to Directive 2001/20/EC, ICH E2A/CPMP/ICH/377/95, June 1995).

Adverse event:
Any untoward medical occurrence in a patient or a subject participating in a clinical investigation and receiving a pharmaceutical product, which does not necessarily have a causal relationship with this product.

Adverse reaction or suspected adverse reaction:
All untoward and unintended responses to an investigational product related to any dose administered. The term 'adverse reaction' implies that there is a suspected relationship between the event and the trial product. In practice this means that there is evidence or arguments that suggest a causal relationship, i.e. a relationship cannot be ruled out.

Seriousness criteria:
The reaction/event

– results in death
– is life-threatening
– requires in-patient hospitalisation or prolongation of hospitalisation
– results in persistent or significant disability/incapacity
– is a medically important condition

NOTE: The term 'life-threatening' in the above definition refers to an event during which the patient was at risk of death at the time of the event; it does not refer to an event which hypothetically might have caused death, had it been more severe.

If one individual reaction/event is serious according to the above criteria, the case as a whole is serious.

If a subject dies, the case as a whole must be categorized as fatal. However, where death is considered to be definitely unrelated to the reported reaction/event according to both the investigator and SSI, the individual reaction/event must not be categorized

as being fatal (ICH E2B (M) (CPMP/ICH/287/95, November 2000).

Unexpected adverse reaction:
An adverse reaction, the nature or severity of which is not consistent with the applicable product information (for example the Investigator's Brochure).

Immediate reporting:
The Investigator's rapid reporting of reactions/events to SSI (reporting within 72 hours of the Investigator's first knowledge of the reaction/event).

Expedited reporting:
The Investigator's rapid reporting of reactions/events to the DKMA and/or the EC (reporting within 15 days of the Investigator's first knowledge of the of reactions/events).

11.2 Standard reporting of adverse events

The Investigator is responsible for the recording of adverse reactions/events in the Adverse Event Forms of the CRFs.

The Investigator must assess the nature of adverse events occurring during this clinical trial (and in the 1½ month follow-up period) as follows:

The causal relationship between an adverse event and the treatment with TSO® and placebo must be assessed by the Investigator using the following terms:

– Not related/unlikely related
– Possible
– Probable
– Certain

The intensity of an adverse event must be assessed by the Investigator using the following terms:

– Mild (i.e. easily tolerated)
– Moderate (i.e. sufficient to interfere with daily activities)
– Severe (i.e. sufficient to prevent normal activity)

The outcome of an adverse event must be assessed by the Investigator using the following terms:

– Fatal
– Not yet recovered
– Recovered with sequelae
– Recovered without sequelae
– Unknown

The seriousness of an adverse event, must be assessed by the Investigator by answering the following questions:

– Did the event result in death?
– Has the event been or is the event life-threatening?
– Has the event required inpatient hospitalisation or prolonging of hospitalisation?
– Has the event resulted in significant or persistent disability or incapacity?

11.3 Expedited reporting of serious adverse events

The Investigator is responsible for the immediate reporting to SSI of serious adverse events/reactions.

Furthermore, non-serious adverse events/reactioins if unexpected or of moderate to severe intensity are subject to immediate reporting:

Although not expected, a mild degree of diarrhoea (e.g. 1-7 days within 3 weeks), anal pruritis (1-7 days within 3 weeks), or flatulence (1-7 days within 3 weeks) are examples of adverse events that should only be recorded in the subject diaries. If such event is judged to be more severe, this must be recorded in the CRF (safety check box), and if judged to be a serious adverse event/reaction, it must be recored in the Adverse Event Pages of the CRF, which is immediately faxed to SSI together with the relevant diary pages on symptoms and medication.

The investigator should always exercise medical and scientific judgement in deciding whether immediate reporting is appropriate.

The Investigator should fill in the Adverse Event Pages of the CRF and fax a copy to SSI within 72 hours of his/her first knowledge of a serious adverse reaction/event.

The initial report should be followed up by follow-up reports using the same procedure if additional important information becomes available.

SSI, Pharmacovigilance, will ensure that expedited reporting to the competent authorities and to the EC is done within the required time lines. To shorten response-time from participant to Investigator to SSI, SSI will deliver a medical passport to all participants indicating contact phone no. in case of serious injury (clinic nurse), and in case of questions on authorisations (Study Director).

- For fatal and life-threatening Suspected Unexpected Serious Adverse Reactions (SUSARs) SSI should report to the competent authority and EC as soon as possible and *no later than 7 calender days* after the sponsor has first knowledge of the minimum criteria for expedited reporting. Any relevant report should be communicated within an additional 8 calender days.

- For non fatal and non life-threatening SUSARs, and other safety issues (e.g. clinically important increase in expected serious adverse reactions if any, post-study SUSARs reported by the Investigator (e.g. from the CRF safety check at the follow-up visit), or new events related to the trial or drug product, e.g. from newly completed toxicological studies) must be reported to competent authority and EC as soon as possible and *no*

later than 15 calender days after the sponsor has fist knowledge of the minimum criteria for expedited reporting. Further follow-up information should be given as soon as possible.

SSI can independently enforce early trial termination if unblinding reveals serious adverse reactions.

SSI is responsible for submitting line listings of serious adverse reactions/events to the competent authorities and to the EC (at any time during the study or at the end of the study) as required.

Note: SSI is responsible for informing the EC and competent authority (DKMA) of trial terminination within 90 days of the last participant's visit, and of early trial termination with 15 days of the decision to terminate early.

12 Data management and statistical analysis

12.1 General considerations

The Department of Epidemiology Research, SSI, is responsible for data management and statistical analysis of the data related to the trial.

12.2 Data management

Data are collected in source documents. Source documents included in the data flow is illustrated below. The final database consists of SAS data sets. All SAS data sets will be initiated with an audit trail.

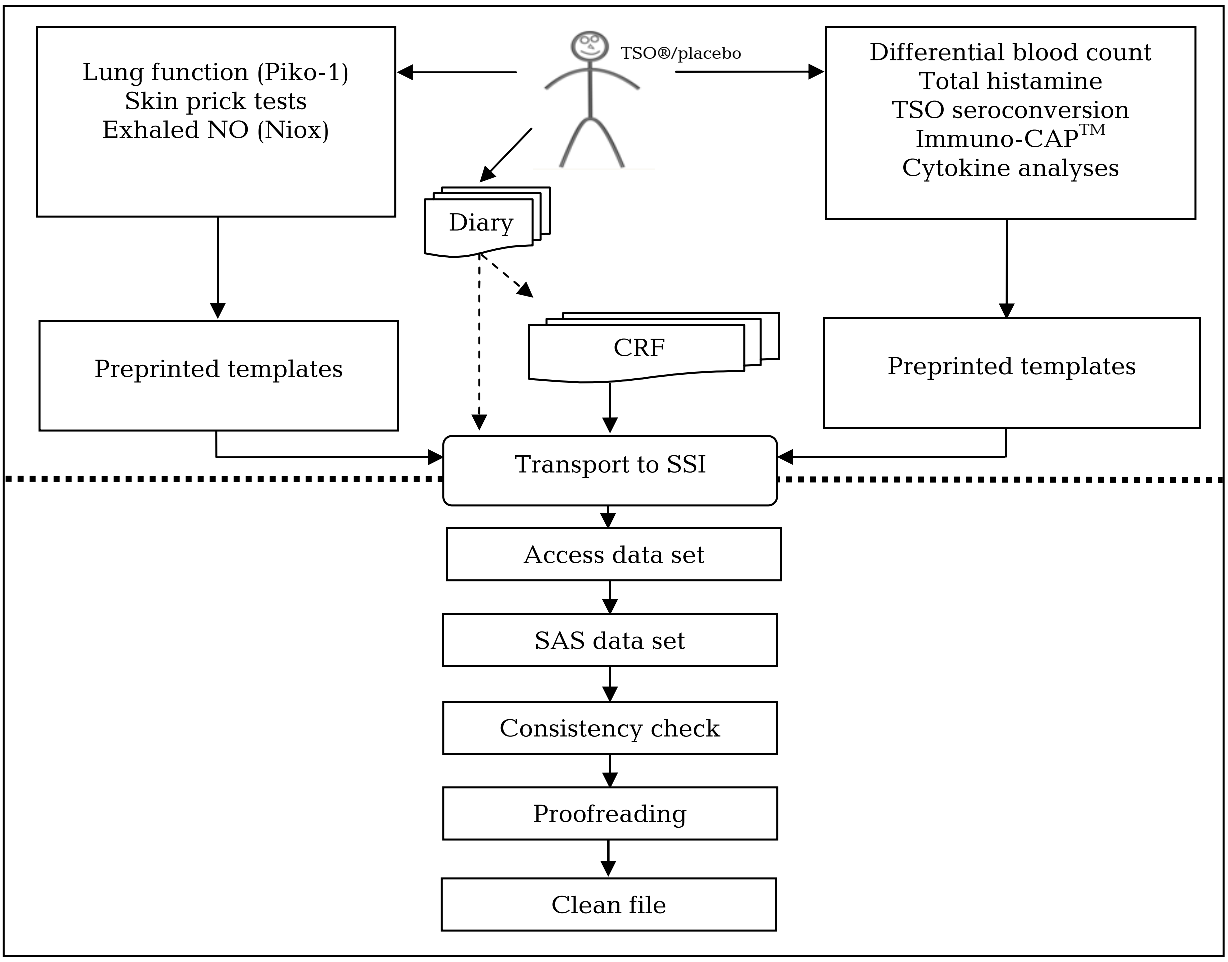

The CRFs and diaries are in Danish.

All source data (CRFs, diary pages, Piko-1, skin prick tests, and NO data) are to be transported from PACC to SSI every month.

Data from participants' Piko-1 devices are to be downloaded directly from the device to a dedicated computer using Piko-1 software. The data will be burned on 1 CD after each download (back-up), and compiled on burned 1 CD at the end of trial.

At SSI, the source data (e.g. CRFs) will then be manually entered into Microsoft Access 2003 data sets and transferred to SAS data sets using Stat/Transfer 8 (Circle Systems). Any electronic source data (CDs) will be transferred directly to SAS data sets using Stat/Transfer 8. Entered diary data on use of relief medication for allergic rhinitis and asthma is run through a custom-made SAS program, which calculates medication scores as described in Appendix 7.

The SAS data sets will then be checked for consistency and plausibility by custom-made SAS programs. Print outs of the SAS data sets will be proof-read against the source documents. All ambiguous or implausible data items will be resolved by potential queries to the Investigator. Data that cannot be corrected (for example diary data not filled in, lung function not measured, or NO-test or skin tests not performed) will be identified as "verified missing".

Immunological data are recorded on preprinted templates by the Laboratories (and if possible then also electronically), and then send to SSI (source documents). The data will then be entered manually or electronically into a SAS data set (single entry). The SAS data set will be checked for consistency and plausibility by custom-made SAS programs. Print outs of the SAS data sets will then be proof-read against the source documents. All ambiguous or implausible data items will be resolved by potential queries to the laboratories. Data that cannot be corrected (for example laboratory test not performed, or implausible outliers as confirmed by the laboratory) will be identified as "verified missing".

All print outs with raw data will be kept in a secure place at the Department of Epidemiology Research, SSI, and kept for the mandatory period of 15 years.

Evaluability criteria

Participants fulfilling the following evaluability critieria will then be electronically marked for later analyses:

- All *intention to treat* participants, i.e those with at least one treatment with TSO or placebo.
- All participants with at least one day of diary data
- All participants with at least one day of lung function data
- All participants with data from visit B0, and at least one subsequent visit from which data on differential blood counts are available.
- All participants with data from visit B0, and at least one subsequent visit from which data on total histamine are available.
- All participants with data from visit B0, and at least one subsequent visit from which data on IgA, IgE, IgG, and IgG4 levels in blood are available. Subjects with information on seroconversion to T.suis is submarked
- All participants with data from visit B0, and at least on subsequent visit from which data on exhaled NO are available.
- All participants with data from visit B0, and at least on subsequent visit from which data on cytokine levels are available (i.e. blood from 30% of participants).
- All participants with available data on skin prick test results from visit B0 and the end visit.

Thus, each marking indicates whether the participant is evaluable for analyses with regards to TSO/placebo treatment and each type of assessment or measurement. This procedure also fascilitates identification of relevant groups to be data cleaned and analysed.

Data from the follow-up visit are not to be used in assessing evaluability criteria.

12.3 Clean file procedures

At the end of the trial, when the data entry from the CRFs and source documents have been validated, the SAS data sets reaches clean file status. Identical copies of the SAS data sets will then be copied to two CDs. One CD will be archived in the trial master file (Sponsor's File) by the Study Director, and the other CD will be archived by the Data Manager, both at the Department of Epidemiology Research, SSI. The statistical analysis reported in the clinical trial report will be based on the clean file data on the clean file CD of the Data Manager.

12.4 Statistical analysis

The primary purpose of the trial is to assess efficacy. The secondary purpose is to assess efficacy and safety. No interim analyses are planned.

After completion of the trial, the Data Manager (statistician) receives the "Allocation list for statistical analyses" from the pharmacist at KVL. The list reveals the two allocation groups for participants, but not who received active or placebo treatment, i.e. the Data Manager is blinded. The Data Manager then performs the statistical analyses in two sets. In the first set, one group is assumed to belong to the active treatment group, and in the second set, the group is assumed to be the placebo group. When all data analyses are done the Data Manager recieves the "Master randomisation list", decodes which is the active and placebo group, and choose to present the set of analyses with the true definition of the groups.

Content validity will be checked and reported for variables where data is available to do so:

- Total daily symptom score for asthma (symptom A+B+C+D) will be checked for correlation with lung function data.
- Reactivity of specific IgE, skin reactivity to corresponding allergens, and symptoms to corresponding allergens will be checked against each other (sensibility and sensitivity analyses).
- Relief medication recorded in diaries will be checked against the Investigators record of returned packages of used and unused relief medication.

Baseline characteristics (see section 7.1) will be categorised according to absolute and relative values in active and place group, and reported in tables.

The definition of endpoints and claims for efficacy and support of efficacy claims are described in section 12.4.1 and 12.4.2. Efficacy for all endpoints will be visualized using curves and plots and quantified using regression techniques allowing for within subject correlation. For dichotomous endpoints, log-linear binomial regression will be used. A p-value of less than 5 % will be considered statistically significant.

Separate analyses will be performed to evaluate the influence of

- the impact of relief medication on relevant outcomes by analysing efficacy and safety for days/weeks where no relief medication is used.
- the impact of seroconversion for *Trichuris suis* infestation on all outcomes by analysing efficacy and safety for subjects who have seroconverted.

— the variation in birch and grass pollen counts by analysing efficacy and safety in periods with high+moderate and low pollen counts according to DMI's categorisation of pollen counts in Copenhagen. If participants have spend days close to the other field station for pollen counting in Denmark (Virum) or field stations abroad, these measures will be used (the participants location will be based on weekly recording in diaries of any days spend outside the Copenhagen area and where. For days spend abroad, max. pollen count, if available for the location, is to be used (recorded by the Investigator in the CRF). The investigator have been advised through the CRF to use www.polleninfo.org for Europe and www.pollen.com for USA pollen surveillance.

— the variation in individually reported pollen triggers of symptoms of allergic rhinitis based on diaries, i.e. weekly recording of triggers (pollen, fur animals, house dust mites, fungis, cold/dust/strong smell, not know), and on what days the triggering took place.

Missing values for a participant will be assigned the average of values in all time corresponding measurement for other participants (e.g. a missing score for symptoms on day 100 will be assigned the average of scores for symptoms in all other participants at day 100). To evaluate the sensitivity of this approach, the two follow analyses will be performed:
- the results of two analyses will be compared; one assigning the best plausible outcome to all missing values in both active and placebo group, and the other assigning the worst possible outcome to all missing values in both active and placebo group.
- the results of two analyses will be compared, one assigning the best possible outcome to missing values in the placebo group, and the worst possible in the active group, and vice-versa.

12.4.1 Primary endpoints

The statistical part of the primary efficacy analyses will be based on diary data, and will be performed on all *intention to treat* participants. Table 1 and 2 summarise the two primary endpoints I and II used in the analyses.

Overall, an efficacy claim can be for at least one of the primary variables I and II.

The efficacy claim for the primary variable I will be a significant score reduction in the mean of total daily symptom score for allergic rhinitis (symptom A+B+C, and/or A+B+C+D, see table 1) between active versus placebo group. In addition, if there is a significant score reduction in the mean of total daily scores for eye symptoms (symptom E+F, see table 1) between active versus placebo group, then it will reported that the efficacy claim for variable I is supported.

The efficacy claim for the primary efficacy variable II will be a significant reduction in number of well-days between the average number of well-days in active versus placebo group. The efficacy claim can be for at least one of the two definitions of well-days described in table 2.

Table 1. Diary variables for primary endpoint I.

**Assessement**	**Interest**	**Range**
Total daily score for relevant seasonal nasal symptoms of allergic rhinitis*: The sum of the symptom components A+B+C described below.	A significant score reduction between average score in active vs. placebo group.**	0-9**
Total daily score for all nasal symptoms*: The sum of the symptom components A+B+C+D described below.	A significant score reduction between average score in active vs. placebo group.	0-12
Total daily score for eye symptoms*: The sum of the symptom components E+F described below.	A significant score reduction between average score in active vs. placebo group.	0-6
**Components of the primary endpoint variable I**		
Daily score for one symptom of allergic rhinitis*:<br>A: runny nose	A significant score reduction between average score in active vs. placebo group.	0-3
Daily score for one symptom of allergic rhinitis*:<br>B: sneezing	A significant score reduction between average score in active vs. placebo group.	0-3
Daily score for one symptom of allergic rhinitis*:<br>C: itchy nose	A significant score reduction between average score in active vs. placebo group.	0-3
Daily score for one symptom of allergic rhinitis*:<br>D: blocked nose	A significant score reduction between average score in active vs. placebo group.	0-3
Daily score for one symptom of allergic rhinitis*:<br>E: gritty feeling/red/itchy eyes	A significant score reduction between average score in active vs. placebo group.	0-3
Daily score for one symptom of allergic rhinitis*:<br>F: watery eyes	A significant score reduction between average score in active vs. placebo group.	0-3

**Four point scoring of severity of each symptom: 0 = no symptoms, 1 = slight symptoms, 2 = moderate symptoms, 3 = severe symptoms.

**Power calculation: The primary effect measure of average total daily nasal symptom score (symptom A+B+C) can reach a daily maximum of 9, and is around 2-4 among pollen allergic adults in the pollen season. With 50 active and 50 placebo participants we will be able to detect with 80% power and at a 5% significance level, a significant score reduction of at least 1.2 between active group and placebo group, e.g. with an average score of usually 4 in the peak of the pollen season a reduction of 30% can be detected. This basic power calculation satisfies EMEA guidelines for an explorative phase II trial on allergic rhinitis (CHMP/EW/2455/02, oct 2004).

Table 2. Diary variables for primary endpoint II.

**Assessement**	**Interest**	**Range**
Number of well-days for allergic rhinitis<br>Days with all symptom scores for allergic rhinitis of ≤ 2 and no use of relief medication for allergic rhinitis (score =0)<br>The 2 components of the variable are described in table 1 and 3.	A significant reduction between average number of well days in active vs. placebo group.	0 to 6 months
Number of well-days for allergic rhinitis and asthma.<br>Days with all symptom scores for allergic rhinitis and asthma of ≤ 2 and no use of relief medication for the diseases (score =0)<br>The 4 components of the variable are described in table 1, 3, and 4.	A significant reduction between average number of well days in active vs. placebo group.	0 to 6 months

12.4.2 Secondary endpoints

The statistical part of the secondary efficacy analyses will be based on self-reported disease activities related to allergic rhinitis and asthma (diary data), clinical skin and airway activity, and immunological activity, while the secondary safety analyses will be based on particular disease activities related to asthma (diary data) and clinical airway activity (lung function data), as well as adverse events. Table 3-6 summarises the secondary endpoint variables.

All secondary analyses will be performed on *intention to treat* participants.

If there is significant positive effect for one or more of the secondary endpoint variables, it will be reported that the effect is supportive of a primary effcacy claim, but the effect will not alone be used to claim efficacy.

Presentation of adverse events

The number of participants and the number of adverse events in the active and placebo group will be tabulated by causality (certain, probable related, possible related, not related/unlikely related) and by severity (mild, moderate, severe), and reported in this form.

Furthermore, the number of participants, if any, with events assessed by the investigator as treatment-emergent, and with events resulting in withdrawals, will be reported and discussed in the report.

Finally, if there are >5% of participants with treatment-emergent adverse events reported by the investigator, the number of participants in active and placebo group will be tabulated by the type of adverse events.

Table 3. Diary variables for secondary endpoints.

Assessement	Interest	Range
Total daily score for use of relief medication for allergic rhinitis calculated on the basis of recorded use of medication for allergic rhinitis. See Appendix 7 for scoring system.	A significant score reduction between average score in active vs. placebo group.	0-38 (for guidance)
Daily/weekly assessment of severity of all symptoms of allergic rhinitis on a 100 mm visual analog scale (VAS). 0 mm = not troublesome 100 mm = worst thinkable troublesome.	A significant score reduction between average mm in active vs. placebo group.	0-100 mm
Assessment at the end of the trial of all symptoms of allergic rhinitis during the trial when compared with symptoms during the previous pollen season. 5 categories: Much better, better, the same, worse, much worse.	A significant relative reduction in percentages of active vs. placebo group. Percentages are calculated for each category	5 categories
As above, but dichotomous: Improved (much better, better); Not improved (the same, worse, much worse)	A significant relative improvement for active vs. placebo group.	Dicho-tomous.
Weekly score for Rhinitis Quality of Life (mini RQLS) developed and validated by E. F. Juniper (Clin Exp Allergy 2000; 30(1):132-140). Calculated as the average of the scores (0-6) for each of 14 quality of life items: 3 activities, 2 practical problems, 3 nose symptoms, 3 eye symptoms, and 3 other symptoms.	A significant score reduction between average score in active vs. placebo group.	0-6
Total daily score for use of relief medication for asthma calculated on the basis of recorded use of medication for asthma. See Appendix 7 for scoring system.	A significant score reduction between average score in active vs. placebo group.	0-32 (for guidance)
Daily recording of severity of diarrhoea, anal pruritis, flatulence.<br>Severity is recorded for each event as mild (i.e. easily tolerated), moderate (i.e. sufficient to interfere with daily activities), or severe (i.e. sufficient to prevent normal activity).	**SAFETY:** All adverse events	(No previous data)
Total daily score for all symptoms of asthma*: A+B+C+D described below.	A significant score reduction between average score in active vs. placebo group.<br>**SAFETY**: A participant's score must not be >7 points for >60% of 14 days. History of asthma at inclusion will be taken into account.	0-12
**Components of the secondary endpoint variable for asthma symptoms (box row above)**		
Daily score for one symptom of asthma*:<br>A: cough	A significant score reduction between average score in active vs. placebo group.	0-3
Daily score for one symptom of asthma*:<br>B: wheeze	A significant score reduction between average score in active vs. placebo group.	0-3
Daily score for one symptom of asthma*:<br>C: chest tightness (dyspnoea)	A significant score reduction between average score in active vs. placebo group.	0-3
Daily score for one symptom of asthma*:<br>D: exercise induced symptoms	A significant score reduction between average score in active vs. placebo group.	0-3

* Four point scoring of severity of each symptom: 0 = no symptoms, 1 = slight symptoms, 2 = moderate symptoms, 3 = severe symptoms.

Table 4. Skin and airway activity variables for secondary endpoints

Measurement method	Variables	Units	Interest	Ranges/ comments
Forced Expiratory Volume 1 (FEV1) measured by participants using a Piko-1 pocket flowmeter	Lung function.<br>Average FEV1 during the trial.	liter (l)	A significant relative increase in average FEV1 between active and placebo group.<br>**SAFETY**: Only participants with FEV1 above 70% of expected are included in the trial. A change of more than 20% for any participant when compared with his/hers baseline, will be evaluated by the Investigator, and appropriate action taken (e.g. steroid treatment, or discontinuation of trial or investigational treatment)<br>Guidelines for severity:[41]<br>Mild: >70%<br>Moderate: 60-69%<br>Moderately severe: 50-59%<br>Severe: 35-49%<br>Very severe: <35%	Measured daily; used for safety checks by investigator every 3rd week.<br>FEV1: maximum volume of air expired in 1 second from full inspiration<br>Expected/normal: Ages 18-65: Males 1.5-5.6 liter, females 1.1-4.0 liter<br>Must be related to height, age, sex, ethnicity (black/white).<br>Two-point short-term changes of >12% and 0.2 l in the FEV1 are usually statistically significant and may be clinically important.[41]
Peak Expiratory Flow (PEF) measured by participants using a Piko-1 pocket flowmeter.	Lung function<br>Average PEF during the trial.	l/min	A significant relative increase in PEF between active and placebo group.<br>No particular emphasis will be put on the results, i.e. they will not be used to support a primary efficacy claim. PEF is not a reliable measure of lung function.[41] However, the data are automatically generated by Piko-1.	Measured daily<br>PEF: maximum flow rate that can be generated during a forced expiratory manoeuvre.<br>Expected/normal: Ages 18-65: Males 420-700 l/min, females 250-520 l/min.<br>Must be related to height, age, sex, ethnicity (black/white).
Niox apparatus (Entra medical). Exhaled Nitrogen Oxid (NO) is measured	Average NO exhaled during the trial.	ppbv*	A significant relative reduction in NO in the active vs. placebo group.	Measurement at visit B0, (1 Birch visit for most subjects), 1 Grass visit, and the end visit<br>Normal: 15-20 ppbv<br>Pathological ranges: 50-100 parts/billion volume (ppbv)
Diameter of wheal reaction on the volar surface of the forearm, to extracts of grass, birch, house dust mites (*D. farinae*, *D. pteronyssinus*), mugwort, cat, dog, horse, *Alternaria*, and *Cladosporium*. Histamine and saline control.	Skin prick test.<br>Reactivity (≥3 mm) or not (dichotomous)	mm	A significant relative reduction in skin reactive participants in active vs. placebo group.<br>Calculated for each allergen and for all allergens.	Tested or reported at visit B0, and tested at end visit.<br>Normal: <3 mm.<br>A wheal diameter ≥3mm, is considered clinical significant, i.e. is associated with increased risk of allergic rhinitis.

Table 5. Immunological activity variables for secondary endpoints

Method/ Assay	Vari -able	Units	Interest	Ranges/comments
ImmunoCAP^{TM}<br><br>(single allergen)	IgE,<br>IgG,<br>IgG4	See comments	A relative reduction in the average concentration [ ] in active vs. placebo group for sIgE, sIgG, sIgG4, and sIgG/sIgG4 ratios to each allergen extract of birch, grass, and other relevant allergens measured in supernatant.<br><br>sIgE class $\geq$2; [sIgE] $\geq$0.7 kU$_A$/l will be considered clinical significant.<br><br>An increase in sIgG/sIgG4 ratio is of interest but no domatic interpretation exist. Increased ratios are generally seen during immune therapy for allergies indicating immune deviation or development of tolerance.	Blood for analyses is sampled at visit B0, 1 Grass visit, and the end visit.<br><br>Measuring ranges for:<br><br>[sIgE]:0.35-100 kU$_A$/l (normal: $\leq$0.35 kU$_A$/l]<br><br>[sIgG]: 6-86 mg$_A$/l (perhaps normal: $\leq$50 mg$_A$/l; i.e. highest concentration observed among 25 non-atopic donors. Source: Phadia AB)<br><br>[sIgG4]: 0.16-19.7 mg$_A$/l (perhaps normal: $\leq$5 mg$_A$/l; i.e. highest mean concentration reported in a study of 100 non-allergic donors. Source: Phadia AB).
ImmunoCAP^{TM} (single antigen)	IgE,<br>IgG,<br>IgG4,<br>IgA	kU$_A$/l or mg$_A$/l<br><br>(see above)	Relative change to baseline of sIgE, sIgG, sIgG4, and IgA to extract of *T. suis* antigen measured as the concentration in supernatant.<br><br>Seroconversion level will be determined, if possible, and is of greatest interest.	Blood for analyses is sampled at visit B0, 1 Grass visit, and the end visit<br><br>*T. suis* antigen is provided by KVL (Helene Kringel), and details of assay will be developed by Phadia AB.
HEM II (differential blood count)	 <br><br><br>Bas<br>Eo<br>Hæm<br><br>Leu<br>Lym<br>MCV<br>Mono<br>Neu<br>Ery<br><br>Thro<br>Uncl.	 <br><br><br>10^9/l<br>10^9/l<br>mmol/l (F)<br>mmol/l (M)<br>10^9/l<br>10^9/l<br>10^{15}/l<br>10^9/l<br>10^9/l<br>10^{12}/l (F)<br>10^{12}/l (M)<br>10^9/l<br>10^9/l	Relative change to baseline of each differential count. The number of values below/above detection limits will be evaluated. Interest are:<br>**<u>Detection</u>** — **<u>Interest/Pathology (see KPLL)</u>**<br>Bas: no info<br>Eos: 14.90 — Parasit, Allgy, steroid▼<br>Hæm: 4.1 — Anemia▼ dehydration▲<br>(same) — (same)<br>Leu: 0.5 ; 100.0 — Infection/inflam./cancer/steroid▲<br>Lym: no info — no info<br>MCV: no info — Anemia ▲(folate) ▼ (Fe)<br>Mono: no info — no info<br>Neu: 0.51 — no info<br>Ery: no info — dehydration▲<br>(same)<br>Thro: 31 — chronic inflam.▲<br>Uncl.: no info — no info	Blood for analyses is sampled at visit B0, 1 Grass visit, and the end visit.<br>Normal, 18-65 years, (KPLL)<br><br>Basophils $\leq$0.20<br>Eosinophils $\leq$0.45<br>Hæmogl. (Fe) 7.0-10.0F<br>8.0-11.0M<br>Leucocytes 3.5-9.0<br>Lymphocytes 3.0-9.0<br>MCV (B-erytro.) 80-100<br>Monocytes $\leq$1.10<br>Neutrophils 1.80-7.40<br>Erythrocytes 3.70-5.50F<br>4.10-6.10M<br>Thrombocytes 150-400<br>Unclassified $\leq$0.60
Fluorometric method (PENDING)	total hista mine	ng/ml	Relative change to baseline of the serum level. Lower detection limit: 47ng/ml. No upper detection limit.	No established normal level
Assay for cell-stimulation, cytokine production, and cytokine RT-PCR	IL-10<br>IL-4<br>IL-5<br>IL-13<br>TGFβ<br>IFNg	*PENDING*	Relative change to baseline of grass, birch, mite, and *T.suis* antigen stimulated IL-10, IL-4, IL-5, IL-13, TGFβ, and INFg production/expression measured as concentration in supernatant from PBMC.	Blood for analyses is sampled at visit B0, 1 Grass visit, and the end visit<br><br>*T. suis* antigen is provided by KVL. (Helene Kringel)

13 Good Clinical Practice considerations

13.1 Declaration of Helsinki

This clinical trial will be conducted in accordance with the principles of the Declaration of Helsinki (see Appendix 2 for the latest revision of the Declaration of Helsinki).

13.2 Subject information and informed consent

Before entering the trial, the volunteers will receive verbal (Appendix 8) and written (Appendix 4) information about the trial, including details of any potential risks. The volunteers will sign an informed consent form before entering the trial (Appendix 3).

13.3 Ethics committee

The EC has approved the trial based on a Danish version of the protocol, which is compatible with the present protocol (j.no. E-KF-01 2006-4100). The address of the EC is:

De videnskabsetiske komitteer for København og Frederiksbergs kommuner
Københavns Kommune
Sundhedsforvaltningen
Postboks 620
Sjællandsgade 40
2200 København N
Tlf. +45 3530 3530 +45 3530 3402; +45 3530 3407; +45 3530 3409; +45 3530 3405
E-mail: videmail.suf@ipost.kk.dk

The sponsor has reassured the composition of the EC followed the ICH6 guideline. Thus the EC consisted of more than 5 persons, at least 1 member whose primary area of interest was non-scientific, and at least 2 members were independent of the Investigator's and Sponsor's institution: The regional committee was no. 2, and consisted of Jens Bülow (MD), Edith Holm (nurse teacher), Majken Johansen (lawyer), Gitte Kronborg (MD), Henrik Off (layman), Manja Sand (layman), and Kjeld Schmigelow (MD).

The EC has been notified about an amendment of the participant information on November 2, 2007 (notification no. 16971), and has approved the amendment on December 7, 2007. Appendix 4 contains the approved version (version 2).

Dated (version number), documented approval from the EC will be located in the Sponsor's and Investigator's File. All correspondence associated with ethical approval is filed by the Study Director.

13.4 Health authority submission and approval

Version no. 1 of the protocol has been submitted to the DKMA on November 1, 2007, and evaluated by the DKMA (j.nr. 2612-3616), who on December 21, 2007, approved commencement of the trial on the condition that an amendment protocol was submitted (as well as results of trial batch analyses). The present version 2 of the protocol is the amendment protocol. The protocol takes into account the substantial amendment required by the Agency: A change from 2-weekly doses to 3-weekly doses in maximum 6 months, and that women of childbearing potential cannot be included in the trial.

The DKMA requested that the protocol be submitted before January 28, 2008.

Any permissions regarding custom during transport of the manufactured investigational products from Ovamed in Germany to the Pharmacy in Denmark is granted automatically if the DKMA approves the study.

For participants who choose to travel abroad by airport (despite the use of inclusion criteria to avoid travels abroad), a security and custom letter will be handed out. The letter has been approved by the relevant Copenhagen Police office (henrik.kristiansen@cph.dk).

The address of the DKMA is:

Lægemiddelstyrelsen
Kliniske forsøg, Inspektionen
Attn.: Batch no. for clinical testing material
Axel Heides Gade 1
DK-2300 Copenhagen S

If the DKMA during the trial have reason to assume, that the trial is not conducted according to the approval, or if there is any other circumstances giving doubt concerning safety and scientific aspects of the trial, the DKMA can demand the trial to be changed or temporarily stopped, or the DKMA can forbit that the trial continues. Before the DKMA takes such decisions, the DKMA can request a statement from SSI or the Investigator to be submitted within 7 days. If the DKMA decides to stop or forbit the trial, the DKMA will immediately report its decision to the EC, EMEA, EU commission, and the other EU countries.

13.5 Subject data protection

The Investigator is responsible for keeping a subject identification code list of all participants in the trial.

SSI will receive person identification information in order to administer payment of the participant during and after the trial (CPR, name, address, phone number, bank account no., and tax level). All data management at SSI is done so that data entering during trial and post-trial data management are performed separately by the data manager, Study Director, and research nurse using anonymised data, while the administration of the payment of participants by the secretary is done separately using person identification data.

All person identification data will be kept in a locked locker in a locked office room at the PACC and SSI.

The volunteers will be informed that the results will be stored and analysed by computer and that confidentiality will be secured.

The volunteers will also be informed, both verbally and in writing that authorised persons, both from the Sponsor (e.g. the Monitor) and from Danish or foreign regulatory authorities (e.g. inspectors), together with the Investigator may review medical patient records relevant for the trial.

The Data Protection Agency has approved the trial (J.nr. 2006-54-2215, see Attachment 10.03)

13.6 Investigators responsibilities

The Investigator is responsible for the conduct of this clinical trial in accordance with the protocol, the current guidelines for Good Clinical Practice (GCP), and any relevant Danish regulations. He undertakes this responsibility by signing the Investigator's Statement Form (Appendix 5) and the full written agreement.

The form and agreement must be signed before the first subject is included in the trial.

The Investigator is, moreover, responsible for the proper reporting of any adverse events according to the procedures described in the Adverse Events Section of this Protocol.

In case of early trial termination or suspension, the Investigator should promptly inform all participants, assure appropriate therapy, and follow-up for participants.

If the Investigator delegates his responsibilities to another person, this must be approved by SSI and confirmed in writing.

The Investigator is responsible for informing volunteers about the trial and for keeping the subject identification code list.

The principle Investigator has at the PACC conducted 12 clinical trials as Investigator for several medical companies including Phadia AB, AstraZeneca ApS, and Alk Abelló ApS. All trials have concerned immune therapy for allergic diseases. The PACC is Denmark's largest clinic with a daily number of patient visits of up to 100.

13.7 CV and log of staff

Before initiation of the trial, current signed and dated curricula vitae for the Investigator and the co-Investigator must be collected, and approved and signed by the Study Director. Personnel involved in the trial at the PACC, SSI, KVL, must be listed in log of staff. For the Study Monitor, Data Manager, and other persons who are allowed to make entries in the CRFs or in other trial related documents, signatures and initials must be listed. The log(s) must be kept in the Investigators' Files, except for the listings of Data Managers, which will be kept in the Sponsor's file (see Appendix 13).

13.8 Training

The Principal Investigator is responsible for the adequate training of the co-Investigator and other study personnel involved at PACC. SSI will provide the Principal Investigator, the co-Investigator and other study personnel with relevant instructions and information needed for the conduct of this clinical trial in accordance with the current GCP guidelines and this protocol. The information and training will be given prior to the start of the trial. Certificates and CVs for qualifications of each attendee will be kept in the Investigators' Files and copies in the TMF at SSI (Sponsors file).

13.9 Monitoring

The clinical Trial Monitor from the GCP unit at Copenhagen University will monitor the PACC at start (i.e. initiation of Investigators site, expected February 21, 2008), every month, and after the last visit (last "end visit").

Furthermore, the monitor will perform an initiation (February 21, 2008), and close-out visit at the sponsor's site. Any queries should be discussed and resolved with the Investigator and Sponsor.

13.10 Audit and Inspection

The Investigator must give access to personnel from SSI for the conduct of audits. Regulatory authorities, national as well as foreign, must at all times be allowed access to conduct inspections as well. Auditors and inspectors must have access to all trial related documents, including the Investigator's File and the subjects' personal medical records.

13.11 Definition of source data

Informed consent, CRFs, diaries, and documents containing results of skin prick tests, NO measurements, lung function data, laboratory analyses, and adverse drug reporting forms are source documents. Any source data too extensive to bee transferred in writing to the CRF pages are instead inserted at the back of the CRF file as one original. The insertion/collection of the data is recorded in the CRF pages.

All source documents must be kept by the Investigator as part of the Investigators file for a period of 15 years after the completion of the trial. Here, relevant copies of source data originals will be certified post-trial by the sponsor, and handed over to the Investigator for archiving, if this relevant for these explorative phase II data.

13.12 Archiving

It is the responsibility of the Investigator and the sponsor, SSI, to maintain the essential documents as described in the ICH guidelines for at least 15 years after the termination of the trial.

The Investigator is responsible for archiving (at least) the following documents:

– Signed Informed Consent and Confidentiality of Data forms.
– Subject identification code list
– Subject Screening log
– Investigator's copy of the CRFs.
– Completed reporting forms of Serious Adverse Events
– Certified copies of relevant source documents besides from the CRFs. The relevance of source data copies to be archived by the Investigator will be decided post-trial.

The completeness of the Investigator's File should be documented by SSI as part of the termination monitoring visit.

14 Agreement and financial settlement

All written agreements of the trial, listed below, will come into force when all are signed.

The agreements below clearly state the rights and obligations of the parties concerned and include a detailed financial settlement where relevant.

1. Agreement between SSI and Pulmonology and Allergy Clinic Copenhagen regarding clinical trial TSO-01. (Includes financial settlements. In process)

2. Agreement between SSI and the GCP-unit of Copenhagen University Hospital. (Includes financial settlements. The agreement is signed).

3. Clinical Trial Supply and Collaboration agreement between SSI and Ovamed (no financial settlements - free trial supply. Indemnity towards participants included, and covers also the manufacture of rawmaterial by Parasite Technologies. The agreement is signed).

4. Clinical Trial Supply and Collaboration agreement between SSI and Pharmacy Services. (Includes financial settlements. In process)

5. Clinical Trial Blood analyses agreement between SSI and the Allergy clinic, National University Hospital. (Includes financial settlements. In process).

6. Clinical Trial Blood analyses agreement between SSI and Phadia AB. (Includes financial settlements. In process).

15 Insurance

The Investigator, PACC, carries a liability insurance. The policy covers claims arising from injury/injuries caused by accident or neglect in the investigator's supervision and conduct of clinical trials.

The Sponsor, SSI, carries a liability insurance under a worldwide liability programme written by the New Hampshire Insurance Company, through insurance brokers Marsh A/S, Teknikerbyen 25, DK-2830 Virum, Denmark, as part of the worldwide Marsh

insurance broker group. The policy covers claims arising from injury/injuries caused by accidents or neglect in initiation and leading of clinical trials.

The Pharmacy Services at KVL carries a product liability insurance under the State. The policy covers claims arising from injury/injuries caused by accidence or neglect in randomisation, blinding, packing, and labelling of clinical trial materials for human use.

The manufacturer, Ovamed GmbH, carries a normal manufacture insurance, and will by written agreement pay the clinical trial insurance that SSI chooses. The policy will cover claims arising from injury/injuries caused by accidents or neglect in the manufacture of the Investigational Medicinal Product.

Prior to the inclusion of the first subject in the clinical trial, SSI will present a signed Indemnity Statement to the Investigators (see Appendix 6).

16 Confidentiality and disclosure

All CRFs, information and results generated by SSI are considered confidential and shall remain the sole property of SSI.

An internal clinical trial report will be prepared by SSI in co-operation with the Principal Investigator. An abstract of the report will be submitted to the DKMA unless otherwise required. The abstract will e.g. include the number of treated patients, administered dose, duration of dosing, and achieved results and any observed side effects.

No data from the clinical trial may be published, presented or communicated, except to regulatory authorities, prior to the release of the internal clinical trial report, unless approved by SSI in writing. The Investigator agrees not to discuss externally or publish any result from the trial without the possibility of SSI to give comments for up to 60 days after receipt of the manuscript.

In the event of a publication, the names of the authors and their order of appearance will be as follows:

Peter Bager, John Arnved, Steen Rønborg, Tine Westergaard, Jan Wohlfahrt, Jytte Bjerregaard, Stig Thamsborg, Allan Roepstorff, Christian Kapel, Mads Melbye

This is not to exclude a priori from the list of authors other collaborators (like laboratory researchers, technicians, research nurses, statisticians) who made a special contribution to the success of the project. Such an addition of authorship to the publication will be considered on the basis of published standards of authorships. If added, such an author will change neither the first nor the last two author places named in the list above.

17 Changes to the protocol

The clinical trial procedures may be changed if the Principal Investigator and the Study Director agree to the changes. If the changes are substantial, the EC and DKMA must

approve the changes before they can be implemented. All substantial changes must be documented by protocol amendments and rewritten full protocols, if applicable.

Substantial changes include changes with an effect on the safety of participants, the interpretation of the scientific documentation which the termination of the trial is based on, the implementation or conduct of the trial, or the quality or safety of the investigational products.

If the SSI or the Investigator becomes aware of circumstances, which results in a risk for the participant's safety, then SSI or the Investigator must immediately take the necessary precautions for protection of the participant. SSI must immediately inform the DKMA about these new changes, and the precautions taken.

18 References

Reference List

(1) Schubert C. The worm has turned. Nat Med 2004; 10(12):1271-1272.

(2) Wickelgren I. Immunotherapy. Wielding worms at asthma and autoimmunity. Science 2004; 305(5681):171.

(3) van den Biggelaar AH, van RR, Rodrigues LC, Lell B, Deelder AM, Kremsner PG et al. Decreased atopy in children infected with Schistosoma haematobium: a role for parasite-induced interleukin-10. Lancet 2000; 356(9243):1723-1727.

(4) Leonardi-Bee J, Pritchard D, Britton J. Asthma and current intestinal parasite infection: systematic review and meta-analysis. Am J Respir Crit Care Med 2006; 174(5):514-523.

(5) Cooper PJ, Chico ME, Vaca MG, Moncayo AL, Bland JM, Mafla E et al. Effect of albendazole treatments on the prevalence of atopy in children living in communities endemic for geohelminth parasites: a cluster-randomised trial. Lancet 2006; 367(9522):1598-1603.

(6) Yazdanbakhsh M, Kremsner PG, van Ree R. Allergy, parasites, and the hygiene hypothesis. Science 2002; 296(5567):490-494.

(7) Yazdanbakhsh M, Matricardi PM. Parasites and the hygiene hypothesis: regulating the immune system? Clin Rev Allergy Immunol 2004; 26(1):15-24.

(8) Weinstock JV, Summers RW, Elliott DE. Role of helminths in regulating mucosal inflammation. Springer Semin Immunopathol 2005; 27(2):249-271.

(9) Maizels RM, Balic A, Gomez-Escobar N, Nair M, Taylor MD, Allen JE. Helminth parasites--masters of regulation. Immunol Rev 2004; 201:89-116.

(10) Strachan DP. Family size, infection and atopy: the first decade of the "hygiene hypothesis". Thorax 2000; 55 Suppl 1:S2-10.

(11) Aas K, Åberg N, Bachert C, Bergmann R, Bergmann R, Bonini S et al. Epidemiology: Prevalence of allergic diseases. In: van Moerbeke D, editor. European allergy white paper - allergic diseases as a public health problem in Europe. Bruxelles: UCB Pharmaceutical Sector Braine-l'Alleud - Belgium; 1997. 14-47.

(12) Linneberg A, Nielsen NH, Madsen F, Frolund L, Dirksen A, Jorgensen T. Is the increase in allergic respiratory disease caused by a cohort effect? Clin Exp Allergy 2002; 32(12):1702-1705.

(13) Ulrik CS, Backer V. Atopy in Danish children and adolescents: results from a longitudinal population study. Ann Allergy Asthma Immunol 2000; 85(4):293-297.

(14) Matricardi PM, Bjorksten B, Bonini S, Bousquet J, Djukanovic R, Dreborg S et al. Microbial products in allergy prevention and therapy. Allergy 2003; 58(6):461-471.

(15) Maizels RM, Yazdanbakhsh M. Immune regulation by helminth parasites: cellular and molecular mechanisms. Nat Rev Immunol 2003; 3(9):733-744.

(16) Ciprandi G, Cirillo I, Fenoglio D, Marseglia G, Tosca MA. Sublingual immunotherapy induces spirometric improvement associated with IL-10 production: Preliminary reports. Int Immunopharmacol 2006; 6(8):1370-1373.

(17) Lima MT, Wilson D, Pitkin L, Roberts A, Nouri-Aria K, Jacobson M et al. Grass pollen sublingual immunotherapy for seasonal rhinoconjunctivitis: a randomized controlled trial. Clin Exp Allergy 2002; 32(4):507-514.

(18) La RM, Ranno C, Andre C, Carat F, Tosca MA, Canonica GW. Double-blind placebo-controlled evaluation of sublingual-swallow immunotherapy with standardized Parietaria judaica extract in children with allergic rhinoconjunctivitis. J Allergy Clin Immunol 1999; 104(2 Pt 1):425-432.

(19) Gafvelin G, Thunberg S, Kronqvist M, Gronlund H, Gronneberg R, Troye-Blomberg M et al. Cytokine and antibody responses in birch-pollen-allergic patients treated with genetically modified derivatives of the major birch pollen allergen Bet v 1. Int Arch Allergy Immunol 2005; 138(1):59-66.

(20) Kitagaki K, Businga TR, Racila D, Elliott DE, Weinstock JV, Kline JN. Intestinal helminths protect in a murine model of asthma. J Immunol 2006; 177(3):1628-1635.

(21) Nagler-Anderson C. Helminth-induced immunoregulation of an allergic response to food. Chem Immunol Allergy 2006; 90:1-13.

(22) Wilson MS, Taylor MD, Balic A, Finney CA, Lamb JR, Maizels RM. Suppression of allergic airway inflammation by helminth-induced regulatory T cells. J Exp Med 2005; 202(9):1199-1212.

(23) Lynch NR, Hagel I, Perez M, Di Prisco MC, Lopez R, Alvarez N. Effect of anthelmintic treatment on the allergic reactivity of children in a tropical slum. J Allergy Clin Immunol 1993; 92(3):404-411.

(24) van den Biggelaar AH, Rodrigues LC, van RR, van der Zee JS, Hoeksma-Kruize YC, Souverijn JH et al. Long-term treatment of intestinal helminths increases mite skin-test reactivity in Gabonese schoolchildren. J Infect Dis 2004; 189(5):892-900.

(25) Lau S, Matricardi PM. Worms, asthma, and the hygiene hypothesis. Lancet 2006; 367(9522):1556-1558.

(26) Falcone FH, Pritchard DI. Parasite role reversal: worms on trial [Phase I results from hookworm trial was not published]. Trends Parasitol 2005; 21(4):157-160.

(27) Falcone FH, Pritchard DI. Parasite role reversal: worms on trial. Trends Parasitol 2005; 21(4):157-160.

(28) Feary J, Falcone FH, Pritchard D. "Hayfever sufferers: could you host a hookworm?" Title of advertisement for trial participants, posted on website for Nottingham University. Phase I of hookworm trial described: "A group of 10 (including the researchers) received 10, 25, 50 or 100 hookworm larvae and 10 larvae was found to produce a good response while also producing the fewest

side effects". Dep Epidemiology, Nottingham University, England [2006 [cited 2006 Aug. 14]; Available from: URL:www.alumni.nottingham.ac.uk/News/news.aspx?newsId=368

(29) Anonymous. "How to cure your asthma or hayfever using hookworm - a practical guide", unpublished case-story self-reported on the internet. www kuro5hin org/story/2006/4/30/91945/8971 [2006 [cited 2006 Aug. 15];

(30) Elliott DE, Summers RW, Weinstock JV. Helminths and the modulation of mucosal inflammation. Curr Opin Gastroenterol 2005; 21(1):51-58.

(31) Summers RW, Elliott DE, Weinstock JV. Why Trichuris suis should prove safe for use in inflammatory bowel diseases. Inflamm Bowel Dis 2005; 11(8):783-784.

(32) Beer RJ. Experimental infection of man with pig whipworm. Br Med J 1971; 2(752):44.

(33) Beer RJ. The relationship between Trichuris trichiura (Linnaeus 1758) of man and Trichuris suis (Schrank 1788) of the pig. Res Vet Sci 1976; 20(1):47-54.

(34) Summers RW, Elliott DE, Qadir K, Urban JF, Jr., Thompson R, Weinstock JV. Trichuris suis seems to be safe and possibly effective in the treatment of inflammatory bowel disease. Am J Gastroenterol 2003; 98(9):2034-2041.

(35) Summers RW, Elliott DE, Urban JF, Jr., Thompson RA, Weinstock JV. Trichuris suis therapy for active ulcerative colitis: a randomized controlled trial. Gastroenterology 2005; 128(4):825-832.

(36) Summers RW, Elliott DE, Urban JF, Jr., Thompson R, Weinstock JV. Trichuris suis therapy in Crohn's disease. Gut 2005; 54(1):87-90.

(37) Multi authorship. CAUSALL (acronym - The cause of allergy). The role of environment, infections, food and lifestyle in promoting or preventing allergic sensitisation and disease. 60 months EU integrated project with priority 5 - food quality and safety. 4th call topic T5.4.8.1. Available on request or contact coordinator Dr. Ronald van Ree (Amsterdam Medical Centre,Tel.+31-20-5669111). 2006.
Ref Type: Report

(38) Dahl R, Kapp A, Colombo G, de Monchy JG, Rak S, Emminger W et al. Efficacy and safety of sublingual immunotherapy with grass allergen tablets for seasonal allergic rhinoconjunctivitis. J Allergy Clin Immunol 2006; 118(2):434-440.

(39) Juniper EF, Thompson AK, Ferrie PJ, Roberts JN. Development and validation of the mini Rhinoconjunctivitis Quality of Life Questionnaire. Clin Exp Allergy 2000; 30(1):132-140.

(40) Frølund L. Rationel farmakoterapi: Farmakologisk behandling af allergiske lidelser i øjne og næse. 2006. Institut for Rationel Farmakoterapi, København, Lægeforeningens forlag.
Ref Type: Pamphlet

(41) Pellegrino R, Viegi G, Brusasco V, Crapo RO, Burgos F, Casaburi R et al. Interpretative strategies for lung function tests. Eur Respir J 2005; 26(5):948-968.

Appendix 2: Declaration of Helsinki

WORLD MEDICAL ASSOCIATION DECLARATION OF HELSINKI

Ethical Principles for Medical Research Involving Human Subjects

Adopted by the 18th WMA General Assembly Helsinki, Finland, June 1964 and amended by the
29th WMA General Assembly, Tokyo, Japan, October 1975
35th WMA General Assembly, Venice, Italy, October 1983
41st WMA General Assembly, Hong Kong, September 1989
48th WMA General Assembly, Somerset West, Republic of South Africa, October 1996
52nd WMA General Assembly, Edinburgh, Scotland, October 2000

A. INTRODUCTION

The World Medical Association has developed the Declaration of Helsinki as a statement of ethical principles to provide guidance to physicians and other participants in medical research involving human subjects. Medical research involving human subjects includes research on identifiable human material or identifiable data.

It is the duty of the physician to promote and safeguard the health of the people. The physician's knowledge and conscience are dedicated to the fulfilment of this duty.

The Declaration of Geneva of the World Medical Association binds the physician with the words, "The health of my patient will be my first consideration," and the International Code of Medical Ethics declares that, "A physician shall act only in the patient's interest when providing medical care which might have the effect of weakening the physical and mental condition of the patient."

Medical progress is based on research which ultimately must rest in part on experimentation involving human subjects.

In medical research on human subjects, considerations related to the well-being of the human subject should take precedence over the interests of science and society.

The primary purpose of medical research involving human subjects is to improve prophylactic, diagnostic and therapeutic procedures and the understanding of the aetiology and pathogenesis of disease. Even the best proven prophylactic, diagnostic, and therapeutic methods must continuously be challenged through research for their effectiveness, efficiency, accessibility and quality.

In current medical practice and in medical research, most prophylactic, diagnostic and therapeutic procedures involve risks and burdens.

Medical research is subject to ethical standards that promote respect for all human beings and protect their health and rights. Some research populations are vulnerable and need special protection. The particular needs of the economically and medically disadvantaged must be recognized. Special attention is also required for those who cannot give or refuse consent for themselves, for those who may be subject to giving consent under duress, for those who will not benefit personally from the research and for those for whom the research is combined with care.

Research Investigators should be aware of the ethical, legal and regulatory requirements for research on human subjects in their own countries as well as applicable international requirements. No national ethical, legal or regulatory requirement should be allowed to reduce or eliminate any of the protections for human subjects set forth in this Declaration.

B. BASIC PRINCIPLES FOR ALL MEDICAL RESEARCH

It is the duty of the physician in medical research to protect the life, health, privacy, and dignity of the human subject.

Medical research involving human subjects must conform to generally accepted scientific principles, be based on a thorough knowledge of the scientific literature, other relevant sources of information, and on adequate laboratory and, where appropriate, animal experimentation.

Appropriate caution must be exercised in the conduct of research which may affect the environment, and the welfare of animals used for research must be respected.

The design and performance of each experimental procedure involving human subjects should be clearly formulated in an experimental protocol. This protocol should be submitted for consideration, comment, guidance and, where appropriate, approval to a specially appointed ethical review committee, which must be independent of the Investigator, the Sponsor or any other kind of undue influence. This independent committee should

be in conformity with the laws and regulations of the country in which the research experiment is performed. The committee has the right to monitor ongoing trials. The researcher has the obligation to provide monitoring information to the committee, especially any serious adverse events. The researcher should also submit to the committee, for review, information regarding funding, sponsors, institutional affiliations, other potential conflicts of interest and incentives for subjects.

The research protocol should always contain a statement of the ethical considerations involved and should indicate that there is compliance with the principles enunciated in this Declaration.

Medical research involving human subjects should be conducted only by scientifically qualified persons and under the supervision of a clinically competent medical person. The responsibility for the human subject must always rest with a medically qualified person and never rest on the subject of the research, even though the subject has given consent.

Every medical research project involving human subjects should be preceded by careful assessment of predictable risks and burdens in comparison with foreseeable benefits to the subject or to others. This does not preclude the participation of healthy volunteers in medical research. The design of all studies should be publicly available.

Physicians should abstain from engaging in research projects involving human subjects unless they are confident that the risks involved have been adequately assessed and can be satisfactorily managed. Physicians should cease any investigation if the risks are found to outweigh the potential benefits or if there is conclusive proof of positive and beneficial results.

Medical research involving human subjects should only be conducted if the importance of the objective outweighs the inherent risks and burdens to the subject. This is especially important when the human subjects are healthy volunteers.

Medical research is only justified if there is a reasonable likelihood that the populations in which the research is carried out stand to benefit from the results of the research.

The subjects must be volunteers and informed participants in the research project.

The right of research subjects to safeguard their integrity must always be respected. Every precaution should be taken to respect the privacy of the subject, the confidentiality of the patient's information and to minimize the impact of the study on the subject's physical and mental integrity and on the personality of the subject.

In any research on human beings, each potential subject must be adequately informed of the aims, methods, sources of funding, any possible conflicts of interest, institutional affiliations of the researcher, the anticipated benefits and potential risks of the study and the discomfort it may entail. The subject should be informed of the right to abstain from participation in the study or to withdraw consent to participate at any time without reprisal. After ensuring that the subject has understood the information, the physician should then obtain the subject's freely-given informed consent, preferably in writing. If the consent cannot be obtained in writing, the non-written consent must be formally documented and witnessed.

When obtaining informed consent for the research project the physician should be particularly cautious if the subject is in a dependent relationship with the physician or may consent under duress. In that case the informed consent should be obtained by a well-informed physician who is not engaged in the investigation and who is completely independent of this relationship.

For a research subject who is legally incompetent, physically or mentally incapable of giving consent or is a legally incompetent minor, the Investigator must obtain informed consent from the legally authorized representative in accordance with applicable law. These groups should not be included in research unless the research is necessary to promote the health of the population represented and this research cannot instead be performed on legally competent persons.

When a subject deemed legally incompetent, such as a minor subject, is able to give assent to decisions about participation in research, the Investigator must obtain that assent in addition to the consent of the legally authorized representative.

Research on individuals from whom it is not possible to obtain consent, including proxy or advance consent, should be done only if the physical/mental condition that prevents obtaining informed consent is a necessary characteristic of the research population. The specific reasons for involving research subjects with a condition that renders them unable to give informed consent should be stated in the experimental protocol for consideration and approval of the review committee. The protocol should state that consent to remain in the research should be obtained as soon as possible from the individual or a legally authorized surrogate.

Both authors and publishers have ethical obligations. In publication of the results of research, the Investigators are

obliged to preserve the accuracy of the results. Negative as well as positive results should be published or otherwise publicly available. Sources of funding, institutional affiliations and any possible conflicts of interest should be declared in the publication. Reports of experimentation not in accordance with the principles laid down in this Declaration should not be accepted for publication.

C. ADDITIONAL PRINCIPLES FOR MEDICAL RESEARCH COMBINED WITH MEDICAL CARE

The physician may combine medical research with medical care, only to the extent that the research is justified by its potential prophylactic, diagnostic or therapeutic value. When medical research is combined with medical care, additional standards apply to protect the patients who are research subjects.

The benefits, risks, burdens and effectiveness of a new method should be tested against those of the best current prophylactic, diagnostic, and therapeutic methods. This does not exclude the use of placebo, or no treatment, in studies where no proven prophylactic, diagnostic or therapeutic method exists.

At the conclusion of the study, every patient entered into the study should be assured of access to the best proven prophylactic, diagnostic and therapeutic methods identified by the study.

The physician should fully inform the patient which aspects of the care are related to the research. The refusal of a patient to participate in a study must never interfere with the patient-physician relationship.

In the treatment of a patient, where proven prophylactic, diagnostic and therapeutic methods do not exist or have been ineffective, the physician, with informed consent from the patient, must be free to use unproven or new prophylactic, diagnostic and therapeutic measures, if in the physician's judgement it offers hope of saving life, re-establishing health or alleviating suffering. Where possible, these measures should be made the object of research, designed to evaluate their safety and efficacy. In all cases, new information should be recorded and, where appropriate, published. The other relevant guidelines of this Declaration should be followed

Appendix 3: Consent form

Informeret samtykke til deltagelse i et biomedicinsk forskningsprojekt

Forskningsprojektets titel:
Oral behandling af allergi med en ikke-patogen mikroorganisme:
Et randomiseret klinisk forsøg.

Erklæring fra den forsøgsansvarlige:
Jeg erklærer, at nedenstående forsøgsperson har modtaget mundtlig og skriftlig information om forskningsprojektet. Efter min overbevisning er der givet tilstrækkelig information, herunder om fordele og ulemper, til at træffe et informeret valg.
De forsøgsansvarliges navne:

____________________________ ___________ _____________________________

Klinikchef og speciallæge	Dato	Speciallæge, Ph.D.
John Arnved		Steen Rønborg

Samtykkeerklæring fra forsøgspersonen:
Jeg har læst den skriftlige information om forskningsprojektet og fået mundtlig information i et sprog, som jeg forstår. Jeg ved nok om formålet, metoderne, fordele og ulemper til at sige ja til at deltage. Jeg er informeret om, at det er frivilligt at deltage, og at jeg når som helst og uden begrundelse kan trække mit samtykke tilbage og udtræde af forsøget, uden at dette påvirker min ret til behandling eller andre rettigheder.

Jeg indvilger i at deltage i forskningsprojektet og har modtaget en kopi af dette samtykkeark samt en kopi af den skriftlige information til eget brug.

Jeg indvilger i at blive kontaktet efter hele forsøget er slut, fordi forskerne planlægger eventuelle yderligere undersøgelser, hvor der er brug for min hjælp (afkryds):

Ja ☐ **Nej** ☐

Fuldmagtserklæring fra forsøgspersonen
Jeg er informeret om og giver min fuldmagt til (jf. Sundhedsloven, nr. 546 af 24. juni 2005), at udvalgte medarbejdere fra GCP-enheden ved Københavns Universitetshospital, GCP-enhedens samarbejdspartnere, SSI's kvalitetsafdeling, Lægemiddelstyrelsen, eller tilsvarende myndighed fra udlandet kan få adgang til oplysninger i min patientjournal med det formål at sikre kvaliteten af forsøget.

Fuldmagten gælder i indtil 5 år efter forsøgets afslutning. Alle oplysninger vil blive behandlet strengt fortroligt.

Forsøgspersonens navn:____

________________Dato ____________________________Underskrift

Appendix 4: Participant information

DELTAGERINFORMATION

til klinisk forsøg med titlen

Oral behandling af allergi med en ikke-patogen mikroorganisme

Lunge- og Allergiklinikken København
Statens Serum Institut

Vi vil hermed spørge, om du i år 2008 vil deltage i et biomedicinsk forskningsforsøg på Lunge- og Allergiklinikken København. Forsøget er beskrevet nedenfor. Først når du har læst denne information grundigt igennem og modtaget mundtlig information fra forsøgets læge, kan du afgøre, om du vil give samtykke til at deltage i forsøget. Det er vigtigt at du stiller spørgsmål, hvis der er noget du ikke forstår. Denne information er til dit eget brug.

Introduktion

Du er behandlet her på Lunge- og Allergiklinikken København, fordi du har høfeber. Vi er i samarbejde med Statens Serum Institut i gang med et forsøg for at finde ud af, om indtagelse af mikroskopiske æg, der indeholder en harmløs mikroorganisme kan helbrede eller mindske dine symptomer på pollen-allergi.

Mange mikroorganismer findes naturligt i din krop. Den organisme, som vi vil anvende til behandling hedder *Trichuris suis* og er én der måske kan hjælpe dig og som er harmløs for kroppen. Vor teori er, at mikroorganismen naturligt vil stimulere dit immunsystem, så du får mindre pollenallergi og dermed flere dage i pollensæsonen, hvor du er rask. Du vil hverken kunne se æg eller mikroorganisme på noget tidspunkt.

Både allergi og andre kroniske inflammatoriske sygdomme er steget i forekomst i mange velfærdslande og således også i Danmark. Man har haft mistanke, om at årsagen til stigningen er, at mikroorganismer, der naturligt kan stimulere vores immunsystem, er forsvundet på grund af vores mere hygiejniske levestil. Den nyeste forskning peger stærkt

på, at netop den type mikroorganisme, som foreslås til behandling her, har haft stor betydning.

Behandlingen med mikroorganismen har vist sig at være effektiv mod kroniske inflammatoriske mavetarmsygdomme (Morbus Crohn og Ulcerøs colitis). Her viste behandling af 90 patienter, at regelmæssig behandling havde den bedste effekt og først efter 4-6 uger. Den samme regelmæssige behandling anvendes derfor også i dette forsøg.

Selve forsøget

Forsøget er tilrettelagt sådan, at halvdelen af de 100 deltagere hver 3. uge vil skulle drikke af en lille flaske, der indeholder en vandig opløsning med 2500 mikroskopiske æg med *Trichuris suis*. Vandet i flasken vil se ud som almindeligt vand, og vil også dufte og smage af almindeligt vand. Den anden halvdel af deltagerne vil på samme vis skulle drikke af en flaske men uden æg (placebo gruppe). Du vil skulle komme på klinikken for at modtage behandlingen.

Allergiklinikken vil forsyne dig med gratis allergimedicin for det tilfælde, at du får symptomer på høfeber eller eventuelt astma, og samtidig hjælpe dig med at lægge en plan, hvis du får brug for medicinen.

Hele forsøget vil starte i første kvartal af 2008 og vare i 5-6 måneder.
I den periode vil du skulle

- Besøge Allergiklinikken 6-9 gange. Modtage forsøgsbehandlingen i alt 5-8 gange.
- Udfylde en skematisk dagbog, hvor du bl.a. daglig på en skala vurderer dine eventuelle symptomer på pollenallergi og astma, og hver uge vurderer din livskvalitet.
- Dagligt måle din lungefunktion med et lille apparat (Piko-1), som du får udleveret.
- 3 gange få taget en blodprøve (i starten, midten, og slutningen af forsøget)
- 3-4 gange få målt graden af en eventuel allergisk betændelse i dine luftveje ved at puste i et apparat på klinikken (udåndet nitrogen-oxid).
- 2 gange blive hudpriktestet overfor allergener

Cirka 1½ måned efter du er færdig med forsøget vil du skulle udfylde et spørgeskema og have et møde med lægen, alt sammen for at undersøge om du er tilfreds med forsøgets forløb.

For at hverken du selv eller allergiklinikkens læger skal vide, om du får æg eller ej, vil vi trække lod om, hvem der skal have æg, og hvem der ikke skal have æg. Det skal sikre, at du (eller lægen) ikke blot tror, at du har det bedre, hvis du ved, at du får æg.

Når forsøget er slut, kan vi finde ud af, om du har fået æg eller ej ved at bryde en kode. Hvis du er interesseret i at få at vide, om du har fået æg, kan du sige det til os med det samme, eller ringe til os senere. Så sender vi svaret til dig, når forsøget er slut, og vil samtidig eller senere informere dig om resultatet af forsøget. Hele forsøget er bedømt og godkendt af de Videnskabsetiske komitéer for Københavns og Frederiksberg kommuner (sag nr. 01 2006-4100), og Lægemiddelstyrelsen (sag nr. 2612-3616).

Hvem kan deltage i forsøget?

Du kan være med i forsøget hvis du er mellem 18 og 65 år, og har græs- og/eller birke-pollenallergi. Din lungefunktion skal være over 70% af forventet, og du må ikke have alvorlig astma.

Hvis du er kvinde må du ikke være fødedygtig (hvis du har overstået overgangsalderen eller af anden årsag ikke er fødedygtig, f.eks. steriliseret, må du gerne deltage).

Er der nogen risiko eller ubehag forbundet med forsøget?

- Forsøget indebærer ingen kendte risici, bivirkninger, eller ubehag.
- Du vil ikke på noget tidspunkt kunne se æg eller mikroorganisme.
- Mikroorganismen kan fjernes med uskadelig tabletmedicin.

Produktet med æg er kvalitetssikret af fremstiller, sygehusapotek, og Statens Serum Institut. Såfremt der optræder uforudsigelige komplikationer vil du straks kunne få en kort behandling med en uskadelig tablet medicin, der dræber mikroorganismen. Du vil blive fuldt orienteret, såfremt der blandt andre deltagere optræder uforudsigelige komplikationer, der medfører at forsøget som helhed må afbrydes. Du vil også blive orienteret, hvis der fremkommer nye oplysninger om effekt, risici, bivirkninger, komplikationer, eller ulemper ved behandlingen. Ved kliniske forsøg er du omfattet af gældende erstatningsbestemmelser, jævnfør lov om patientforsikring eller spørg en af forsøgets læger. Det er naturligvis vigtigt, at du er bevidst om, at der ved deltagelse i kliniske forsøg vil kunne forekomme uforudsigelige risici.

Er der nogen fordele ved at deltage i forsøget?

Hvis du bliver en af de 50 der modtager behandling med æg, mener vi det er en fordel for dig, fordi behandlingen muligvis kan mindske dine symptomer på pollenallergi. Behandlingen har ingen kendte bivirkninger - i modsætning til en del almindelig

allergimedicin. En fordel for alle er mulighed for bedre viden og kontrol af deres allergisymptomer, fordi du gennem dagbøgerne, de daglige lungefunktionsmålinger, og konsultationerne med lægen, vil få mere at vide om din allergi end ellers. Desuden vil allergiklinikken udlevere gratis allergimedicin til behandling af eventuelle symptomer.

Hvis behandlingen med æg er effektiv vil andre allergikere også kunne drage nytte af denne behandling.

Du vil blive godtgjort for 2-3 minutter af den tid du har brugt hver dag på at udfylde dagbøger og måle din lungefunktion med 150kr/time (ca. 1.000 kr i alt). Du vil derudover kunne få godtgjort dokumenteret tabt arbejdsfortjeneste på op til 15 minutter per konsultation (150 kr/time), samt dokumenterede transportudgifter til og fra besøgene på klinikken på op til 100 kr per besøg. Godtgørelsen er skattepligtig, undtagen hvad angår transportgodtgørelse.

Det skal derudover oplyses, at klinikkens økonomiske fordele ved at deltage i forsøget er, at Statens Serum Institut betaler klinikken ialt 1.500 kr for samtlige konsultationer med dig, 45.000 kr for 3 måneders leje af apparatet, der måler din udåndingsluft for nitrogen-oxid, og fuld løn til 2 sygeplejersker, der skal hjælpe klinikkens læger under forsøget. Forsøget er iværksat af forskere fra Statens Serum Institut og det Biomedicinske Fakultet, sammen med Lunge- og Allergiklinikken København. Æggene leveres gratis af fremstiller, Ovamed GmbH i Tyskland (www.ovamed.de). Hele forsøget er finansieret af Forskningsrådet for Sundhed og Sygdom (1,5 millioner kr), samt flere private fonde uden økonomiske interesser i æggene som produkt.

Fortrolighed

Alle oplysninger til forsøget vil blive indtastet, opbevaret og analyseret i computer. Alle oplysninger vedrørende dine helbredsforhold er omfattet af tavshedspligt i henhold til lov om patienters retstilling og straffeloven. Din anonymitet vil blive bevaret. Foruden omstående personer har Lægemiddelstyrelsen, de Videnskabsetiske komitéer, og de ansvarlige forskere fra Statens Serum Institut adgang til oplysningerne som du bidrager med under forsøget. For at sikre at forsøget foregår helt korrekt, vil dertil ansatte personer fra udenlandske myndigheder, Lægemiddelstyrelsen og Statens Serum Institut have adgang til din patientjournal på Lunge- og Allergiklinikken København. Forsøget er godkendt af Datatilsynet (J.nr. 2006-54-2215).

Det er frivilligt at deltage

Deltagelse i forsøget er fuldstændig frivilligt. Hvis du beslutter dig for at deltage og senere fortryder, kan du til enhver tid og uden begrundelse trække dig ud, også selvom du ”har skrevet under”. Hvis du vælger ikke at deltage i forsøget, vil det ikke påvirke din behandling eller dit forhold til Lunge- og Allergiklinikken København og dens personale. Du har ret til betænkningstid, før du giver samtykke, og du kan medbringe en bisidder ved modtagelse af den mundtlige information. Du har mulighed for at få aktindsigt i forsøgsprotokollen efter offentlighedslovens bestemmelser. Vedlagt er et brev til dig om *Dine rettigheder som forsøgsperson i et biomedicinsk forsøg*, som er skrevet af Den Centrale Videnskabsetiske Komité.

Kontaktperson

Har du spørgsmål til forsøget kan du altid kontakte en af forsøgets læger på Lunge- og Allergiklinikken København.

Speciallæge John Arnved og Steen Rønborg
Lunge- og Allergiklinikken København,
Frederiksborggade 15, 7. sal
DK- 1360 København
Telefon: 3313 6513 (klinikken), 3313 6502 (direkte)

Hvis du indvilliger i at deltage, bedes du læse og underskrive den informerede samtykkeerklæring. Du vil få udleveret en kopi af den underskrevne samtykkeerklæring.

Med venlig hilsen

____

John Arnved/Steen Rønborg

DEN CENTRALE
VIDENSKABSETISKE
KOMITÉ

The Danish National Committee
for Biomedical Research Ethics

Slotsholmsgade 12
DK-1216 København K
Tel +45 3395 5626
Fax +45 3395 5635
cvk@im.dk
www.cvk.im.dk

Til deltagere i biomedicinske forskningsprojekter

Dine rettigheder som forsøgsperson i et biomedicinsk forskningsprojekt.

Som deltager i et biomedicinsk forskningsprojekt skal du vide at :

-din deltagelse i forskningsprojektet er helt frivillig og kun kan ske efter, at du har fået både skriftlig og mundtlig information om forskningsprojektet og underskrevet samtykkeerklæringen,

- du til enhver tid mundtligt, skriftligt eller ved anden klar tilkendegivelse kan trække dit samtykke til deltagelse tilbage og udtræde af forskningsprojektet. Såfremt du trækker dit samtykke tilbage påvirker dette ikke din ret til nuværende eller fremtidig behandling eller andre rettigheder, som du måtte have,

- du har ret til at tage et familiemedlem, en ven eller en bekendt med til informationssamtalen,

- du har ret til betænkningstid, før du underskriver samtykkeerklæringen

- oplysninger om dine helbredsforhold, øvrige rent private forhold og andre fortrolige oplysninger om dig, som fremkommer i forbindelse med forskningsprojektet, er omfattet af tavshedspligt,

- opbevaringen af oplysninger om dig, herunder oplysninger i væv, blodprøver, der hidrører fra dig, sker efter reglerne i lov om behandling af personoplysninger og lov om patienters retsstilling

- der er mulighed for at få aktindsigt i forsøgsprotokoller efter offentlighedslovens bestemmelser herom. Det vil sige, at du kan få adgang til at se alle papirer vedrørende din deltagelse i forsøget, bortset fra de dele, som indeholder forretningshemmeligheder eller fortrolige oplysninger om andre.

- der er klageadgang, muligheder for erstatning efter patientforsikringsloven eller lov om erstatning for lægemiddelskader samt anden kompensation ved skader som følge af forskningsprojektet.

(Ovenstående tillæg er udgivet af Den Centrale Videnskabsetiske komité og skal vedhæftes den skriftlige information om det biomedicinske forskningsprojekt)

Appendix 5: Investigator's statement

Investigators erklæring

Klinisk forsøg: Oral behandling af allergi med en ikke-patogen mikroorganisme: Et randomiseret klinisk forsøg TSO-01.

Dette kliniske forsøg vil blive udført i overenstemmelse med God Klinisk Praksis (GCP) defineret af den Europæiske Union og principperne i Helsinki deklarationen. Specielt vil de følgende punkter blive fulgt:

- Den endelige forsøgsprotokol og efterfølgende ændringer vil blive fulgt.
- Alle deltager oplysninger, inklusiv uønskede hændelser, vil blive komplet og nøjagtigt indskrevet i Case Report Forms (CRFs).
- Alvorlige uønskede hændelser og bivirkninger, som defineret i protokollen, vil blive rapporteret til de passende regulatoriske myndigheder, etiske komitteer, og til Statens Serum Institut, som foreskrevet i instruktionerne i protokollen.
- Monitor vil blive tilladt adgang til deltagernes patient journaler.
- Før indgang i forsøget skal alle deltagere give skriftligt samtykke.
- De orale behandlingsdoser, korrekt opbevaret, vil kun blive givet til deltagerne i forsøget under de forsøgsansvarlige lægers ansvar. Der kan gøres regnskab for alle brugte æsker i loggen for behandlingsdoserne. Ubrugte æsker med behandlingsdoser vil blive returneret til apoteket.
- "Investigators File", indeholdende underskrevede samtykkeerklæringer, screening logs, korrespondance og andre oplysninger tilhørende forsøget, vil blive opbevaret på Lunge- og Allergiklinikken København under rekrutterings/behandlingsfasen af forsøget. "Investigators File" vil blive arkiveret af de forsøgsansvarlige læger i 15 år efter forsøget er slut.
- Den primære forsøgsansvarlige læge vil tillade bekræftelse af kildedata og auditering udført af Statens Serum Institut og inspektion af sundhedsmyndigheder.

Jeg indvilger i denne udtalelse's betingelser og i Statens Serum Institut's generelle retningslinjer for udførelse af kliniske forsøg.

Date: ___________________________ Signature: ____

John Arnved,
Primær forsøgsansvarlig læge

Appendix 6: Indemnity Statement

Trial title: Oral treatment of allergy with a non-pathogenic micro-organism: A randomised clinical trial.

Dear John Arnved,

You have kindly agreed to consider undertaking the above-mentioned clinical trial of ParaTech's and Ovamed's product with *Trichuris suis* eggs and placebo as investigator, in accordance with the protocol for the trial TSO-01 initiated and coordinated by Statens Serum Institut.

In the event that any recruited subject in the trial should suffer any personal injury resulting from the clinical trial, Statens Serum Institut agrees to indemnify the institution where the clinical trial is being undertaken (Pulmonology and Allergy Clinic Copenhagen) any of its employees or agents participating in the trial, against liability imposed by law, but not assumed voluntarily, and arising from the use of the *Trichuris suis* eggs and the placebo in the trial, PROVIDED THAT:

Statens Serum Institut shall not indemnify against, nor have any obligation whatsoever as regards liability arising from or related to any error, omission, intentional wrongful act, or other negligence on the part of said institutions or persons, such as medical malpractice; and

Any such institution or person seeking indemnity

has fully complied with the trial protocol, and

has promptly notified Statens Serum Institut of any notice of any type of claim, or the likelihood of a claim, relating to the trial,

as regards any claim, makes no statement, takes no action, nor makes any commitment affecting Statens Serum Institut's interests, without Statens Serum Institut's prior written consent, and further, provides all reasonable and necessary assistance to Statens Serum Institut in the defence of any claim, allowing Statens Serum Institut, at its cost and in its discretion, to take over the defence of any action and to have full control in handling the claim.

Please note that this letter is not a legal contract itself, but rather summarizes the main points of Statens Serum Institut's liability under its legal and economical agreement with ParaTech, Ovamed, and the Pharmacy that labels and packs the final product.

Date: ____________________ Signature: ______________________________

Mads Melbye
Executive Vicepresident
Statens Serum Institut

Date: ____________________ Signature: ______________________________

Peter Bager
Study Director
Statens Serum Institut

Appendix 7: Scores for relief medication

Table. Scoring system used to calculate the daily score for use of relief medication used in a stepwise manner depending on severity of symptoms (step 1-3 for allergic rhinitis, and step A, B, and C for asthma). Participants are unaware of the scoring system, record daily in their diaries any use of medication (type, dose, and frequency of dosages), and must return all unused relief medication to the PACC, thus making it possible to check the exact amount of medication used.

Step	Medicine	Score/dosis	Max./day (guidance)
Allergic rhinitis			
1	Desloratidine 5 mg once daily	6	6
1	Levocabastine eye drops (0,5 mg/ml; 1 drop in each eye 2x daily)	2	8
2	Budesonide nasal spray (up to 32 µg; 2 puffs per nostril 2x daily)	1 per puff	8
3	Prednisone (up to 50 mg once daily)	1.6 per 5 mg	16
	Maximum daily score		38
Asthma			
A	Airomir (100 µg per inhalation; 1–2 inhalations twice daily)	2	8
B	Budesonide (200 µg per inhalation; 1–2 inhalations twice daily)	2	8
C	Prednisone	1.6 per 5 mg	16
	Maximum daily score		32

Appendix 8: Guidelines for verbal information of participants

Volunteers have a right to receive information about the clinical trial, which the volunteers consider to participate in, according to the order of the Ethical Committee § 16 and §7 about information and consent for involvement of subjects in biomedical research projects. The follow guidelines apply (In Danish)

Forsøgspersonen har krav på at få information om det biomedicinske forskningsprojekt, som denne påtænker at deltage i, jf. komitélovens § 16 og § 7 i bekendtgørelse om information og samtykke ved inddragelse af forsøgspersoner i biomedicinske forskningsprojekter.

Forsøgsprotokollen skal være vedlagt retningslinier for afgivelse af mundtlig deltagerinformation, jf. § 8 i bekendtgørelsen. Retningslinierne skal gælde for den person, som i praksis giver informationen. Ansvaret for at give den mundtlige information kan delegeres til en person, som har de faglige forudsætninger for at kunne formidle indholdet af forskningsprojektet, og som har direkte tilknytning til forskningsprojektet, jf. § 7, stk. 3 i bekendtgørelsen.

Retningslinierne for afgivelse af mundtlig information skal tage udgangspunkt i den skriftlige information. Retningslinierne for afgivelse af mundtlig information kan omhandle nedenstående forholdsregler fra bekendtgørelsens §§ 7 og 8.

Informationssamtalen

1. En informationssamtale kan kun finde sted efter forudgående aftale om tid og sted for informationssamtalen. Den mundtlige information vil finde sted før den skriftlige information.

2. Det skal angives, at der er tale om en forespørgsel om deltagelse i et biomedicinsk forskningsprojekt.

3. Samtalen skal indeholde en forståelig fremstilling af forskningsprojektet uden brug af tekniske eller værdiladede vendinger og gives på en hensynsfuld måde tilpasset modtagerens individuelle forudsætninger m.h.t. alder, modenhed, erfaring m.v. Samtalen skal foregå i uforstyrrede rammer, og der skal være tid til at stille spørgsmål.

4. Overvejelser omkring betænkningstid, dvs. at det skal angives hvor lang tid personen har til at overveje evt. deltagelse. Denne bør afhænge af forsøgets karakter.

5. Informationen skal indeholde oplysning om eventuelle forudsigelige risici, bivirkninger, komplikationer og ulemper, samt at der kan være uforudsigelige risici og belastninger knyttet til deltagelse i et biomedicinsk forskningsprojekt.

6. Informationen skal indeholde oplysninger om alternative behandlingsmetoder, jf. lov om patienters retsstilling.

7. Informationen skal indeholde oplysninger om forhold, som forsøgspersonen i øvrigt skønnes at være uvidende om, men som har betydning for forsøgspersonens stillingtagen, fx at vederlag til deltagerne er skattepligtige.

8. Den person som afgiver informationen skal sikre sig, at samtalen foregår uden afbrydelser og har ansvar for, at informationen er forstået.

Efterfølgende information.

9. Forsøgspersonen skal informeres, såfremt der under gennemførelsen af forsøget fremkommer *nye* oplysninger om effekt, risici, bivirkninger, komplikationer eller ulemper.

10. Den forsøgsperson, der fortsat er aktivt med i forsøget, skal informeres, såfremt forskningsprojektets forsøgsdesign ændres væsentligt i forhold til forsøgspersonens sikkerhed.

11. Forsøgspersonen skal informeres, såfremt der under gennemførelsen af forskningsprojektet fremkommer væsentlige oplysninger om forsøgspersonens helbredstilstand, medmindre forsøgspersonen utvetydigt har givet udtryk for, at den pågældende ikke ønsker dette, jf. bekendtgørelsens § 13.

12. Hvis det er praktisk muligt, og forsøgspersonen ønsker det, skal den forsøgsansvarlige eller en bemyndiget person ved forskningsprojektets afrapportering informere forsøgspersonen om de resultater, der er opnået, samt om eventuelle konsekvenser for den enkelte.

Appendix 9: Subject Screeening Log

Protokol: Oral behandling af allergi med en ikke-patogen mikroorganisme

Enkeltcenter: Lunge- og Allergiklinikken København

Investigator: John Arnved (ansvarlig) og Steen Rønborg

Screening-dato	Køn	Alder (år)	Evt.	Inkluderet ja/nej	Evt. årsag til ikke-inklusion

Til vitterlighed for dette dokument:
(udfyldes ved forsøgsafslutning)

Investigator:

John Arnved/
Steen Rønborg ________ ________________ ________________________________
Navn Dato Signatur

Appendix 10: Subject Identification Code List

Forsøgsperson Identifikationsliste

Protokol: Oral behandling af allergi med en ikke-patogen mikroorganisme

Enkeltcenter: Lunge- og Allergiklinikken København

Investigator: John Arnved (ansvarlig) og Steen Rønborg

**Nr. / id.**	**Navn**	**Initialer**	**CPR-nr.**	**Inklusions-*dato* og *læges initialer***	**Evt. ophørt i forsøg *dato***

Til vitterlighed for dette dokument:
(udfyldes ved forsøgsafslutning)

Investigator:

John Arnved/
Steen Rønborg ________ ________________ ________________________________
Navn Dato Signatur

Appendix 11: Subject Enrolment Log

Deltager indrulleringsliste

(to be generated as anonymous subject identification code list)

Appendix 12: Product accountability document

Id. nr.	Initialer	Dose nr.<br>Skriv dato for behandling.<br>Skriv ”R” bagefter udleveringsdato når den tomme flaske er returneret<br>Skriv ”S” foran dato ved udlevering til selvbehandling							
		1	2	3	4	5	6	7	8

Til vitterlighed for dette dokument: *(udfyldes ved forsøgsafslutning)*
Investigator: John Arnved/Steen Rønborg ________________ ____________________________________
Dato Signatur

Appendix 13: Check list for Investigator's and Sponsor's File

The list assists in succesfull management of the trial

The items are usually audited by inspectors

Before, during, or after trial the follow items are:	located in Sponsor's (S) and/or Investigator's (I) File		
(Marks of "*" are defined in each row)	before	during	after
Protocol (signed)	S+I	+update	S+I
- measuring ranges/normal ranges for tests	S+I	+update	S+I
- sample of labels for investigational products	S	S	S
- instructions for handling of the products	S+I	S+I	S+I
- decoding procedure	S+I	S+I	S+I
- participant information	S+I	S+I	S+I
- sample CRF	S+I	S+I	S+I
Investigator's brochure	S+I	+update	S+(I)
Consent forms (*signed)	S+I	I*	I
Financial aspects of the trial	S+I	S+I	S+I
Insurance statement	S+I	S+I	S+I
Signed agreements			
- Investigator (PACC)/sponsor (SSI)	S+I	S+I	S+I
- Sponsor (SSI)/Manufacturer (Ovamed GmbH)	S+I	S+I	S+I
- Sponsor (SSI)/Pharmacy Services at KVL	S+(I)	S+I	S+I
Approvals (dated and signed)			
- Ethical Comité of Copenhagen and	S+I	S+I	S+I
Frederiksberg Municipalities	S+I	+update	S+I
- The Danish Medicine Agency	S+I	+update	S+I
- The Data Protection Agency	S+I	S+I	S+I
Document of composition of the Ethical Comité	S+I	S+I	S+I
Curriculum vitae of Investigator and co-Investigator		+update	S
Documents of competence			
- Phadia ApS; accreditation or audit certificate	S	+update	S+I
- Ovamed GmbH, GMP approval	S	+update	S+I
- Pharmacy Services, GMP approval	S	+update	S+I
Transport documentation (method, accountability, temperature logging)			
- Products; Ovamed GmbH to Phamacy Services	S	S	S
- Products; Pharmacy Services to PACC	S	S+I	S+I
- Blood samples; PACC to Phadia ApS		S+I	S
Certificate of analysis of transported products			
- Ovamed GmbH (identity, purity, strength)	S	S	S
- Pharmacy Services (identity, certification)	S+I	S+I	S
Master randomisation list (*Pharmacy Services)	S*	S*+I	S
Allocation list for Statistician (*Pharmacy Services)	S*	S*	S+I
Blood randomisation list (*+Pharmacy Services)	S*	S*+I	S+I
Decoding documentation		I	S
Monitoring reports			
- Pre-trial and initiation report(s)	S+I	S+I	S+I
- Visit reports		S	S
- close-out report			S

(continued)

Before, during, or after trial the follow items are:	located in Sponsor's (S) and/or Investigator's (I) File		
(Marks of "*" are defined in each row)	before	during	after
Audit certificate (if any)			S
Communications (letters, meetings, telephone)		S+I	S
Souce documents (*transport to S, see 10.1 & 13.2)		S*+I	S+I
CRFs (signed, dated, and completed), *copy		S*+I	S*+I
CRF; document of corrections (*copy)		S*+I	S*+I
CRF; signatures, persons authorised to entry to CRF		S+I	S+I
Serious Adverse Event notification from I to S		S+I	S+I
Safety notification to EC and/or DMA		S+I	S+I
Safety notification from S to I		S+I	S+I
Subject screening log		I+(S)	I
Subject identification code list		I	I
Subject enrolment log		I	S+I
Product accountability document		S+I	S+I
Destruction documentation for product (if any)			S
Summary report by S (and/or I) to DMA			S or I
Clinical Study Report			S+(I)
