# Supplementary material for: Symptoms after Ingestion of Pig Whipworm Trichuris suis Eggs in a Randomized Placebo-Controlled Double-Blind Clinical Trial
Source: PLoS One. 2011 Aug 2;6(8):e22346. doi: 10.1371/journal.pone.0022346 (PMC3149054; doi:10.1371/journal.pone.0022346)
Supplement: Checklist S1 — CONSORT Checklist. (DOC) [file pone.0022346.s002.doc]

#
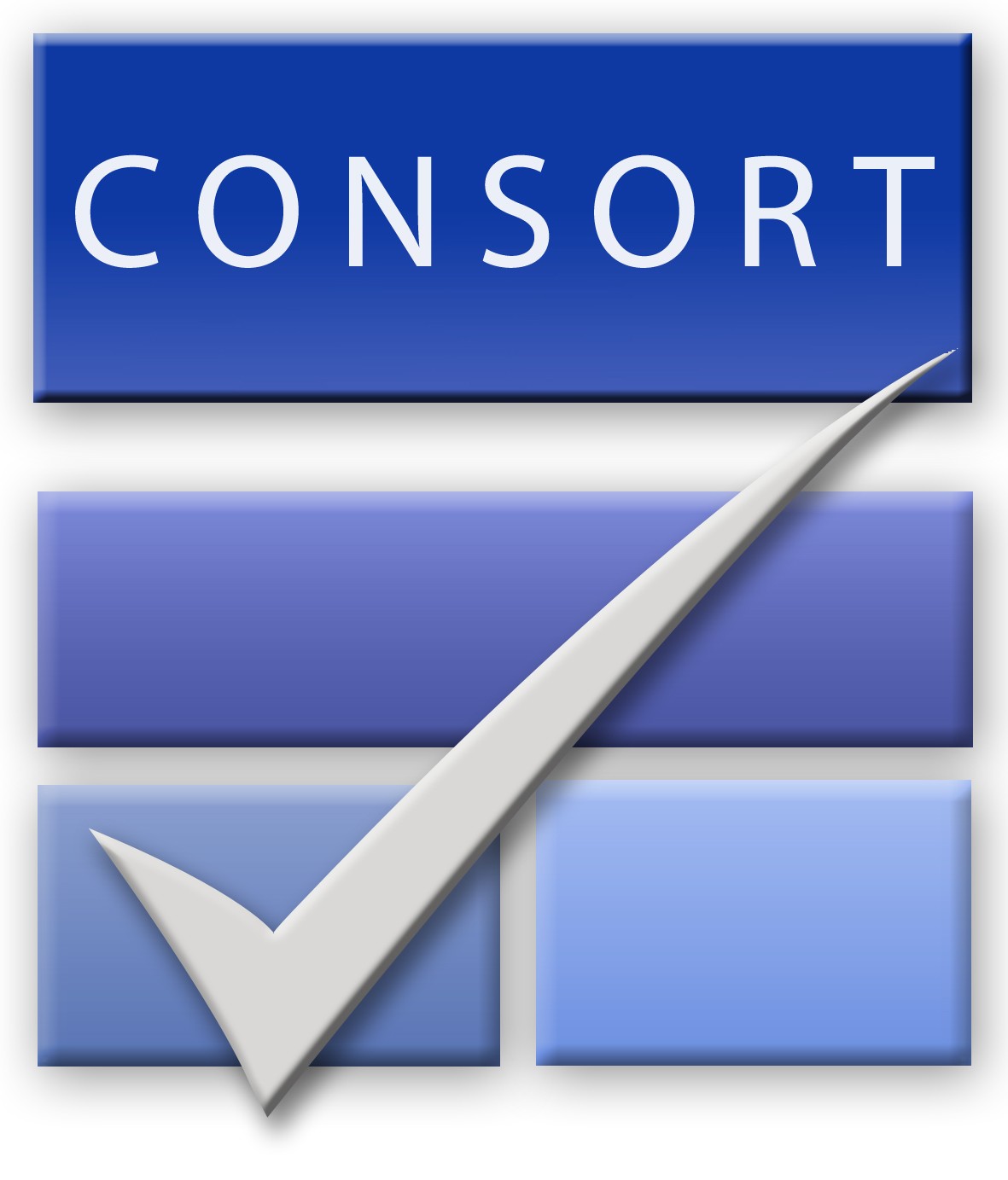
CONSORT Statement 2001 Checklist

**Items to include when reporting a randomized trial**

| ***PAPER SECTION* And topic** | Item | **Descriptor** | **Reported on**  **Page #** |
| --- | --- | --- | --- |
| TITLE & ABSTRACT | 1 | [How participants were allocated to interventions](http://www.consort-statement.org/index.aspx?o=1107) (*e.g*., "random allocation", "randomized", or "randomly assigned"). | Methods (Subjects and study design) |
| *INTRODUCTION* Background | 2 | [Scientific background and explanation of rationale](http://www.consort-statement.org/index.aspx?o=1016). | Introduction |
| *METHODS* Participants | 3 | [Eligibility criteria for participants](http://www.consort-statement.org/index.aspx?o=1017#3a) and the [settings and locations where the data were collected](http://www.consort-statement.org/index.aspx?o=1017#3b). | 2* |
| Interventions | 4 | [Precise details of the interventions intended for each group and how and when they were actually administered](http://www.consort-statement.org/index.aspx?o=1021). | 2* |
| Objectives | 5 | [Specific objectives and hypotheses](http://www.consort-statement.org/index.aspx?o=1022). | 2* |
| Outcomes | 6 | [Clearly defined primary and secondary outcome measures](http://www.consort-statement.org/index.aspx?o=1023#6a) and, when applicable, any [methods used to enhance the quality of measurements](http://www.consort-statement.org/index.aspx?o=1023#6b) (*e.g.*, multiple observations, training of assessors). | 2* |
| Sample size | 7 | [How sample size was determined](http://www.consort-statement.org/index.aspx?o=1024#7a) and, when applicable, [explanation of any interim analyses and stopping rules](http://www.consort-statement.org/index.aspx?o=1024#7b). |  |
| Sample size was not determined for the studied outcome of adverse events mainly because the observed side effects were unexpected and not the primary outcome. The primary outcome was reported in a previous study,* and here determination of sample size adhered to the CONSORT guideline (request details from the authors, if required) | | | |
| Randomization -- Sequence generation | 8 | [Method used to generate the random allocation sequence, including details of any restrictions](http://www.consort-statement.org/index.aspx?o=1025) (*e.g*., blocking, stratification) | 2* |
| Randomization -- Allocation concealment | 9 | [Method used to implement the random allocation sequence](http://www.consort-statement.org/index.aspx?o=1026) (*e.g*., numbered containers or central telephone), clarifying whether the sequence was concealed until interventions were assigned. | 2* |
| Randomization -- Implementation | 10 | [Who generated the allocation sequence, who enrolled participants, and who assigned participants to their groups](http://www.consort-statement.org/index.aspx?o=1027). | 2* |
| Blinding (masking) | 11 | [Whether or not participants, those administering the interventions, and those assessing the outcomes were blinded to group assignment](http://www.consort-statement.org/index.aspx?o=1028#11a). If done, [how the success of blinding was evaluated](http://www.consort-statement.org/index.aspx?o=1028#11b). | 3*, Methods (Adverse events) , Results (Study flow, subjects characteristics and blinding) |
| Statistical methods | 12 | [Statistical methods used to compare groups for primary outcome(s)](http://www.consort-statement.org/index.aspx?o=1029#12a); [Methods for additional analyses](http://www.consort-statement.org/index.aspx?o=1029#12b), such as subgroup analyses and adjusted analyses. | Methods (Statistical analyses), and 4* |
| RESULTS Participant flow | 13 | [Flow of participants through each stage](http://www.consort-statement.org/index.aspx?o=1018) (a diagram is strongly recommended). Specifically, for each group report the numbers of participants randomly assigned, receiving intended treatment, completing the study protocol, and analyzed for the primary outcome. [Describe protocol deviations from study as planned, together with reasons](http://www.consort-statement.org/index.aspx?o=1086). | 3 (fig. 1)*, Results (Study flow, …; Treatment compliance) |
| Recruitment | 14 | [Dates defining the periods of recruitment and follow-up](http://www.consort-statement.org/index.aspx?o=1087). | 2* |
| Baseline data | 15 | [Baseline demographic and clinical characteristics of each group](http://www.consort-statement.org/index.aspx?o=1088). | 4* |
| Numbers analyzed | 16 | [Number of participants (denominator) in each group included in each analysis and whether the analysis was by "intention-to-treat"](http://www.consort-statement.org/index.aspx?o=1089). State the results in absolute numbers when feasible (*e.g*., 10/20, not 50%). | 3 (fig. 1)*, Results (Study flow, …) |
| Outcomes and estimation | 17 | [For each primary and secondary outcome, a summary of results for each group, and the estimated effect size and its precision](http://www.consort-statement.org/index.aspx?o=1090) (*e.g.*, 95% confidence interval). | * and Results, and tables |
| Ancillary analyses | 18 | [Address multiplicity by reporting any other analyses performed](http://www.consort-statement.org/index.aspx?o=1091), including subgroup analyses and adjusted analyses, indicating those pre-specified and those exploratory. | Abstract, Introduction, Discuission |
| The study presents ancillary analyses only. Some of the analyses were pre-specified and some not. If the reviewers wish that the authors describe which analyses were pre-specified and which were not, the authors are willing to do so, however, this differentiation is very clear if the reader compares the present study with the previously published paper containing the main results of the study.* | | | |
| Adverse events | 19 | [All important adverse events or side effects in each intervention group](http://www.consort-statement.org/index.aspx?o=1092). | Results, tables, figures |
| *DISCUSSION* Interpretation | 20 | [Interpretation of the results](http://www.consort-statement.org/index.aspx?o=1019), taking into account study hypotheses, sources of potential bias or imprecision and the dangers associated with multiplicity of analyses and outcomes. | Discussion |
| Generalizability | 21 | [Generalizability (external validity) of the trial findings](http://www.consort-statement.org/index.aspx?o=1094). | Discussion |
| Overall evidence | 22 | [General interpretation of the results in the context of current evidence](http://www.consort-statement.org/index.aspx?o=1095). | Discussion |

*From* Moher D, Schulz KF, Altman DG. The CONSORT statement: revised recommendations for improving the quality of reports of parallel-group randomised trials. Lancet 2001; 357(9263):1191-1194.

**The CONSORT Statement 2001 checklist is intended to be accompanied with the explanatory document that facilitates its use. For more information, visit** [**www.consort-statement.org**](http://www.consort-statement.org/)**.**

***** Bager P, Arnved J, Ronborg S, Wohlfahrt J, Poulsen LK, Westergaard T et al. Trichuris suis ova therapy for allergic rhinitis: a randomized, double-blind, placebo-controlled clinical trial. J Allergy Clin Immunol 2010; 125(1):123-130

**The Consort E-Flowchart Aug. 2005**

**Trial:** *Trichuris suis* **ova therapy for allergic rhinitis.**

Assessed for eligibility (n= 162 )

Excluded (n= 62 )

Not meeting inclusion criteria

(n= 46 )

Refused to participate

(n= 4 )

Other reasons

(n= 12 )

**Allocation**

**Analysis**

**Follow-Up**

**Enrollment**

Allocated to intervention

(n=50 )

Received allocated intervention

(n=50 )

Did not receive allocated intervention

(n=0 )

Give reasons

Is it Randomized? Yes

Analyzed (n=49)

Excluded from analysis (n= 1)

Give reasons: moved abroad (see above)

Lost to follow-up (n= 1)

Give reasons: Moved abroad

Discontinued intervention

(n= 0)

# Give reasons

Allocated to intervention

(n=50)

Received allocated intervention

(n= 50)

Did not receive allocated intervention

(n= 0)

Give reasons

Lost to follow-up (n= 3)

Give reasons

Discontinued intervention

(n= 2)

Give reasons: 2 drop-outs after 1^st^ treatment due to no time/interest

Analyzed (n=47)

Excluded from analysis (n=3)

Give reasons: 2 drop-outs (see above), and 1 was withdrawed due to non-compliant data for primary outcome.

**Reference List**
